# Comprehensive Genome-Wide Identification, Characterization, and Expression Analysis of *CCHC-*Type Zinc Finger Gene Family in Wheat (*Triticum aestivum* L.)

**DOI:** 10.3389/fpls.2022.892105

**Published:** 2022-04-29

**Authors:** Aolong Sun, Yongliang Li, Yang He, Xiaoxiao Zou, Fenglin Chen, RuiZhao Ji, Changqiao You, Keyao Yu, You Li, Wenjun Xiao, Xinhong Guo

**Affiliations:** College of Biology, Hunan University, Changsha, China

**Keywords:** wheat, *CCHC-ZFP* genes, evolution, abiotic stress, expression analyses

## Abstract

The CCHC-type zinc finger proteins (CCHC-ZFPs) play versatile roles in plant growth, development and adaptation to the environment. However, little is known about functions of *CCHC-ZFP* gene family memebers in *Triticum aestivum*. In the present study, we identified a total of 50 *TaCCHC-ZFP* genes from the 21 wheat chromosomes, which were phylogenetically classified into eight groups based on their specific motifs and gene structures. The 43 segmentally duplicated *TaCCHC-ZFP* genes were retrieved, which formed 36 segmental duplication gene pairs. The collinearity analyses among wheat and other eight mono/dicots revealed that no gene pairs were found between wheat and the three dicots. The promoter analyses of the *TaCCHC-ZFP* genes showed that 636 environmental stress-responsive and phytohormone-responsive *cis*-elements. The gene ontology enrichment analysis indicated that all the *TaCCHC-ZFP* genes were annotated under nucleic acid binding and metal ion binding. A total of 91 MicroRNA (miRNA) binding sites were identified in 34 *TaCCHC-ZFP* genes according to the miRNA target analysis. Based on the public transcriptome data, the 38 *TaCCHC-ZFP* genes were identified as differentially expressed gene. The expression profiles of 15 *TaCCHC-ZFP* genes were verified by the quantitative real-time PCR assays, and the results showed that these genes were responsive to drought or heat treatments. Our work systematically investigated the gene structures, evolutionary features, and potential functions of *TaCCHC-ZFP* genes. It lays a foundation for further research and application of *TaCCHC-ZFP* genes in genetic improvement of *T. aestivum*.

## Introduction

The CCHC-type zinc finger proteins (CCHC-ZFPs) are one of the largest transcription factors in plants, which play versatile roles in a variety of physiological processes. The CCHC-ZFPs regulate the expression of their target genes by directly or indirectly recognizing and binding the promoters (Takatsuji, 1999; Kiełbowicz-Matuk, 2012). As one type of zinc finger proteins, the CCHC-ZFPs contain at least one CCHC motif, which is also called zinc knuckle, sharing the consensus sequence CX_2_CX_4_HX_4_C (X for any amino acid, numbers for the number of residues, C and H for cysteine and histidine, respectively; Summers, 1991). The CCHC motifs with high affinity for DNA and RNA usually consist of a short helix and two short β-strands joined through a zinc knuckle, which function during transcriptional activation, RNA packaging, DNA recognition, and regulation of apoptosis (Laity et al., 2001; Wang et al., 2021). The first CCHC-ZFP was identified in the murine leukemia virus and Rous avian sarcoma virus, and subsequently in antigen proteins of retroviral nucleocapsids and eukaryotic retrotransposons (Summers, 1991). Comprehensive studies of *CCHC-ZFP* gene family were carried out in humans (34), *Arabidopsis* (69), and yeast (7), while the CCHC-ZFPs in wheat have not been reported so far (Aceituno-Valenzuela et al., 2020).

The *CCHC-ZFP* genes were extensively founded in plant genomes, which played central roles in seed development, and plant growth mediated by phytohormones, such as indole-3-acetic acid (IAA), gibberellins (GA), abscisic acid (ABA), and methyl jasmonate (MeJA). In rice, *OsZFP* regulates lateral root development via IAA signaling pathways (Cui et al., 2017). *AtCSP2* negatively modulates seed germination by adjusting GA and ABA contents (Sasaki et al., 2015). Additionally, a great number of *CCHC-ZFP* genes also participate in regulating plant tolerance to environmental stress. Overexpressing *OsZFP6*, a NaCl, H_2_O_2_, and NaHCO_3_ responsive gene, increases the tolerance to H_2_O_2_ and NaHCO_3_ in *Arabidopsis* (Guan et al., 2014). Similarly, *BrCSDP3* is a positive regulator of seed germination and seedling growth during dehydration and salinity treatment (Choi et al., 2015). The transcription of *OsRZ1, OsRZ2*, and *OsRZ3* is upregulated by low temperature treatment, but they show no response to high salinity and drought stress (Kim et al., 2010). In Pak-choi, *BcCSP1* plays a key role in responses to cold and ABA treatments (Huang et al., 2016). Besides, some *CCHC-ZFP* genes also participate in the regulation of plant defense to biotic stress (Amorim et al., 2017). Ectopic expression of wheat *TaRZ1* in *Arabidopsis* confers the transgenic plants enhanced resistance against bacterial (Xu et al., 2015). The up-regulation of *AdRSZ21* under MeJA treatment and pathogen infection indicates that *CCHC-ZFP* genes might take part in plant defense (Kumar and Kirti, 2012).

Bread wheat (*Triticum aestivum* L., A, B, and D sub-genome) was obtained by natural hybridization between *Triticum dicoccoides* (A and B sub-genome) and *Aegilops tauschii* (D sub-genome), which was a valuable material for evolutionary research due to the specificity of heterohexaploid (Ozkan et al., 2001; Petersen et al., 2006). The growth and development of wheat are susceptible to complex and variable environments, leading to the reduction of yield (Fujita et al., 2006). Considering the roles of *CCHC-ZFP* genes in various biological processes, a comprehensive investigation of *TaCCHC-ZFP* gene family will contribute to wheat stress resistance breeding and gene function research. In this study, we identified the *CCHC-ZFP* genes by bioinformatic methods from wheat genome and analyzed the chromosomal location, subcellular localization, phylogenetic relationships, gene structures, proteins interaction network, and expression patterns of them. The promoter *cis*-elements and the MicroRNA (miRNA) potentially targeting *TaCCHC-ZFP* genes were predicted to study the transcriptional regulatory network. These works will provide the basis for further analyses and application of *CCHC-ZFP* genes in wheat.

## Materials and Methods

### Plant Materials and Abiotic Stress Treatments

Bread wheat cultivar Fielder was used throughout this study. Seeds were placed in Petri dishes with wet filter paper at 4°C for 5 days. Then, the germinated seedlings were cultured in an incubator at 22°C (8 h dark and 16 h light period, about 60–70 μmol⋅m^–2^⋅s^–1^, and 50% relative humidity) with half strength Murashige and Skoog liquid medium. Two-week seedlings of Fielder were treated by drought stress [20% (m/V) PEG-6000], heat stress (40°C), or combined drought and heat stress (20% PEG-6000 and 40°C) for 1 or 6 h, respectively, while the seedlings under normal growing conditions (22°C, watered) were used as a control. Leaves were collected at 1 and 6 h after treatments, frozen in liquid nitrogen immediately, and stocked at −80°C for further study. All the experiments were conducted in parallel, and three biological replications were performed for each timepoint.

### Data Retrieval and Identification of *CCHC* Genes

The protein sequences and reference genomes for all the species in this study were available from the Ensemble Plants^1^. To identify the *CCHC-ZFP* family members, the Hidden Markov Model (HMM) profile of CCHC conserved motif (PF00098) from Pfam^2^ was applied to search against all of the protein sequences through the HMMER with the *E*-value < 1e^–4^ (Wheeler and Eddy, 2013; Mistry et al., 2021). After cleaning out the redundant sequences, candidate genes were subjected to Simple Modular Architecture Research Tool (SMART)^3^ to further verify CCHC-ZFP members (Letunic et al., 2021). The theoretical isoelectric point (pI), aliphatic index (AI), molecular weight (MW), instability index, and grand average of hydropathicity (GRAVY) were calculated using the ExPasy site^4^ (Gasteiger, 2003). The subcellular localization of each CCHC-ZFP was forecast using the Cell-PLoc 2.0^5^ (Chou and Shen, 2010). The secondary structure of TaCCHC-ZFPs was predicted using SOPMA secondary structure prediction^6^ (Geourjon and Deléage, 1995).

### Sequence Analysis and Structural Characterization of the CCHC Proteins in Wheat

Multiple protein sequence alignment of the characterized CCHC-ZFPs was carried out via ClustalX2 (Larkin et al., 2007). Then, depending on the full-length protein sequence alignment, the phylogenetic tree was constructed using MEGA 7.0 with the neighbor-joining method based on Poisson model, 1000 bootstrap replications and pairwise deletion (Kumar et al., 2016). The MEME online program^7^ was applied to identify the conserved motifs of CCHC-ZFPs in wheat (Bailey et al., 2009). Then, the conserved motif of wheat CCHC was extracted and visualized by WebLogo^8^ (Crooks et al., 2004). The wheat genome annotation file (GFF3 file) of wheat was retrieved from the Ensemble Plants (see text footnote 1) for analyzing the exon-intron structures of *TaCCHC-ZFP* genes. Finally, the prepared files were imported into TBtools for visualizing the protein motifs and gene structures (Chen et al., 2020a).

### Chromosome Distribution, Collinearity Analysis, and Ka/Ks Analysis

According to the information of chromosome location obtained from the Ensemble Plants, the *TaCCHC-ZFP* genes were mapped into the wheat chromosome by MapGene2Chrom V2^9^ (Chao et al., 2015). Subsequently, the gene duplication events and synteny of wheat *CCHC-ZFP* genes were analyzed using MCScanX and DIAMOND with the default parameters, and the figure was displayed by the Circos (Krzywinski et al., 2009; Wang et al., 2012; Buchfink et al., 2015). Additionally, the collinearity relationships and segmental duplication events of *TaCCHC-ZFP* gene pairs from other species were also performed similarly. The species evolution tree was drawn by using TimeTree online tool^10^ (Kumar et al., 2017). Then, the TBtools was adopted to calculated Ks (synonymous) and Ka (non-synonymous) of the duplicated gene pairs for further estimating duplication events (Chen et al., 2020a). The time (*T*) of duplication in millions of years (Mya) was estimated with the formula *T* = Ks/2λ × 10^–6^ Mya (λ = 6.5 × 10^–9^).

### *Cis*-Acting Element Analysis and Gene Ontology Annotation of *TaCCHC* Family Genes

In order to investigate the *cis*-acting elements in the promoter of *TaCCHC-ZFP* genes, the 1.5-kb upstream genomic DNA sequences of the transcription start codon were submitted to the PlantCARE database^11^ (Lescot et al., 2002). Then, the Gene Structure Display Server (GSDS)^12^ was adopted to visualized the *cis*-element distribution (Hu et al., 2015). The gene ontology (GO) analysis of *TaCCHC* genes was predicted for functional annotation using Omicshare Tools^13^.

### Prediction of Protein Interaction Network and MicroRNA Targets

The wheat CCHC-ZFPs were committed to the STRING database^14^ to analyze the protein-protein interaction network with high confidence (0.700; Szklarczyk et al., 2019). Then, The Cytoscape was adopted to visualize the interaction network with default parameters (Shannon et al., 2003). To predict the miRNAs targeting *TaCCHC-ZFP* genes, mature miRNA sequences and *TaCCHC-ZFP* gene sequences of wheat were submitted to the psRNATarget tool^15^, filtered at an expectation level ≤ 5.0 (Dai et al., 2018).

### Expression Analyses of *TaCCHC* Genes

The gene expression patterns of *TaCCHC* under abiotic stresses were available from the expVIP^16^ (Liu et al., 2015; Ramírez-González et al., 2018). Subsequently, the edgeR package was performed to identified the differentially expressed genes (DEGs) with fold change ≥ 2 and *q*-value ≤ 0.5 (Robinson et al., 2009). The TBtools was applied to create the gene expression heatmap (Chen et al., 2020a). Finally, EVenn^17^ was adapted to construct Venn diagrams.

### RNA Extraction and Quantitative Real-Time PCR Analyses

The total RNA from wheat leaves was extracted using TRIzol reagent (Vazyme Biotech Co., Ltd). For quantitative real-time PCR (qRT-PCR) analyses, RNA concentration was assessed by the NanoDrop 2000 spectrophotometer (ND-2000, Thermo Fisher Scientific, Inc.). Total RNAs were reverse transcribed with the HiScript II 1st Strand cDNA Synthesis Kit (+gDNA wiper; Vazyme Biotech Co., Ltd). The expression of 15 stress-responsive *TaCCHC-ZFP* genes were examined by qRT-PCR analyses, while *TaRP15* was served as a reference gene. The reaction system was composed of 5 μL of 2 × ChamQ Universal SYBR qPCR Master Mix (Vazyme Biotech Co., Ltd), 2 μL of template, 0.2 μL of each prime, and 2.6 μL of ddH_2_O. The reaction was performed as follows: pre-denaturation at 95°C for 30 s (step 1), denaturation at 95°C for 10 s (step 2), primer annealing/extension and collection of fluorescence signal at 60°C for 30 s (step 3). The next 40 cycles started at step 2. Each sample was performed in three biological replications and three technical replications. Subsequently, the data from qRT-PCR analyses was calculated with the 2^–ΔΔCT^ method. Primer sequences used in this study were listed in detail in Supplementary Table 10.

## Results

### Identification and Characterization of the *CCHC* Gene Family

In this study, a total of 50 putative *CCHC* genes in wheat were retrieved based on the HMMER search. After SMART searched, 50 wheat proteins sharing the CCHC conserved motifs were obtained, which were consistent to the predictions. Meanwhile, several important dicotyledonous and monocotyledonous plants were selected for reference analyses. We identified 38 *CCHC-ZFP* genes in *T. dicoccoides*, 46 *CCHC-ZFP* genes in *Ae. tauschii*, 17 *CCHC-ZFP* genes in *Hordeum vulgare*, 26 *CCHC-ZFP* genes in *Oryza sativa*, 33 *CCHC-ZFP* genes in *Zea mays*, 22 *CCHC-ZFP* genes in *Arabidopsis thaliana*, 95 *CCHC-ZFP* genes in *Glycine max*, and 67 *CCHC-ZFP* genes in *Solanum tuberosum* in the same method (Supplementary Table 1).

Subsequently, physicochemical properties of TaCCHC-ZFPs were analyzed, including the length of proteins, MW, AI, pI, instability index, GRAVY, and the subcellular localization (Supplementary Table 2). Among the 50 TaCCHC-ZFPs, TaCCHC14 is identified to be the smallest protein with 162 residues of amino acids (aa), while TaCCHC31 with 1,149 residues of amino acids is the largest one. The pI ranges from 5.31 (TaCCHC40) to 11.63 (TaCCHC41), and AI fluctuates from 20.06 (TaCCHC25) to 74.88 (TaCCHC5), and instability index varies from 25.67 (TaCCHC22) to 117.51 (TaCCHC41). Besides, the GRAVY values of all TaCCHC-ZFPs are negative, implying that TaCCHC-ZFPs may be hydrophilic proteins. Additionally, the subcellular localization predictions showed that 28 TaCCHC-ZFPs were located both in the cell nucleus and chloroplast, whereas 20 and 2 TaCCHC-ZFPs were only located in the nucleus or chloroplast, respectively. Additionally, all TaCCHC-ZFPs are composed of four secondary structures, including alpha helix (0.62–40.61%), extended strand (5.99–26.53%), beta turn (3.05–24.62%), and random coil (43.32–83.6%), of which random coil accounts for the main part of protein secondary structure (Supplementary Table 3).

**FIGURE 1:**
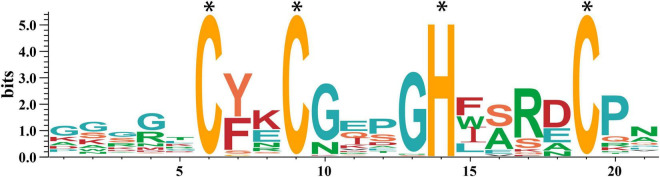
Sequence of the CCHC motifs in wheat. The height of the letter at each location (in bits) represents the conservation of the sequences, and the height of every single letter means the relative frequency of the corresponding amino acid of that position. The * represents the completely conserved residues.

Subsequently, we obtained the amino acid sequences of the conserved motif CCHCs using the MEME tool. As shown in Figure 1, the CCHC conserved motif from wheat has the consensus sequence CX_2_CX_4_HX_4_C, which has high affinity to nucleic acids. Except for the completely conserved histidine (H) and cysteine (C) residues in the positions 6, 9, 14, 19, the conserved substituted glycine (G) residue occurs in the positions 10, 13, and hydrophobic or aromatic residues are found in the positions 7, 15 (Figure 1).

### Phylogenetic Tree and Sequence Structure Analysis

**FIGURE 2:**
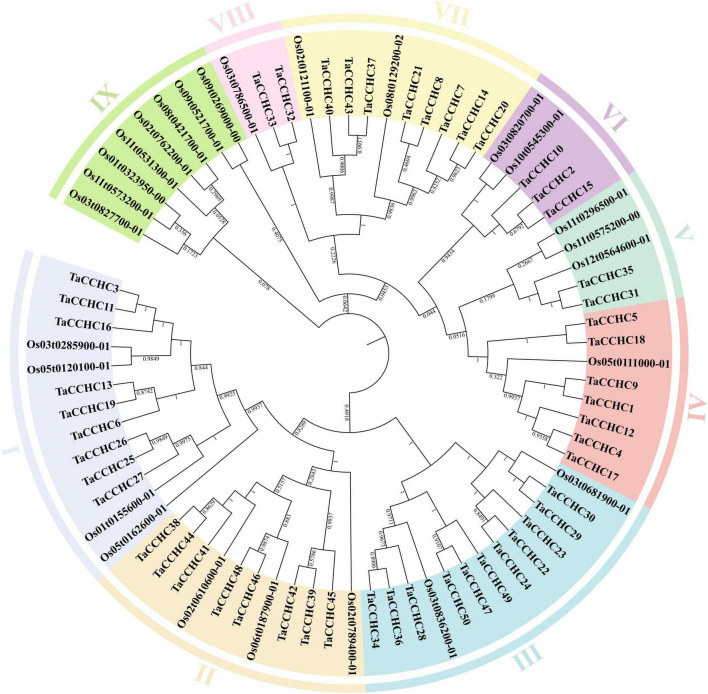
Phylogenetic tree of CCHC-ZFPs in wheat and rice. The protein sequences were aligned using the ClustaX2, and the Neighbor-joining (NJ) phylogenetic tree was built using the MEGA 7.0 based on Poisson model. Different groups are indicated by different colors, respectively.

**FIGURE 3:**
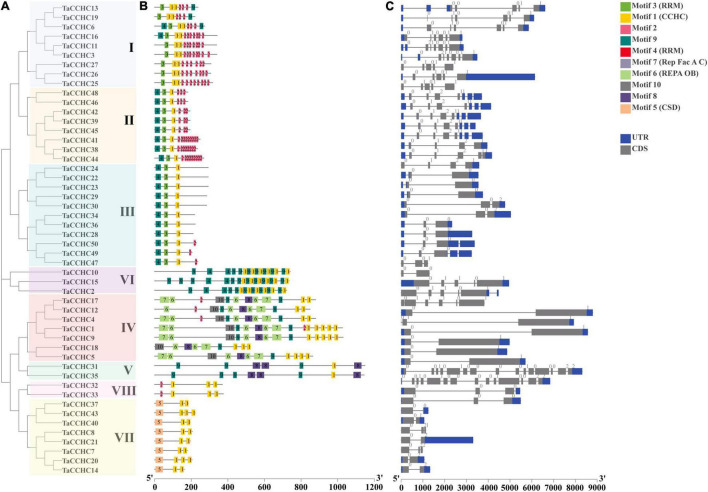
Comparative analyses of the phylogenetic relationships, protein conserved motifs, and gene structures of *CCHC-ZFP* family in wheat. **(A)** Phylogenetic tree of 50 TaCCHC-ZFPs was constructed by using MEGA 7.0. Each group was marked by a different color. **(B)** Motif composition of wheat CCHC-ZFPs. MEME was adopted to identify the conserved motifs of the TaCCHC-ZFPs. The motifs are displayed by different-colored boxes with the corresponding number in the center of the motifs. **(C)** Gene structures of *TaCCHC-ZFP* genes. The black lines represent the introns, while the blue and gray boxes represent the untranslated regions (UTRs) and exons, respectively. The numbers represent the phases of corresponding introns.

To study the evolutionary relationship of the *CCHC-ZFP* genes, a phylogenetic tree was constructed using the protein sequences of CCHC-ZFPs from both wheat and rice. These *CCHC-ZFP* genes are classified into nine groups, named as groups I to IX, which are distributed unevenly in each group (Figure 2). Except for the group IX, the others all possess *CCHC-ZFP* genes from both wheat and rice. The groups I and III both contain the most members of 13, and the group III is also the group with most members of 11 *CCHC-ZFP* genes in wheat. In addition, the group VIII possesses the fewest members, two from wheat and one from rice. Based on the phylogenetic analysis, *TaCCHC-ZFP* genes are classified into eight groups (groups I to VIII) for further analyses (Figure 3A).

A schematic diagram displaying the motifs of TaCCHC-ZFPs was constructed with the MEME tool (Bailey et al., 2009). Through the annotations of the Pfam (PF00098) database, we found that the motif 1 was the CCHC domain, and the motif 3 and 4 both were RRM domains, and the motif 5, 6, and 7 were CSD domain, REPA OB domain, and Rep Fac-A C domain, respectively, (Supplementary Table 4; Mistry et al., 2021). As shown in Figure 3B, the motif 1 was extensively distributed in TaCCHC-ZFPs. Moreover, TaCCHC-ZFPs in one group generally tend to have a similar motif composition. For instance, the motif 5 only exists in the group VII, while the motifs 6, 7 and 10 are specific to the group IV. Similarly, the motifs 8 is unique to the group IV and V, and the motif 3 only occurs in the groups I, II, and III. As a result, the motif patterns of TaCCHC-ZFPs in a group are similar, suggesting that the protein structure is conserved within a specific group. The roles of the conserved motifs remain to be elucidated, which may be relevant to specific biological functions.

Additionally, the exon-intron structures of *TaCCHC-ZFP* genes were investigated to understand the evolution of the *TaCCHC-ZFP* family. The gene structures of *TaCCHC-ZFPs* in different groups are changeable in the number of exons (ranging from 2 to 15; Figure 3C). However, the *TaCCHC-ZFP* genes in the same group usually share similar numbers of exons as expected, suggesting that they are evolutionarily conserved. For instance, all members of the group VII contain two or three exons, while seven *TaCCHC-ZFP* genes of the group II possess four exons. In contrast, some of the more closely related members were also observed to share similar length of exons. In general, the diverse gene structures of *TaCCHC-ZFP* genes may be related to the involvement of *TaCCHC-ZFP* genes in many plant biological processes.

### Chromosomal Location and Collinearity Analysis of the *TaCCHC* Genes

**FIGURE 4:**
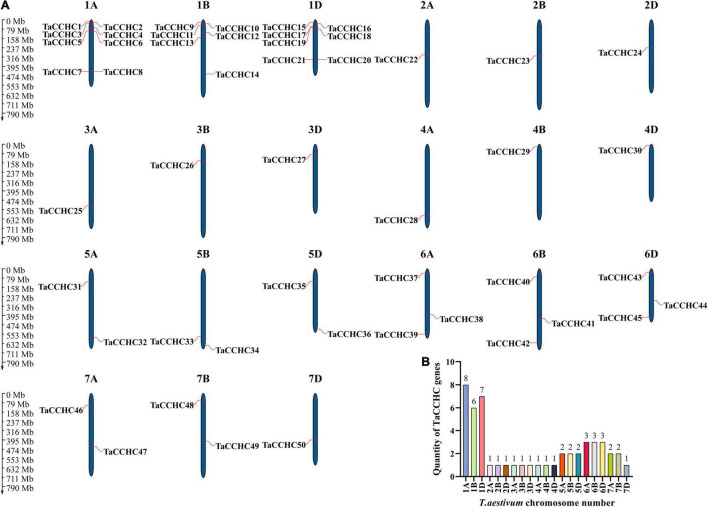
Chromosome distribution of the wheat *CCHC-ZFP* genes. **(A)** Chromosomal localization of the *TaCCHC-ZFP* genes. The dark blue columns indicate wheat chromosomes with the scale in megabases (Mb). The chromosome number is displayed at the top of each chromosome. **(B)** Numbers of *TaCCHC-ZFP* genes on each *T. aestivum* chromosome.

MapGene2Chrom V2 was adopted to create the chromosome map of the *TaCCHC-ZFP* genes based on the physical location information (Figure 4A; Chao et al., 2015). The *TaCCHC-ZFP* genes are unevenly spread across wheat chromosomes, with the number of the genes on each chromosome varying from one (2A, 2B, 2D, 3A, 3B, 3D, 4A, 4B, 4D, and 7D) to eight (1A; Figure 4B). Interestingly, we also found that the numbers of the *TaCCHC-ZFP* genes on each chromosome were not relevant to chromosome size. For instance, the smallest chromosome (6D, 473.6 Mb) encodes three *TaCCHC-ZFP* genes, while the largest chromosome (3B, 830.8 Mb) contains only one *TaCCHC-ZFP* gene. The *TaCCHC-ZFP* genes spread roughly equally in the three sub-genomes of wheat (sub-genome A, 18; sub-genome B, 16; and sub-genome D, 16), which may cause redundant functions with genes on sub-genome A, indicating some *TaCCHC-ZFP* genes may experience gene loss event during the evolution with low purifying selection. We also found that the 50 *TaCCHC-ZFP*s formed 19 homoeologous groups, of which 12 homoeologous groups contained three homoeologous genes with strict 1: 1: 1 correspondence, while the other 7 homoeologous groups were referred as dyads (1: 1: 0, 1: 0: 1, 0: 1: 1; Figures 3, 4A). Meanwhile, the homoeologous genes, *TaCCHC28* (4A), *TaCCHC34* (5B), *TaCCHC36* (5D), were not located in the same homologous chromosomes, suggesting that the *TaCCHC-ZFPs* were involved in the structural rearrangements of the 4A-5A chromosomes during the evolution of wheat (Chen et al., 2020b).

**FIGURE 5:**
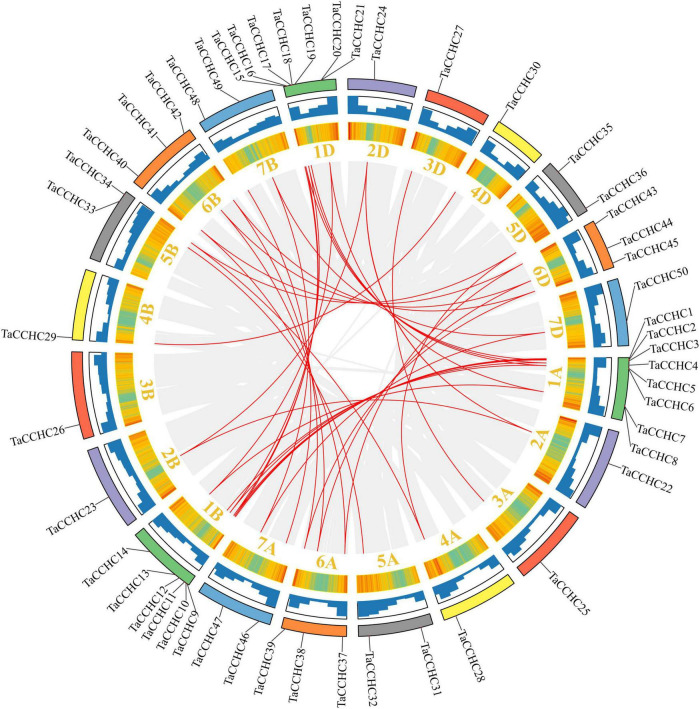
Synteny analysis of the *CCHC-ZFP* family in wheat. The gray lines indicate all synteny blocks within the wheat genome, while the red lines represent duplicated *CCHC-ZFP* gene pairs. Wheat chromosomes are displayed by rectangles with different colors, and the heatmaps and histograms along the rectangles show the gene density of each chromosome.

Next, the synteny analyses were performed to evaluate the gene duplication events in *T. aestivum.* Interestingly, we didn’t identify any tandem duplication events in these *TaCCHC-ZFP* genes. Nevertheless, a total of 36 segmental duplication events with 43 *TaCCHC-ZFP* genes were identified, indicating that segmental duplication events were the major driver for the evolution of *TaCCHC-ZFP* genes (Figure 5). All duplicated genes in a pair belong to the same *TaCCHC-ZFP* genes group. Furthermore, the Ka/Ks values of the *TaCCHC-ZFP* gene pairs were computed to explore the evolutionary constraints. The Ka/Ks values of the 36 gene pairs in wheat are generally less than 1, implying that the replicated *TaCCHC-ZFP* genes could experience strong purification selection pressure (Supplementary Table 5). The Ks values were adopted to assess the divergence time (*T*) based on the formula *T* = Ks/2λ × 10^–6^ Mya (λ = 6.5 × 10^–9^). The divergence time of these genes diverged between 0.994 and 19.055 Mya (average 6.735, 34 values in 36 earlier than 2.26), mostly before the early Gramineae whole-genome duplication event.

### Synteny Analyses of *CCHC* Members From Wheat and Eight Other Plant Species

**FIGURE 6:**
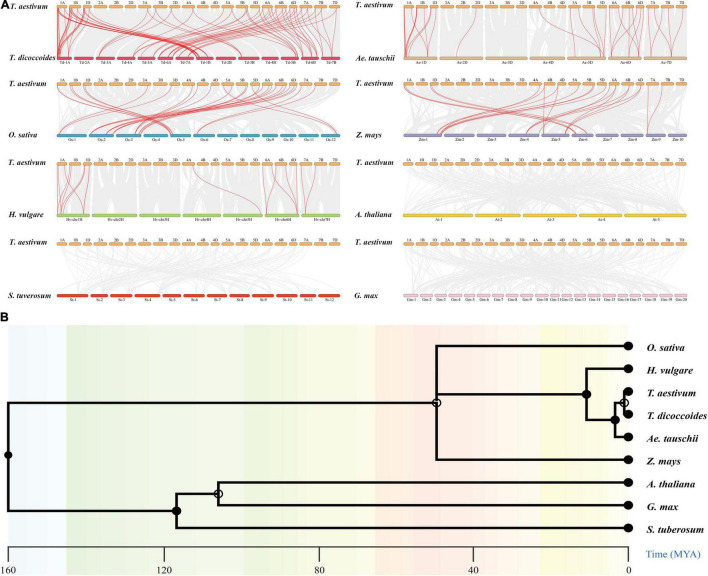
Synteny analyses of the *CCHC-ZFP* family between wheat and other species. **(A)** Collinearity analysis of the *TaCCHC-ZFP* family with other eight representative species. The gray lines in the background represent the collinear blocks in the genome of wheat and other species, while the red lines indicate the syntenic *CCHC-ZFP* gene pairs. **(B)** Species evolution tree of wheat and other eight species.

To further investigate the evolutionary mechanisms and homologous genes of *TaCCHC-ZFPs*, comparative syntenic maps were constructed by comparing eight representative species with wheat, including five monocots (*T. dicoccoides*, *Ae. tauschii*, *Z. mays, O. sativa*, and *H. vulgare*) and three dicots (*A. thaliana*, *S. tuberosum*, and *G. max*; Figure 6A). A total of 46 *TaCCHC-ZFP* genes show collinearity relationships with 15 *CCHC-ZFP* genes in *Ae. tauschii*, 29 in *T. dicoccoides*, 12 in *O. sativa*, 8 in *H. vulgare*, and 8 in *Z. mays*, respectively, while no this relationship among wheat and the three dicots analyzed was found, suggesting the closer phylogenetic relationships with the monocots than the dicots (Figure 6B). Therefore, 31, 76, 28, 16, 17 orthologous gene pairs among wheat and *Ae. tauschii*, *T. dicoccoides*, *O. sativa*, *Z. mays*, and *H. vulgare* were identified, respectively, (Supplementary Table 6). Hexaploid wheat (A, B, and D sub-genome) was obtained by natural hybridization between *T. dicoccoides* (A and B sub-genome) and *Ae. tauschii* (D sub-genome). Compared to *T. dicoccoides* and *Ae. tauschii*, more wheat *CCHC-ZFP* genes were derived from *T. dicoccoides* based on the number of orthologous *CCHC-ZFP* gene pairs. Among the three sub-genomes of wheat, 36 gene pairs (14 between the A and B sub-genomes, 11 between the A and D sub-genomes, 11 between the B and D sub-genomes) were identified, which were less than that between wheat and the sub-genome donors (Figures 5, 6A). This might be related to either the gene lost or chromosomal recombination during the polyploidization and evolution. Additionally, three *TaCCHC-ZFP* genes (*TaCCHC37*, *TaCCHC46*, and *TaCCHC48*) were observed in all of five syntenic maps, indicating that these *TaCCHC* genes were relatively conserved in the evolution. However, some wheat *TaCCHC-ZFP* genes identified were collinear with genes from only one species. For instance, *TaCCHC35* was identified to have a collinearity relationship with *Os12t0564600-01*, while there was no collinearity with the *CCHC-ZFP* genes from the other four species, implying that *TaCCHC35* might have been lost in the rest four plants and remained in wheat and rice.

To further investigate the evolutionary constraints of the *TaCCHC-ZFP* genes, the Ka/Ks ratios of the *CCHC-ZFP* gene pairs were computed. The Ka/Ks ratios of nearly all orthologous *CCHC-ZFP* gene pairs were less than 1, indicating that the *TaCCHC-ZFP* genes might undergone purifying selection during the evolution to eliminate harmful mutations at the protein level (Supplementary Table 6). The divergence time of these duplicated orthologous *TaCCHC-ZFP* gene pairs were approximately 4.872 Mya (*T. dicoccoides*), 5.143 Mya (*Ae. tauschii*), 11.074 Mya (*H. vulgare*), 44.765 Mya (*O. sativa*), and 60.761 Mya (*Z. mays*), respectively, which were close to the result of the species evolution tree (Figure 6B).

### *Cis*-Acting Elements and Gene Ontology Enrichment Analyses of *TaCCHC-ZFP* Genes

**FIGURE 7:**
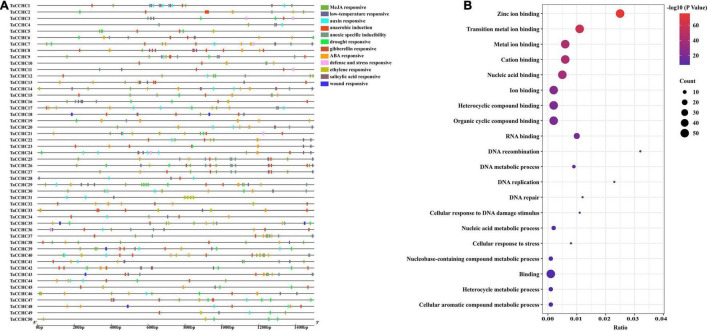
Analyses of *cis*-acting elements and functional annotation of *TaCCHC-ZFP* genes. **(A)** Distribution of predicted *cis*-acting elements in the promoter regions of *TaCCHC-ZFP* genes. The color blockers indicate different *cis*-acting elements and their locations in these *TaCCHC-ZFP* genes. **(B)** GO enrichment analysis of the *TaCCHC-ZFP* genes.

Transcription factors bind the *cis*-acting elements of the promoter regions to regulate transcription. Thus, the 1.5-kb upstream promoter regions of all *TaCCHC-ZFP* genes were submitted to the PlantCARE to study the potential biological functions of *TaCCHC-ZFP* genes. A total of 636 *cis*-elements associated with environmental stress signal and phytohormone responsiveness were found in the promoter regions of *TaCCHC-ZFP* genes (Figure 7A and Supplementary Table 7). Among them, 152 MeJA-responsive elements (TGAGG-motif and CGTCA-motif) and 128 ABA-responsive elements (ABRE) were found, respectively, which were the two most *cis*-acting elements of *TaCCHC-ZFP* genes. The result suggested that MeJA and ABA might take part in the transcriptional regulation of *TaCCHC-ZFP* genes. Moreover, 40 auxin-responsive *cis*-acting elements (AuxRR-core, TGA-element), 40 ethylene-responsive elements (ERE), 27 gibberellin-responsive elements (P-box, GARE-motif, and TATC-box), 16 salicylic acid-responsive elements (SARE and TCA-element) were identified in 40, 27, 16, and 14 *TaCCHC-ZFP* genes, respectively. Meanwhile, four types of *cis*-elements associated with biotic or abiotic stress responsiveness were identified, such as 45 drought responsive elements (MBS), 60 low-temperature responsive elements (LTR), 8 wound responsive elements (WUN-motif), 9 defense and stress responsive elements (TC-rich repeats). Additionally, except for *TaCCHC31*, *TaCCHC44*, *TaCCHC49*, and *TaCCHC50*, the anaerobic induction (ARE) or anoxic specific inducibility element (GC-motif) were found in the rest 46 *TaCCHC-ZFP* genes. In brief, the *cis*-acting elements identified in the promoter regions indicate that *TaCCHC-ZFP* genes may participate in the transcriptional regulation of phytohormone signaling and biotic/abiotic stress responses.

Additionally, the GO enrichment of all *TaCCHC-ZFP* genes was constructed to further explore the gene functions. The GO terms consist of three categories: cellular component, biological process (BP), and molecular function (MF). The enrichment results of the MF category revealed that all 50 *TaCCHC-ZFP* genes were annotated under nine GO terms, including zinc ion binding, metal ion binding, transition metal ion binding, cation binding, nucleic acid binding, ion binding, heterocyclic compound binding, organic cyclic compound binding, and binding, all of which belonged to the MF category, suggesting that they might act as zinc finger proteins to regulate gene expression through DNA or RNA binding (Figure 7B). The enrichment results of the BP category revealed that 12 *TaCCHC* genes participated in four kinds of metabolic processes, such as nucleic acid metabolic process and cellular aromatic compound metabolic process. Moreover, seven *TaCCHC-ZFP* genes shared five GO terms, which were DNA recombination, DNA replication, cellular response to DNA damage stimulus, DNA repair, and cellular response to stress, implying the potential roles of them during stress responses.

### Protein Interaction Network and MicroRNA Targets Analysis

**FIGURE 8:**
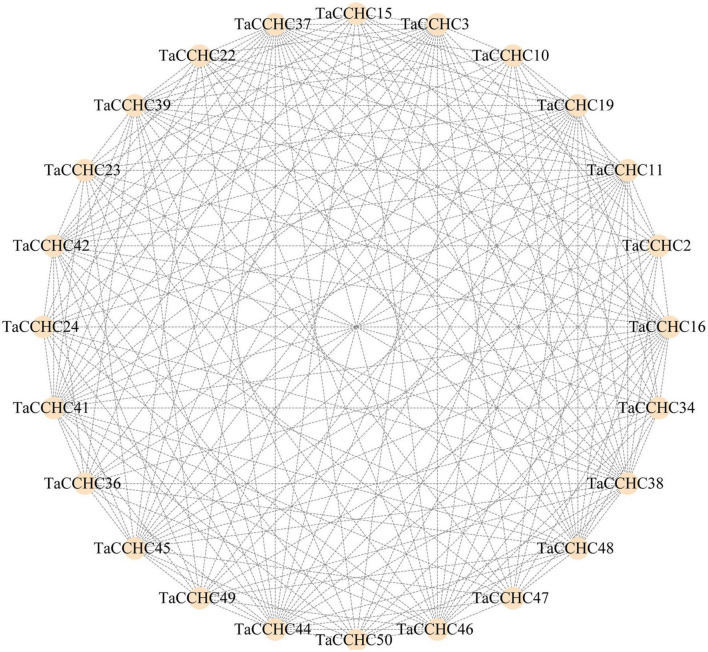
Interaction network of TaCCHC-ZFPs. A total of 202 interactions are displayed among 24 TaCCHC-ZFPs. The protein-protein interaction networks of wheat CCHC-ZFPs were predicted using the STRING tools with high confidence (0.700; Szklarczyk et al., 2019), and was used to visualized by the Cytoscape with default parameters (Shannon et al., 2003).

Proteins that perform similar functions or participate in the same pathway are more likely to exhibit interaction networks, forming gene modules or clusters in proteins interaction networks. To further understand the interaction relationships and biological functions among TaCCHC-ZFPs, the STRING database was adopted to map the protein-protein networks within TaCCHC-ZFP family. As shown in Figure 8, 24 TaCCHC-ZFPs were found to be involved in the protein interaction networks with 202 branches, suggesting that they might perform similar function.

MicroRNAs are small non-coding RNAs that function in RNA silencing and post-transcriptional regulation of gene expression. Thus, the potential miRNA targets of *TaCCHC-ZFP* genes were predicted to provide support information about the regulatory mechanism of the *TaCCHC-ZFP* genes. The results revealed that a total of 91 miRNA target sites were identified in 34 *TaCCHC-ZFP* genes, with each gene corresponding to one to seven miRNAs (Supplementary Table 8). Among the 34 wheat *CCHC-ZFP* genes, *TaCCHC36* and *TaCCHC50* had the most targets with seven miRNA target sites, followed by *TaCCHC21* and *TaCCHC26* with six miRNA target sites, implying that the expression of *TaCCHC-ZFP* genes might be regulated by multiple miRNAs. At the same time, tae-miR9652-5p had the most target sites (nine *TaCCHC-ZFP* genes) among the 47 wheat miRNAs, followed by tae-miR9782 targeting six genes.

### Expression Patterns of *TaCCHC* Genes Under Different Stresses

**FIGURE 9:**
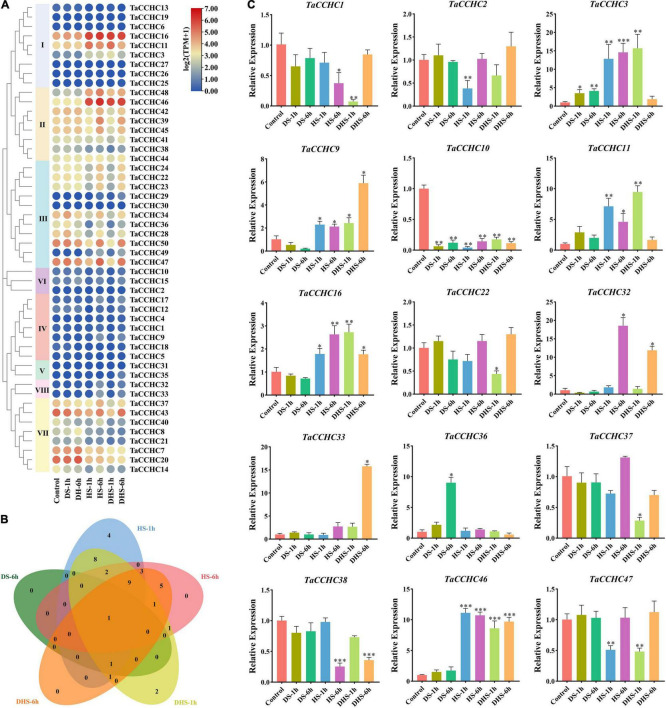
Expression profiles of wheat *CCHC-ZFP* genes under different conditions. **(A)** Expression profiles of 50 *TaCCHC-ZFP* genes under different stress treatments. HS-1 (6) h: heat stress for 1 (6) h; DS-1 (6) h: drought stress for 1 (6) h; DHS-1 (6) h: combined drought and heat stress for 1 (6) h. The color in the heat map reflects *TaCCHC-ZFP* genes expression level. **(B)** Venn diagrams of DEGs under different treatments. **(C)** Expression analyses of 15 *TaCCHC-ZFP* genes in response to different treatments by qRT-PCR. Data were normalized to *actin-TaRP15* and error bars represent standard deviation among three independent replicates (*P < 0.05, ^**^P < 0.01, ^***^P < 0.001, and Student’s *t*-test).

To further dissect the function of *TaCCHC* genes under abiotic stresses, the expression profiles of 50 *TaCCHC-ZFP* genes during different treatments (drought, heat, drought, and heat) were analyzed in this study (Figure 9A). Among the 50 genes, 38 DEGs were screened out via the edgeR package from five kinds of treatments [drought stress for 1 (6) h: DS-1 (6) h, heat stress for 1 (6) h: HS-1 (6) h, and combined drought and heat stress for 1 (6) h: DHS-1 (6) h], while no DEGs were identified under the DS-1h treatment (Supplementary Table 9). As shown in Figure 9B, 32 *TaCCHC-ZFP* genes responded to at least two treatments, while six *TaCCHC-ZFP* genes only responded to one treatment. For instance, *TaCCHC34, TaCCHC42*, and *TaCCHC47* showed decreased expression under the HS-1h and DHS-1h treatments, while *TaCCHC11* exhibited increased expression under the HS-1h and DHS-1h treatments, implying that these genes were sensitive to heat and drought. As expected, some genes with close evolutionary relationships showed similar expression patterns. The expression of *TaCCHC22*, *TaCCHC23*, and *TaCCHC24* were downregulated under the HS-1h and DHS-1h treatments, while the expression of *TaCCHC32* and *TaCCHC33* were upregulated under the HS-6h and DHS-6h treatments. Additionally, the expression patterns of *TaCCHC-ZFP* genes under cold and phosphorous starvation treatments were also analyzed (Supplementary Figure 1). Only eight *TaCCHC-ZFP* genes responded to cold treatment, while no DEGs were identified under the phosphorous starvation treatment (Supplementary Table 9).

To confirm the transcription profiles of the *TaCCHC-ZFP* genes derived from the transcriptome data, 15 *TaCCHC-ZFP* genes from the eight groups were selected to analyze their expression level under different treatments by qRT-PCR. As shown in Figure 9C, the expression profiles of most *TaCCHC-ZFP* genes are congruent with the previously published data according to the results of qRT-PCR. Overall, the expression of *TaCCHC-ZFP* genes could be influenced by multiple treatments.

## Discussion

As one of the most important food crops, wheat is subject to environmental stresses, resulting in the reduction of yield. Zinc finger protein transcription factors play vital roles in plant growth and development, and biotic and abiotic stress responses (Li et al., 2013). Some previous studies revealed that *TaCCHC-ZFP* genes regulated plant growth and stress responses, such as *AtRZ-1a*, *Mt-Zn-CCHC*, and *NTT* (Kim et al., 2005; Crawford et al., 2007; Lee and Kang, 2016; Radkova et al., 2019). Thus, the comprehensive bioinformatic analyses of *TaCCHC-ZFP* gene family were conducted to better study the gene functions of *TaCCHC-ZFP* genes due to the limited work on *TaCCHC-ZFP* gene family.

In this study, we identified 50 TaCCHC-ZFPs from wheat and extracted CCHC motif sequences of these members, in which the conserved sites were consistent with the previous study (Figure 1; Armas and Calcaterra, 2013). Studies revealed that CCHC motif was a kind of nucleic acid binding domain, which contributed to RNA binding, DNA regulation, or protein-protein interactions (Matthews et al., 2000; Espinosa, 2003; Ganie, 2020). Meanwhile, the results of GO enrichment showed that all 50 *TaCCHC-ZFP* genes were annotated under nucleic acid binding term and zinc ion binding term (Figure 7B), implying that TaCCHC-ZFPs might function by binding DNA or RNA. Previous study showed that WCSP1 (TaCCHC7 in this study) was capable of binding dsDNA, ssDNA, and RNA homopolymers, whereas its ability to bind dsDNA was almost eliminated in the absence of *C*-terminal CCHC motif (Karlson et al., 2002). In *Arabidopsis*, CSDP1, homologous to TaCCHC14, which possesses seven tandem repeated CCHC motifs in the *C*-terminal half, acts as an RNA chaperone in the response to cold stress, helping to export mRNA from the nucleus to the cytoplasm (Park et al., 2009).

The analyses of phylogenetic relationships, protein motifs, and gene structures showed that the homologous *TaCCHC-ZFP* genes in sub-genomes A, B, D shared similar gene structures and conserved motifs, indicating the functions of *TaCCHC-ZFP* genes were conservative during the evolution (Figures 3A–C). The motif 1 (CCHC motif) is conserved in all TaCCHC-ZFPs. It is noteworthy that some motifs are distributed in specific groups, such as RRM, CSD, REPA OB, and Rep Fac-A C, which may participate in various biological processes based on the different functions of *TaCCHC-ZFP* genes (Supplementary Table 4; Bochkarev et al., 1997; Daubner et al., 2013; Budkina et al., 2020). For instance, AtGRP2 containing two CCHC zinc fingers and one CSD motifs may be involved in cold-response and flower development (Fusaro et al., 2007). Besides, RRM exists in groups I, II, and III, which can bind single-strand RNA and participate in the regulation of flowering and adaptation to heat stress (Pi et al., 2018). AtSF1, a protein containing RRM, takes part in regulating heat stress response by affecting the alternative splicing of the pre-mRNA of the heat shock transcription factor HsfA2 (Lee et al., 2017). Meanwhile, to better understand the homoeologous relationships, we analyzed homoeologous groups of wheat *TaCCHC-ZFP*s in detail (Figures 3, 4A). Approximately 36% of wheat genes are presented in homoeologous groups of three (1: 1: 1), while the remaining 64% have a more complex homologous relationship (e.g., 1: 1: 0 or 1: 1: N; Glover et al., 2016; Juery et al., 2021). By contrast, about 63% of wheat *TaCCHC-ZFP* genes identified are presented as triads, which is considerably above the average homoeologous retention rate in wheat (36%). On the other hand, the loss of one homoeolog is less in *TaCCHC-ZFP* genes (37 vs 64%), suggesting that the high homoelog retention rate could partly explain the abundance of *TaCCHC-ZFP* genes.

Previous researches revealed that gene families normally experienced tandem duplication events or segmental duplication events to expand gene family members in the process of evolution (Cannon et al., 2004). Subsequently, syntenic analyses were carried out in this study (Figures 5, 6A). Wheat has undergone two major polyploid evolutionary events, accompanied by segmental duplication, tandem duplication, and transposition events (Peng et al., 2011). However, the number of *TaCCHC-ZFP* genes in a specific sub-genome was severely reduced during the transition from tetraploid to hexaploidy through the identification of *CCHC-ZFP* genes in wheat and its sub-genomes donors, *T. dicoccoides* and *Ae. tauschii* (for A sub-genome, from 20 to 18 genes; B sub-genome, from 18 to 16 genes; D sub-genome, from 46 to 16 genes), proving that gene loss during hexaploidy wheat formation occurred extensively (Berkman et al., 2013). Generally, the Ka/Ks ratios for all the homologous *CCHC* gene pairs are less than 1, indicating that *TaCCHC-ZFP* genes may have undergone purifying selection pressure and the functions of these gene pairs do not diverge much after the two polyploidization events (Supplementary Tables 5, 6).

*Cis*-acting elements and miRNAs are involved in the regulation of gene expression at the transcriptional and post-transcriptional levels, respectively, (Hausser and Zavolan, 2014; Hernandez-Garcia and Finer, 2014). Therefore, we predicted the *cis*-acting elements in the promoter regions of wheat *CCHC-ZFP* genes and miRNAs targeting *TaCCHC* genes. Plenty of studies showed that *cis*-elements were essential factors of modulating gene expression under biotic and abiotic stress. For instance, *PbrMYB21* could interact with the MYB-recognizing *cis*-element in the promoter region of *PbrADC* to modulate polyamine synthesis via regulating *ADC* expression, improving drought tolerance (Li et al., 2017). In this study, a lot of *cis*-acting elements associated with environmental stress and phytohormone responsiveness were identified, indicating that *TaCCHC-ZFP* genes might take part in multiple signaling pathways (Figure 7A and Supplementary Table 7; Liu et al., 2014). Additionally, plant miRNAs are associated with cell biology processes and response to stress, which can regulate gene expression at the post-transcriptional level through splicing mRNA or inhibiting translation. In this study, we found 47 wheat miRNAs target with 34 *TaCCHC-ZFP* genes, including tae-miR9652-5p, tae-miR9782, tae-miR156, tae-miR159a/b, tae-miR164, and tae-miR167, etc. (Supplementary Table 8). Previous studies reported that some plant miRNAs, such as miR156, miR159a/b, miR164, miR319, and miR399, played an important role in modulating plant developmental time, the differentiation of tissues, and response to environmental stresses (Willmann and Poethig, 2007). The miR156-overexpression alfalfa showed significant improvement in drought tolerance with reduced water loss and higher survival compared with the wild-type control (Arshad et al., 2017). Moreover, ABA induced the accumulation of miR159 to mediate the cleavage of *MYB33* and *MYB101* transcripts in geminating *Arabidopsis* seeds (Reyes and Chua, 2007). In brief, *cis*-acting elements and miRNAs may be regulators of *TaCCHC-ZFP* gene expression.

Previous studies showed that *CCHC-ZFP* genes responded to multiple stresses. For example, the cold resistance of atRZ-1a-overexpressing transgenic *Arabidopsis* plants was enhanced compared to wild-type plants, with earlier germination and better seedling growth under cold treatment as well (Kim et al., 2005). Drought and heat are the main environmental stresses affecting wheat growth and development, often resulting in the decline of wheat yield. In this study, we investigated the potential functions of *TaCCHC-ZFP* genes under drought and heat treatments, and 38 DEGs were screened out (Figures 9A–C and Supplementary Table 9). Previous research revealed that AtCSP3-overexpressing transgenic *Arabidopsis* plants exhibited higher survival rates under the drought and salt treatment, whereas the *atcsp3* mutant displayed lower survival rate (Kim et al., 2013). *TaCCHC20*, homologous to *Arabidopsis At4g36020.1* (*AtCSP3*), was downregulated under the DHS-6h treatment, indicating that they might have similar functions under the drought and heat stresses. *TaRZ2* (*TaCCHC49* in this study) can negatively regulate seed germination and seedling growth under the salt or dehydration treatments but contribute to enhancing cold tolerance of transgenic *Arabidopsis* (Xu et al., 2014). Meanwhile, *TaCCHC20* and *TaCCHC49* were found to share similar *cis*-elements, such as TGA-element, LTR, and so on. Overall, these results suggested that *TaCCHC-ZFP* genes might be involved in the plant responses to drought and heat stresses.

## Conclusion

CCHC-ZFPs are involved in multiple physiological processes, such as seed development, plant growth, and responses to biotic and abiotic stresses. In this study, a total of 50 *TaCCHC-ZFP* genes were identified from wheat by bioinformatics tools. Subsequently, these *TaCCHC-ZFP* genes were categorized into eight groups with specific motifs and gene structures. Interestingly, only segmental duplication events were identified in *TaCCHC-ZFP* genes, suggesting that the segmental duplication events were the major driver for *TaCCHC-ZFP* genes evolution. In addition, collinearity relationships among wheat and eight other representative plants were analyzed and no gene pairs were identified between wheat and the three dicots. Plenty of *cis*-acting elements related to environmental stress were found in the promoters of *TaCCHC-ZFP* genes. GO enrichment results showed that all *TaCCHC-ZFP* genes were annotated under nucleic acid binding and metal ion binding. The analyses of miRNA targets suggested that the *TaCCHC-ZFP* genes could be regulated by the miRNAs. Furthermore, the expression patterns of *TaCCHC-ZFP* genes and qRT-PCR verification showed that some *TaCCHC-ZFP* genes participated in the responses to drought and heat stresses.

## Data Availability Statement

The datasets presented in this study can be found in online repositories. The names of the repository/repositories and accession number(s) can be found in the article/Supplementary Material.

## Author Contributions

XG and WX designed the experiments. AS and XG wrote the main manuscript text. YLL and AS conducted the experiments. AS, YLL, YH, XZ, FC, RJ, CY, KY, and YL collected and analyzed phenotype data. AS, YL, and WX prepared Figures 1–9. All authors read and approved the manuscript.

## Conflict of Interest

The authors declare that the research was conducted in the absence of any commercial or financial relationships that could be construed as a potential conflict of interest.

## Publisher’s Note

All claims expressed in this article are solely those of the authors and do not necessarily represent those of their affiliated organizations, or those of the publisher, the editors and the reviewers. Any product that may be evaluated in this article, or claim that may be made by its manufacturer, is not guaranteed or endorsed by the publisher.

## Footnotes

1
http://plants.ensembl.org/index.html

2
http://pfam.xfam.org

3
http://smart.emblheidelberg.de/

4
http://web.expasy.org/protparam/

5
http://www.csbio.sjtu.edu.cn/bioinf/plant-multi/

6
https://npsa-prabi.ibcp.fr/cgi-bin/npsa_automat.pl?page=npsa_sopma.html

7
https://meme-suite.org/meme/tools/meme

8
http://weblogo.threeplusone.com/

9
http://mg2c.iask.in/mg2c_v2.0

10
http://www.timetree.org

11
http://bioinformatics.psb.ugent.be/webtools/plantcare/html

12
http://gsds.gao-lab.org

13
https://www.omicshare.com

14
https://string-db.org

15
https://www.zhaolab.org/psRNATarget/

16
http://www.wheat-expression.com

17
http://www.ehbio.com/test/venn/#/

## Abbreviations

CCHC-ZFP: CCHC-type zinc finger protein
GA: gibberellins
IAA: indole-3-acetic acid
ABA: abscisic acid
MeJA: methyl jasmonate
MW: molecular weight
AI: aliphatic index
pI: isoelectric point
GRAVY: grand average of hydropathicity
aa: amino acids
REPA OB: Replication protein A OB
Rep Fac-A C: Replication factor-A C terminal domain
Ka: Non-synonymous
Ks: Synonymous
Mya: Mya millions of years
SA: salicylic acid
BP: biological process
MF: molecular function
CC: cellular component
miRNA: MicroRNA
DS-1 (6) h: drought stress for 1 (6) h
HS-1 (6) h: heat stress for 1 (6) h
DHS-1 (6) h: combined drought and heat stress for 1 (6) h
DEG: differentially expressed gene
qRT-PCR: quantitative real-time PCR
HMM: Hidden Markov Model
NJ: neighbor-joining
SMART: Simple Modular Architecture Research Tool
GSDS: Gene Structure Display Server
GO: gene ontology
UTR: untranslated regions.
