# Supplementary material for: Comprehensive Genome-Wide Identification, Characterization, and Expression Analysis of CCHC-Type Zinc Finger Gene Family in Wheat (Triticum aestivum L.)
Source: Front Plant Sci. 2022 Apr 29;13:892105. doi: 10.3389/fpls.2022.892105 (PMC9100697; doi:10.3389/fpls.2022.892105)
Supplement: Supplementary file 1 [file Data_Sheet_1.DOC]

**Supplementary Table 1.** List of CCHC-ZFPs identified in this study.

| ***Triticum dicoccoides*** | ***Aegilops tauschii*** | ***Hordeum vulgare*** | ***Oryza sativa*** | ***Zea mays*** | ***Arabidopsis thaliana*** | ***Glycine max*** | ***Solanum tuberosum*** |
| --- | --- | --- | --- | --- | --- | --- | --- |
| TRIDC1AG003450.2 | AET5Gv21108500.4 | HORVU1Hr1G004910.1 | Os01t0155600-01 | Zm00001d002713_P001 | AT4G36020.1 | KRH60779 | PGSC0003DMT400068563 |
| TRIDC1AG004630.1 | AET1Gv20524700.5 | HORVU1Hr1G006890.1 | Os01t0323950-00 | Zm00001d002866_P001 | AT2G17870.1 | KRH51468 | PGSC0003DMT400068562 |
| TRIDC1AG006650.1 | AET1Gv20088100.10 | HORVU1Hr1G009950.10 | Os02t0121100-01 | Zm00001d004066_P001 | AT1G75560.1 | KRH69774 | PGSC0003DMT400096473 |
| TRIDC1AG012090.1 | AET1Gv20067600.13 | HORVU1Hr1G018380.1 | Os02t0610600-01 | Zm00001d007507_P001 | AT3G43590.1 | KRH08060 | PGSC0003DMT400009195 |
| TRIDC1AG015200.1 | AET7Gv21078400.5 | HORVU1Hr1G024060.10 | Os02t0762200-01 | Zm00001d007799_P001 | AT3G42860.1 | KRG89907 | PGSC0003DMT400068565 |
| TRIDC1AG015700.1 | AET6Gv20173700.1 | HORVU1Hr1G052910.1 | Os02t0789400-01 | Zm00001d011004_P001 | AT2G21060.1 | KRG89908 | PGSC0003DMT400068566 |
| TRIDC1AG031050.1 | AET1Gv20635200.11 | HORVU1Hr1G064210.1 | Os03t0285900-01 | Zm00001d011005_P001 | AT4G38680.1 | KRG89906 | PGSC0003DMT400068567 |
| TRIDC1AG039170.1 | AET5Gv20120400.2 | HORVU4Hr1G074650.1 | Os03t0681900-01 | Zm00001d012922_P002 | AT5G36240.1 | KRG89903 | PGSC0003DMT400070774 |
| TRIDC1BG001190.13 | AET5Gv20120600.1 | HORVU5Hr1G022740.7 | Os03t0786500-01 | Zm00001d012965_P001 | AT3G55340.1 | KRH17933 | PGSC0003DMT400007740 |
| TRIDC1BG004320.5 | AET5Gv20990500.2 | HORVU5Hr1G103450.1 | Os03t0820700-01 | Zm00001d013543_P001 | AT3G53500.1 | KRH17934 | PGSC0003DMT400007742 |
| TRIDC1BG006490.1 | AET5Gv20120700.1 | HORVU5Hr1G113000.1 | Os03t0827700-01 | Zm00001d014885_P001 | AT2G37340.3 | KRH17935 | PGSC0003DMT400015114 |
| TRIDC1BG014830.1 | AET1Gv20209300.3 | HORVU5Hr1G118010.1 | Os03t0836200-01 | Zm00001d017177_P001 | AT5G52380.1 | KRG89904 | PGSC0003DMT400015112 |
| TRIDC1BG019080.1 | AET3Gv20291700.8 | HORVU6Hr1G012510.1 | Os05t0111000-01 | Zm00001d018341_P001 | AT2G12880.1 | KRG91524 | PGSC0003DMT400037206 |
| TRIDC1BG036510.1 | AET1Gv20274000.4 | HORVU6Hr1G083840.1 | Os05t0120100-01 | Zm00001d019041_P001 | AT5G04280.2 | KRH60714 | PGSC0003DMT400059696 |
| TRIDC1BG044550.2 | AET1Gv20235700.1 | HORVU7Hr1G031320.1 | Os05t0162600-01 | Zm00001d021860_P001 | AT3G02820.2 | KRH24281 | PGSC0003DMT400015116 |
| TRIDC3AG016490.1 | AET7Gv20812500.1 | HORVU7Hr1G078140.1 | Os06t0187900-01 | Zm00001d028789_P001 | AT3G26420.1 | KRH24277 | PGSC0003DMT400004030 |
| TRIDC3AG036880.6 | AET5Gv20664100.1 | HORVU7Hr1G107520.1 | Os08t0129200-02 | Zm00001d029811_P001 | AT1G60650.4 | KRH24276 | PGSC0003DMT400061468 |
| TRIDC3AG037200.14 | AET4Gv20688300.2 |  | Os08t0421700-01 | Zm00001d033511_P001 | AT4G31580.1 | KRH24263 | PGSC0003DMT400061470 |
| TRIDC3BG021350.1 | AET4Gv20308200.1 |  | Os09t0269000-00 | Zm00001d034360_P001 | AT2G24590.1 | KRH35302 | PGSC0003DMT400061471 |
| TRIDC3BG041600.14 | AET5Gv20055100.1 |  | Os09t0521700-01 | Zm00001d034649_P001 | AT1G23860.3 | KRG89905 | PGSC0003DMT400015113 |
| TRIDC4AG053200.2 | AET7Gv20384800.16 |  | Os10t0545300-01 | Zm00001d034721_P001 | AT1G54490.2 | KRH53199 | PGSC0003DMT400060436 |
| TRIDC5AG013380.3 | AET6Gv20863500.3 |  | Os11t0296500-01 | Zm00001d034768_P001 | AT2G15180.1 | KRH64720 | PGSC0003DMT400060435 |
| TRIDC5AG061480.2 | AET1Gv20003500.2 |  | Os11t0531300-01 | Zm00001d035551_P001 |  | KRH67600 | PGSC0003DMT400079284 |
| TRIDC5AG068360.1 | AET5Gv21002100.1 |  | Os11t0573200-01 | Zm00001d035552_P004 |  | KRH67601 | PGSC0003DMT400079285 |
| TRIDC5BG015470.3 | AET5Gv21150500.4 |  | Os11t0575200-00 | Zm00001d035608_P001 |  | KRH67602 | PGSC0003DMT400076495 |
| TRIDC5BG065570.2 | AET5Gv21152600.1 |  | Os12t0564600-01 | Zm00001d037543_P001 |  | KRH67603 | PGSC0003DMT400076496 |
| TRIDC5BG073610.1 | AET7Gv20667100.1 |  |  | Zm00001d045076_P001 |  | KRG95886 | PGSC0003DMT400076497 |
| TRIDC5BG077060.1 | AET2Gv21252200.1 |  |  | Zm00001d047389_P001 |  | KRG95887 | PGSC0003DMT400087384 |
| TRIDC6AG008520.2 | AET7Gv21152700.1 |  |  | Zm00001d051052_P001 |  | KRG95888 | PGSC0003DMT400043579 |
| TRIDC6AG033900.1 | AET3Gv20711400.3 |  |  | Zm00001d052062_P002 |  | KRH67604 | PGSC0003DMT400043581 |
| TRIDC6AG052490.1 | AET2Gv20518500.4 |  |  | Zm00001d052668_P001 |  | KRH67596 | PGSC0003DMT400080556 |
| TRIDC6BG012830.2 | AET5Gv21129800.4 |  |  | Zm00001d052941_P001 |  | KRH67597 | PGSC0003DMT400043580 |
| TRIDC6BG040440.1 | AET1Gv20246600.1 |  |  | Zm00001d053908_P001 |  | KRH67598 | PGSC0003DMT400059695 |
| TRIDC6BG061250.6 | AET7Gv20780300.1 |  |  |  |  | KRH67599 | PGSC0003DMT400045627 |
| TRIDC7AG018740.1 | AET1Gv20442000.1 |  |  |  |  | KRG95883 | PGSC0003DMT400045628 |
| TRIDC7AG045770.1 | AET6Gv20581200.3 |  |  |  |  | KRG95884 | PGSC0003DMT400045629 |
| TRIDC7BG008240.1 | AET4Gv20455500.1 |  |  |  |  | KRG95885 | PGSC0003DMT400093996 |
| TRIDC7BG037700.2 | AET4Gv20420500.2 |  |  |  |  | KRG95881 | PGSC0003DMT400088114 |
|  | AET2Gv21036600.1 |  |  |  |  | KRG95882 | PGSC0003DMT400079815 |
|  | AET3Gv20987700.3 |  |  |  |  | KRH67595 | PGSC0003DMT400085039 |
|  | AET7Gv21297800.1 |  |  |  |  | KRG95880 | PGSC0003DMT400075974 |
|  | AET2Gv20517300.1 |  |  |  |  | KRG95879 | PGSC0003DMT400075975 |
|  | AET1Gv20786700.2 |  |  |  |  | KRH39675 | PGSC0003DMT400075976 |
|  | AET4Gv20781900.1 |  |  |  |  | KRG95878 | PGSC0003DMT400075977 |
|  | AET5Gv20237600.14 |  |  |  |  | KRG95876 | PGSC0003DMT400075978 |
|  | AET7Gv21338500.1 |  |  |  |  | KRG95877 | PGSC0003DMT400075979 |
|  |  |  |  |  |  | KRH67593 | PGSC0003DMT400091943 |
|  |  |  |  |  |  | KRH35986 | PGSC0003DMT400067677 |
|  |  |  |  |  |  | KRG90774 | PGSC0003DMT400027781 |
|  |  |  |  |  |  | KRH24282 | PGSC0003DMT400087661 |
|  |  |  |  |  |  | KRH24279 | PGSC0003DMT400092152 |
|  |  |  |  |  |  | KRH24275 | PGSC0003DMT400091243 |
|  |  |  |  |  |  | KRH24272 | PGSC0003DMT400094334 |
|  |  |  |  |  |  | KRH24280 | PGSC0003DMT400084770 |
|  |  |  |  |  |  | KRH24270 | PGSC0003DMT400091705 |
|  |  |  |  |  |  | KRH24283 | PGSC0003DMT400004021 |
|  |  |  |  |  |  | KRH42209 | PGSC0003DMT400088215 |
|  |  |  |  |  |  | KRH24269 | PGSC0003DMT400092287 |
|  |  |  |  |  |  | KRH24266 | PGSC0003DMT400084867 |
|  |  |  |  |  |  | KRH24265 | PGSC0003DMT400088278 |
|  |  |  |  |  |  | KRH24268 | PGSC0003DMT400015115 |
|  |  |  |  |  |  | KRH24267 | PGSC0003DMT400094973 |
|  |  |  |  |  |  | KRH43451 | PGSC0003DMT400093864 |
|  |  |  |  |  |  | KRH24271 | PGSC0003DMT400093884 |
|  |  |  |  |  |  | KRH24264 | PGSC0003DMT400088180 |
|  |  |  |  |  |  | KRH24274 | PGSC0003DMT400079780 |
|  |  |  |  |  |  | KRH24273 | PGSC0003DMT400079781 |
|  |  |  |  |  |  | KRH24278 |  |
|  |  |  |  |  |  | KRG90775 |  |
|  |  |  |  |  |  | KRG90776 |  |
|  |  |  |  |  |  | KRH35987 |  |
|  |  |  |  |  |  | KRH29133 |  |
|  |  |  |  |  |  | KRH16161 |  |
|  |  |  |  |  |  | KRH16159 |  |
|  |  |  |  |  |  | KRH16162 |  |
|  |  |  |  |  |  | KRH16160 |  |
|  |  |  |  |  |  | KRH04932 |  |
|  |  |  |  |  |  | KRH04931 |  |
|  |  |  |  |  |  | KRH24168 |  |
|  |  |  |  |  |  | KRH58335 |  |
|  |  |  |  |  |  | KRG99323 |  |
|  |  |  |  |  |  | KRG99319 |  |
|  |  |  |  |  |  | KRG99320 |  |
|  |  |  |  |  |  | KRG99321 |  |
|  |  |  |  |  |  | KRG99322 |  |
|  |  |  |  |  |  | KRH45694 |  |
|  |  |  |  |  |  | KRH45695 |  |
|  |  |  |  |  |  | KRH45696 |  |
|  |  |  |  |  |  | KRH45697 |  |
|  |  |  |  |  |  | KRH68372 |  |
|  |  |  |  |  |  | KRH45698 |  |
|  |  |  |  |  |  | KRH26235 |  |
|  |  |  |  |  |  | KRG90413 |  |
|  |  |  |  |  |  | KRG96641 |  |
|  |  |  |  |  |  | KRH37434 |  |

**Supplementary Table 2.** List of 50 CCHC-ZFPs in *T. aestivum* and their physicochemical properties.

| **Gene Name** | **Accession Number** | **Protein length (aa)** | **MW (Da)** | **pI** | **Instability index** | **AI** | **GRAVY** | **Subcellular localization** |
| --- | --- | --- | --- | --- | --- | --- | --- | --- |
| TaCCHC1 | TraesCS1A02G032700.1 | 1027 | 109656.92 | 7.97 | 38.94 | 62.15 | -0.525 | Nucleus. |
| TaCCHC2 | TraesCS1A02G040400.1 | 721 | 78355.95 | 8.73 | 60.94 | 56.42 | -0.936 | Chloroplast. |
| TaCCHC3 | TraesCS1A02G049700.2 | 340 | 37656.35 | 10.76 | 93.64 | 37.24 | -1.304 | Chloroplast. Nucleus. |
| TaCCHC4 | TraesCS1A02G085000.1 | 880 | 94257.5 | 7.09 | 41.2 | 67.2 | -0.42 | Nucleus. |
| TaCCHC5 | TraesCS1A02G104500.1 | 864 | 92130.22 | 8.38 | 36.91 | 74.88 | -0.235 | Nucleus. |
| TaCCHC6 | TraesCS1A02G108000.1 | 273 | 31720.58 | 9.43 | 55.05 | 36.45 | -1.501 | Chloroplast. |
| TaCCHC7 | TraesCS1A02G262400.1 | 179 | 17219.28 | 5.55 | 57.2 | 29.39 | -0.748 | Nucleus. |
| TaCCHC8 | TraesCS1A02G262500.1 | 206 | 19194.26 | 5.62 | 58.77 | 27.91 | -0.691 | Nucleus. |
| TaCCHC9 | TraesCS1B02G040800.1 | 1030 | 109947.36 | 8.27 | 37.99 | 61.59 | -0.519 | Nucleus. |
| TaCCHC10 | TraesCS1B02G053400.1 | 741 | 80361.15 | 8.69 | 58.66 | 57.03 | -0.911 | Chloroplast. Nucleus. |
| TaCCHC11 | TraesCS1B02G065900.1 | 339 | 37585.27 | 10.5 | 93.54 | 37.64 | -1.313 | Chloroplast. Nucleus. |
| TaCCHC12 | TraesCS1B02G102200.1 | 849 | 91036.64 | 7.92 | 41.65 | 65.63 | -0.466 | Nucleus. |
| TaCCHC13 | TraesCS1B02G130200.4 | 237 | 26711.36 | 9.57 | 73.66 | 38.27 | -1.24 | Chloroplast. Nucleus. |
| TaCCHC14 | TraesCS1B02G273000.1 | 162 | 15970.11 | 5.55 | 57.79 | 34.88 | -0.752 | Nucleus. |
| TaCCHC15 | TraesCS1D02G041400.1 | 735 | 80059.77 | 8.48 | 62.8 | 56.95 | -0.945 | Chloroplast. Nucleus. |
| TaCCHC16 | TraesCS1D02G051900.1 | 342 | 38488.96 | 10.78 | 96.56 | 30.53 | -1.539 | Chloroplast. Nucleus. |
| TaCCHC17 | TraesCS1D02G086400.1 | 880 | 94509.74 | 6.74 | 44 | 67.98 | -0.434 | Nucleus. |
| TaCCHC18 | TraesCS1D02G096600.1 | 524 | 56917.23 | 5.68 | 35.22 | 72.81 | -0.269 | Nucleus. |
| TaCCHC19 | TraesCS1D02G111000.3 | 221 | 24821.32 | 9.82 | 73.02 | 37.56 | -1.337 | Nucleus. |
| TaCCHC20 | TraesCS1D02G262400.1 | 205 | 19485.7 | 5.52 | 61.16 | 28.54 | -0.715 | Nucleus. |
| TaCCHC21 | TraesCS1D02G262500.1 | 196 | 18430.71 | 5.61 | 56.2 | 29.34 | -0.68 | Nucleus. |
| TaCCHC22 | TraesCS2A02G237400.1 | 293 | 31942.39 | 9.13 | 25.67 | 32.35 | -1.128 | Chloroplast. Nucleus. |
| TaCCHC23 | TraesCS2B02G259400.1 | 293 | 31899.3 | 9.13 | 28.29 | 30.03 | -1.143 | Chloroplast. Nucleus. |
| TaCCHC24 | TraesCS2D02G238900.1 | 293 | 31901.38 | 9.13 | 26.45 | 34.03 | -1.115 | Chloroplast. Nucleus. |
| TaCCHC25 | TraesCS3A02G308000.2 | 317 | 35461.77 | 11.47 | 109.06 | 20.06 | -1.711 | Chloroplast. Nucleus. |
| TaCCHC26 | TraesCS3B02G151100.2 | 307 | 34294.52 | 11.39 | 103.83 | 21.34 | -1.671 | Chloroplast. Nucleus. |
| TaCCHC27 | TraesCS3D02G134500.2 | 308 | 34318.63 | 11.4 | 99.97 | 23.18 | -1.631 | Chloroplast. Nucleus. |
| TaCCHC28 | TraesCS4A02G355700.1 | 211 | 22932.48 | 5.67 | 35.34 | 25.45 | -1.361 | Chloroplast. Nucleus. |
| TaCCHC29 | TraesCS4B02G026400.2 | 285 | 30761.05 | 9.18 | 35.84 | 27.44 | -1.273 | Chloroplast. Nucleus. |
| TaCCHC30 | TraesCS4D02G023800.1 | 284 | 30722.03 | 9.18 | 34.55 | 26.16 | -1.281 | Chloroplast. Nucleus. |
| TaCCHC31 | TraesCS5A02G087300.1 | 1149 | 127804.53 | 7.55 | 39.75 | 74.47 | -0.694 | Nucleus. |
| TaCCHC32 | TraesCS5A02G424900.1 | 370 | 37517.45 | 8.52 | 53.34 | 48.41 | -0.425 | Nucleus. |
| TaCCHC33 | TraesCS5B02G426900.1 | 376 | 37990.89 | 8.52 | 58.65 | 46.84 | -0.451 | Nucleus. |
| TaCCHC34 | TraesCS5B02G516400.1 | 219 | 24106.62 | 6.04 | 32.77 | 24.11 | -1.49 | Chloroplast. Nucleus. |
| TaCCHC35 | TraesCS5D02G098900.3 | 1145 | 127452.8 | 6.61 | 39.7 | 73.63 | -0.725 | Nucleus. |
| TaCCHC36 | TraesCS5D02G516500.1 | 222 | 24273.79 | 6.04 | 34.83 | 23.33 | -1.489 | Chloroplast. Nucleus. |
| TaCCHC37 | TraesCS6A02G069500.1 | 185 | 17664.08 | 6.28 | 49.16 | 31.68 | -0.499 | Nucleus. |
| TaCCHC38 | TraesCS6A02G219000.1 | 235 | 26412.48 | 11.46 | 109.72 | 37.87 | -1.284 | Chloroplast. Nucleus. |
| TaCCHC39 | TraesCS6A02G349500.1 | 194 | 21505.75 | 11.21 | 96.11 | 41.29 | -1.224 | Chloroplast. Nucleus. |
| TaCCHC40 | TraesCS6B02G093900.1 | 195 | 18229.23 | 5.31 | 59.99 | 27.49 | -0.613 | Nucleus. |
| TaCCHC41 | TraesCS6B02G248500.1 | 249 | 27998.29 | 11.63 | 117.51 | 39.28 | -1.294 | Chloroplast. Nucleus. |
| TaCCHC42 | TraesCS6B02G382600.1 | 194 | 21521.75 | 11.21 | 96.55 | 40.77 | -1.237 | Chloroplast. Nucleus. |
| TaCCHC43 | TraesCS6D02G067500.1 | 222 | 21148.67 | 5.94 | 52.25 | 27.7 | -0.548 | Nucleus. |
| TaCCHC44 | TraesCS6D02G202100.1 | 269 | 30221.76 | 11.53 | 114.75 | 38.92 | -1.188 | Chloroplast. Nucleus. |
| TaCCHC45 | TraesCS6D02G332000.1 | 194 | 21519.77 | 11.28 | 96.14 | 41.29 | -1.205 | Chloroplast. Nucleus. |
| TaCCHC46 | TraesCS7A02G152800.1 | 182 | 20558.81 | 11.48 | 109.03 | 44.45 | -1.139 | Chloroplast. Nucleus. |
| TaCCHC47 | TraesCS7A02G326800.1 | 237 | 25917.42 | 7.59 | 32.09 | 23.04 | -1.443 | Chloroplast. Nucleus. |
| TaCCHC48 | TraesCS7B02G056600.1 | 182 | 20585.84 | 11.48 | 107.29 | 44.45 | -1.154 | Chloroplast. Nucleus. |
| TaCCHC49 | TraesCS7B02G227500.1 | 205 | 22010.44 | 6.62 | 37.01 | 26.15 | -1.282 | Chloroplast. Nucleus. |
| TaCCHC50 | TraesCS7D02G323600.1 | 230 | 25053.54 | 7.59 | 35.77 | 23.3 | -1.416 | Chloroplast. Nucleus. |

Note: MW: molecular weight; AI: aliphatic index; pI: isoelectric point; GRAVY: grand average of hydropathicity

**Supplementary Table 3.** Prediction analysis of protein secondary structure of TaCCHC-ZFPs.

| **Name** | **Alpha helix** | **Extended strand** | **Beta turn** | **Random coil** |
| --- | --- | --- | --- | --- |
| TaCCHC1 | 13.73% | 17.43% | 5.74% | 63.10% |
| TaCCHC2 | 30.37% | 12.21% | 3.88% | 53.54% |
| TaCCHC3 | 10.29% | 12.94% | 8.53% | 68.24% |
| TaCCHC4 | 15.80% | 20.00% | 5.80% | 58.41% |
| TaCCHC5 | 16.78% | 19.10% | 6.02% | 58.10% |
| TaCCHC6 | 15.02% | 9.52% | 6.96% | 68.50% |
| TaCCHC7 | 1.68% | 22.91% | 15.64% | 59.78% |
| TaCCHC8 | 3.88% | 25.24% | 21.36% | 49.51% |
| TaCCHC9 | 14.47% | 17.77% | 5.83% | 61.94% |
| TaCCHC10 | 31.71% | 11.74% | 3.78% | 52.77% |
| TaCCHC11 | 10.91% | 12.98% | 7.67% | 68.44% |
| TaCCHC12 | 18.26% | 18.96% | 5.77% | 57.01% |
| TaCCHC13 | 23.03% | 14.59% | 5.76% | 56.62% |
| TaCCHC14 | 0.62% | 22.84% | 11.11% | 65.43% |
| TaCCHC15 | 30.61% | 10.34% | 3.67% | 55.37% |
| TaCCHC16 | 6.43% | 12.57% | 7.60% | 73.39% |
| TaCCHC17 | 16.02% | 20.11% | 6.25% | 57.61% |
| TaCCHC18 | 20.42% | 19.27% | 5.15% | 55.15% |
| TaCCHC19 | 2.26% | 12.67% | 5.43% | 79.64% |
| TaCCHC20 | 1.95% | 21.95% | 15.12% | 60.98% |
| TaCCHC21 | 4.08% | 26.53% | 17.86% | 51.53% |
| TaCCHC22 | 13.99% | 9.90% | 9.22% | 66.89% |
| TaCCHC23 | 14.33% | 8.53% | 6.83% | 70.31% |
| TaCCHC24 | 10.92% | 7.85% | 4.78% | 76.45% |
| TaCCHC25 | 4.42% | 5.99% | 5.99% | 83.60% |
| TaCCHC26 | 5.21% | 7.49% | 7.49% | 79.80% |
| TaCCHC27 | 5.84% | 7.47% | 7.14% | 79.55% |
| TaCCHC28 | 15.17% | 17.54% | 10.43% | 56.87% |
| TaCCHC29 | 13.33% | 8.42% | 11.23% | 67.02% |
| TaCCHC30 | 14.08% | 11.97% | 12.32% | 61.62% |
| TaCCHC31 | 39.25% | 10.79% | 3.05% | 46.91% |
| TaCCHC32 | 10.54% | 13.51% | 5.68% | 70.27% |
| TaCCHC33 | 11.17% | 13.03% | 5.59% | 70.21% |
| TaCCHC34 | 14.16% | 14.16% | 13.24% | 58.45% |
| TaCCHC35 | 40.61% | 11.79% | 4.28% | 43.32% |
| TaCCHC36 | 17.12% | 13.96% | 12.61% | 56.31% |
| TaCCHC37 | 8.11% | 24.32% | 15.14% | 52.43% |
| TaCCHC38 | 12.77% | 12.77% | 6.38% | 68.09% |
| TaCCHC39 | 14.95% | 15.46% | 7.73% | 61.86% |
| TaCCHC40 | 4.10% | 19.49% | 24.62% | 51.79% |
| TaCCHC41 | 13.25% | 11.24% | 6.43% | 69.08% |
| TaCCHC42 | 13.40% | 14.43% | 6.70% | 65.46% |
| TaCCHC43 | 6.76% | 25.68% | 17.57% | 50.00% |
| TaCCHC44 | 18.22% | 8.92% | 7.43% | 65.43% |
| TaCCHC45 | 15.46% | 13.92% | 7.22% | 63.40% |
| TaCCHC46 | 15.93% | 15.38% | 9.89% | 58.79% |
| TaCCHC47 | 15.19% | 10.55% | 10.55% | 63.71% |
| TaCCHC48 | 14.84% | 15.93% | 8.79% | 60.44% |
| TaCCHC49 | 17.07% | 11.71% | 10.24% | 60.98% |
| TaCCHC50 | 16.96% | 10.87% | 10.87% | 61.30% |

Supplementary Table 4. Annotations of TaCCHC-ZFP sequence motifs.

| **Name** | **Sequence** | **Description of Pfam** |
| --- | --- | --- |
| motif 1 | GGGGTCYKCGEPGHFSRDCPN | CCHC |
| motif 2 | RSYSRSPPPRR | NO |
| motif 3 | FVTFDDPRDAEEAIRDLDGKD | RRM |
| motif 4 | MARVYVGNLSWRTTERDLEDAFRVFGKVT | RRM |
| motif 5 | VKGTVKWFNVTKGFGFISPDDGSEDLFVHQSAIKADGYRSL | CSD |
| motif 6 | ETRKRVLQJKDMSGGSVEKPDWGTVCGAE | REPA OB |
| motif 7 | CNKKVTNNGDGMWLCEKCEQSSZTCEYRYLLSCQIQDHTGTTHATAFQEA | Rep Fac-A C |
| motif 8 | SSSLLKINPDFPEAEKLRQWYITEGKLAACTSLSGEISSMG | NO |
| motif 9 | KKAPEVIADENKANTDSASTA | NO |
| motif 10 | VDQFYDLIEVDKVYLISRGSLKPANKRFNPLNNDYEINLDPSTSIEVCS | NO |

***Supplementary Table 5.*** *The Ka/Ks ratios and the date of duplication for duplicate CCHC-ZF genes in T. aestivum.*

| **TaCCHC gene 1** | **TaCCHC gene 2** | **Ka** | **Ks** | **Ka/Ks** | **Duplication time/(Mya)** | **Selective pressure** | **Duplicate type** |
| --- | --- | --- | --- | --- | --- | --- | --- |
| TaCCHC7 | TaCCHC14 | 0.026590673 | 0.133473189 | 0.19922108 | 10.267 | Purifying selection | Segmental |
| TaCCHC1 | TaCCHC9 | 0.016852214 | 0.064829067 | 0.259948428 | 4.987 | Purifying selection | Segmental |
| TaCCHC2 | TaCCHC10 | 0.029324854 | 0.098583918 | 0.297460833 | 7.583 | Purifying selection | Segmental |
| TaCCHC3 | TaCCHC11 | 0.014467788 | 0.093872357 | 0.154121916 | 7.221 | Purifying selection | Segmental |
| TaCCHC4 | TaCCHC12 | 0.015532458 | 0.057091211 | 0.272063904 | 4.392 | Purifying selection | Segmental |
| TaCCHC6 | TaCCHC13 | 0.06027138 | 0.104357683 | 0.577546168 | 8.028 | Purifying selection | Segmental |
| TaCCHC7 | TaCCHC20 | 0.028439426 | 0.194898309 | 0.145919307 | 14.992 | Purifying selection | Segmental |
| TaCCHC3 | TaCCHC16 | 0.070892096 | 0.210945717 | 0.336067955 | 16.227 | Purifying selection | Segmental |
| TaCCHC4 | TaCCHC17 | 0.012029328 | 0.035827707 | 0.335754897 | 2.756 | Purifying selection | Segmental |
| TaCCHC6 | TaCCHC19 | 0.010377198 | 0.047906818 | 0.216612126 | 3.685 | Purifying selection | Segmental |
| TaCCHC14 | TaCCHC20 | 0.019700945 | 0.104516641 | 0.188495774 | 8.040 | Purifying selection | Segmental |
| TaCCHC10 | TaCCHC15 | 0.028032196 | 0.079237432 | 0.353774666 | 6.095 | Purifying selection | Segmental |
| TaCCHC11 | TaCCHC16 | 0.069514266 | 0.21297072 | 0.326402925 | 16.382 | Purifying selection | Segmental |
| TaCCHC12 | TaCCHC17 | 0.017630502 | 0.055400081 | 0.318239636 | 4.262 | Purifying selection | Segmental |
| TaCCHC22 | TaCCHC23 | 0.014088596 | 0.012922071 | 1.090273808 | 0.994 | Purifying selection | Segmental |
| TaCCHC22 | TaCCHC24 | 0.014099632 | 0.017242139 | 0.817742638 | 1.326 | Positive selection | Segmental |
| TaCCHC23 | TaCCHC24 | 0.015678648 | 0.021630915 | 0.724825906 | 1.664 | Purifying selection | Segmental |
| TaCCHC25 | TaCCHC27 | 0.008577649 | 0.067298923 | 0.12745596 | 5.177 | Purifying selection | Segmental |
| TaCCHC28 | TaCCHC34 | 0.010615888 | 0.083034018 | 0.127849871 | 6.387 | Purifying selection | Segmental |
| TaCCHC28 | TaCCHC36 | 0.004212755 | 0.082321269 | 0.051174559 | 6.332 | Purifying selection | Segmental |
| TaCCHC29 | TaCCHC30 | 0.003147133 | 0.083487576 | 0.037695823 | 6.422 | Purifying selection | Segmental |
| TaCCHC32 | TaCCHC33 | 0.012075125 | 0.052686233 | 0.22918938 | 4.053 | Purifying selection | Segmental |
| TaCCHC34 | TaCCHC36 | 0.004036337 | 0.045029599 | 0.089637411 | 3.464 | Purifying selection | Segmental |
| TaCCHC39 | TaCCHC42 | 0.002313924 | 0.100268267 | 0.023077331 | 7.713 | Purifying selection | Segmental |
| TaCCHC37 | TaCCHC40 | 0.048089228 | 0.247720132 | 0.194127248 | 19.055 | Purifying selection | Segmental |
| TaCCHC38 | TaCCHC41 | 0.009615516 | 0.087477678 | 0.109919657 | 6.729 | Purifying selection | Segmental |
| TaCCHC39 | TaCCHC45 | 0.006957916 | 0.092865195 | 0.074924908 | 7.143 | Purifying selection | Segmental |
| TaCCHC38 | TaCCHC44 | 0.005752665 | 0.075280111 | 0.076416795 | 5.791 | Purifying selection | Segmental |
| TaCCHC37 | TaCCHC43 | 0.02620899 | 0.143850226 | 0.182196375 | 11.065 | Purifying selection | Segmental |
| TaCCHC42 | TaCCHC45 | 0.004631431 | 0.034370275 | 0.134751065 | 2.644 | Purifying selection | Segmental |
| TaCCHC41 | TaCCHC44 | 0.007324948 | 0.05951747 | 0.123072228 | 4.578 | Purifying selection | Segmental |
| TaCCHC40 | TaCCHC43 | 0.02741664 | 0.132963649 | 0.206196509 | 10.228 | Purifying selection | Segmental |
| TaCCHC46 | TaCCHC48 | 0.002451984 | 0.044957277 | 0.054540318 | 3.458 | Purifying selection | Segmental |
| TaCCHC47 | TaCCHC49 | 0.004325895 | 0.069173418 | 0.062536959 | 5.321 | Purifying selection | Segmental |
| TaCCHC47 | TaCCHC50 | 0.003830814 | 0.05607177 | 0.068319841 | 4.313 | Purifying selection | Segmental |
| TaCCHC49 | TaCCHC50 | 0 | 0.047879502 | 0 | 3.683 | Purifying selection | Segmental |

Note: Ka: Non-synonymous; Ks: Synonymous

**Supplementary Table 6. The Ka/Ks ratios and the date of duplication for duplicate *CCHC-ZF* genes among *T. aestivum* and other species.**

|  | **gene 1** | **gene 2** | **Ka** | **Ks** | **Ka/Ks** | **Duplication time/(Mya)** | **Selective pressure** |
| --- | --- | --- | --- | --- | --- | --- | --- |
| *T. aestivum & Ae. tauschii* | TaCCHC1 | AET1Gv20067600.13 | 0.009556272 | 0.049191237 | 0.194267764 | 3.783941302 | purifying selection |
|  | TaCCHC5 | AET1Gv20235700.1 | 0.049316529 | 0.095508892 | 0.516355373 | 7.34683781 | purifying selection |
|  | TaCCHC7 | AET1Gv20635200.11 | 0.065564343 | 0.388758342 | 0.16865064 | 29.90448786 | purifying selection |
|  | TaCCHC2 | AET1Gv20088100.10 | 0.011069177 | 0.065548345 | 0.168870428 | 5.042180365 | purifying selection |
|  | TaCCHC12 | AET1Gv20209300.3 | 0.01326979 | 0.071513086 | 0.185557501 | 5.501006626 | purifying selection |
|  | TaCCHC10 | AET1Gv20088100.10 | 0.011026232 | 0.04447244 | 0.247934052 | 3.420956931 | purifying selection |
|  | TaCCHC9 | AET1Gv20067600.13 | 0.015649888 | 0.056062348 | 0.279151496 | 4.312488335 | purifying selection |
|  | TaCCHC14 | AET1Gv20635200.11 | 0.183345614 | 0.420993196 | 0.43550731 | 32.38409197 | purifying selection |
|  | TaCCHC17 | AET1Gv20209300.3 | 0 | 0.002439027 | 0 | 0.187617426 | purifying selection |
|  | TaCCHC15 | AET1Gv20088100.10 | 0 | 0.003919016 | 0 | 0.301462777 | purifying selection |
|  | TaCCHC20 | AET1Gv20635200.11 | 0.031159032 | 0.032972343 | 0.945005103 | 2.536334044 | purifying selection |
|  | TaCCHC24 | 0 | 0 | 0 | NaN | 0 | purifying selection |
|  | TaCCHC28 | AET5Gv21150500.4 | 0.007900007 | 0.079544679 | 0.099315345 | 6.118821459 | purifying selection |
|  | TaCCHC32 | AET5Gv20990500.2 | 0.012075125 | 0.048797695 | 0.247452774 | 3.753668847 | purifying selection |
|  | TaCCHC31 | AET5Gv20237600.14 | 0.013861931 | 0.038490979 | 0.360134533 | 2.960844531 | purifying selection |
|  | TaCCHC33 | AET5Gv20990500.2 | 0.004737481 | 0.029024178 | 0.163225337 | 2.23262907 | purifying selection |
|  | TaCCHC34 | AET5Gv21150500.4 | 0.014599001 | 0.076014371 | 0.192055804 | 5.847259284 | purifying selection |
|  | TaCCHC36 | AET5Gv21150500.4 | 0.005257415 | 0.034316207 | 0.153205007 | 2.63970824 | purifying selection |
|  | TaCCHC39 | AET6Gv20863500.3 | 0.008113669 | 0.104289191 | 0.077799715 | 8.022245487 | purifying selection |
|  | TaCCHC37 | AET6Gv20173700.1 | 0.021128158 | 0.135678092 | 0.155722692 | 10.43677628 | purifying selection |
|  | TaCCHC40 | AET6Gv20173700.1 | 0.032263039 | 0.1412099 | 0.228475757 | 10.86229998 | purifying selection |
|  | TaCCHC42 | AET6Gv20863500.3 | 0 | 0.076826169 | 0 | 5.909705301 | purifying selection |
|  | TaCCHC41 | AET6Gv20581200.3 | 0.04642936 | 0.135223168 | 0.343353591 | 10.40178215 | purifying selection |
|  | TaCCHC43 | AET6Gv20173700.1 | 0.004051327 | 0.005870871 | 0.690072428 | 0.451605497 | purifying selection |
|  | TaCCHC45 | AET6Gv20863500.3 | 0 | 0.050333348 | 0 | 3.871796022 | purifying selection |
|  | TaCCHC44 | AET6Gv20581200.3 | 0.043426404 | 0.084758285 | 0.51235586 | 6.519868052 | purifying selection |
|  | TaCCHC46 | AET7Gv20384800.16 | 0 | 0.107085786 | 0 | 8.237368172 | purifying selection |
|  | TaCCHC47 | AET7Gv20812500.1 | 0.008202406 | 0.05469393 | 0.149969215 | 4.207225379 | purifying selection |
|  | TaCCHC48 | AET7Gv20384800.16 | 0 | 0.025372398 | 0 | 1.951722953 | purifying selection |
|  | TaCCHC49 | AET7Gv20812500.1 | 0 | 0.034000156 | 0 | 2.615396614 | purifying selection |
|  | TaCCHC50 | AET7Gv20812500.1 | 0 | 0.013363382 | 0 | 1.027952499 | purifying selection |
| *T. aestivum & T. dicoccoides* | TaCCHC7 | TRIDC1AG039170.1 | 0.034549953 | 0.084923525 | 0.406836066 | 6.532578833 | purifying selection |
|  | TaCCHC3 | TRIDC1AG006650.1 | 0.013150221 | 0.013299203 | 0.988797669 | 1.023015637 | purifying selection |
|  | TaCCHC4 | TRIDC1AG012090.1 | 0.008955748 | 0.009067664 | 0.987657711 | 0.697512594 | purifying selection |
|  | TaCCHC1 | TRIDC1AG003450.2 | 0.002240647 | 0.004284706 | 0.522940685 | 0.329592752 | purifying selection |
|  | TaCCHC2 | TRIDC1AG004630.1 | 0 | 0.003931857 | 0 | 0.302450536 | purifying selection |
|  | TaCCHC5 | TRIDC1AG015700.1 | 0.002143528 | 0.00172836 | 1.240209289 | 0.13295079 | positive selection |
|  | TaCCHC6 | TRIDC1AG015200.1 | 0.049977265 | 0.056145928 | 0.890131596 | 4.318917577 | purifying selection |
|  | TaCCHC14 | TRIDC1AG039170.1 | 0.078512667 | 0.22369084 | 0.350987402 | 17.20698768 | purifying selection |
|  | TaCCHC12 | TRIDC1AG012090.1 | 0.022011644 | 0.065986952 | 0.333575708 | 5.075919392 | purifying selection |
|  | TaCCHC10 | TRIDC1AG004630.1 | 0.01477577 | 0.048830802 | 0.302591189 | 3.756215505 | purifying selection |
|  | TaCCHC11 | TRIDC1AG006650.1 | 0.029585988 | 0.119919774 | 0.246714844 | 9.224597968 | purifying selection |
|  | TaCCHC9 | TRIDC1AG003450.2 | 0.017199215 | 0.071678483 | 0.239949483 | 5.513729452 | purifying selection |
|  | TaCCHC13 | TRIDC1AG015200.1 | 0.009613976 | 0.058250288 | 0.165045975 | 4.480791378 | purifying selection |
|  | TaCCHC20 | TRIDC1AG039170.1 | 0.016537188 | 0.205109342 | 0.080626206 | 15.77764167 | purifying selection |
|  | TaCCHC17 | TRIDC1AG012090.1 | 0.022580378 | 0.050415616 | 0.447884603 | 3.878124293 | purifying selection |
|  | TaCCHC15 | TRIDC1AG004630.1 | 0.011069177 | 0.057034596 | 0.194078293 | 4.387276615 | purifying selection |
|  | TaCCHC16 | TRIDC1AG006650.1 | 0.015112742 | 0.114809493 | 0.131633208 | 8.831499445 | purifying selection |
|  | TaCCHC19 | TRIDC1AG015200.1 | 0.008284516 | 0.048016394 | 0.172535164 | 3.693568775 | purifying selection |
|  | TaCCHC25 | TRIDC3AG016490.1 | 0 | 0 | NaN | 0 | purifying selection |
|  | TaCCHC26 | TRIDC3AG016490.1 | 0.001986756 | 0.077170817 | 0.025744915 | 5.936216721 | purifying selection |
|  | TaCCHC27 | TRIDC3AG016490.1 | 0.012000256 | 0.069817817 | 0.171879564 | 5.370601331 | purifying selection |
|  | TaCCHC28 | TRIDC4AG053200.2 | 0.002094608 | 0.006483023 | 0.323091234 | 0.498694041 | purifying selection |
|  | TaCCHC34 | TRIDC4AG053200.2 | 0.00606268 | 0.05840788 | 0.103799014 | 4.492913853 | purifying selection |
|  | TaCCHC36 | TRIDC4AG053200.2 | 0.001992695 | 0.077238611 | 0.025799203 | 5.941431609 | purifying selection |
|  | TaCCHC32 | TRIDC5AG061480.2 | 0.002579538 | 0.007874088 | 0.327598346 | 0.605699083 | purifying selection |
|  | TaCCHC31 | TRIDC5AG013380.3 | 0.018278109 | 0.006853274 | 2.667062163 | 0.527174955 | positive selection |
|  | TaCCHC33 | TRIDC5AG061480.2 | 0.009069426 | 0.056899313 | 0.159394297 | 4.376870237 | purifying selection |
|  | TaCCHC38 | TRIDC6AG033900.1 | 0 | 0.011121613 | 0 | 0.855508654 | purifying selection |
|  | TaCCHC39 | TRIDC6AG052490.1 | 0 | 0.013529115 | 0 | 1.040701188 | purifying selection |
|  | TaCCHC37 | TRIDC6AG008520.2 | 0.020954527 | 0.061628857 | 0.340011604 | 4.740681312 | purifying selection |
|  | TaCCHC42 | TRIDC6AG052490.1 | 0.002313924 | 0.100268267 | 0.023077331 | 7.712943593 | purifying selection |
|  | TaCCHC41 | TRIDC6AG033900.1 | 0.021222391 | 0.127847654 | 0.165997499 | 9.834434959 | purifying selection |
|  | TaCCHC40 | TRIDC6AG008520.2 | 0.014134694 | 0.167154108 | 0.084560853 | 12.85800831 | purifying selection |
|  | TaCCHC44 | TRIDC6AG033900.1 | 0.01749082 | 0.134004652 | 0.130523979 | 10.30805013 | purifying selection |
|  | TaCCHC45 | TRIDC6AG052490.1 | 0.006957916 | 0.092865195 | 0.074924908 | 7.14347654 | purifying selection |
|  | TaCCHC43 | TRIDC6AG008520.2 | 0.02467666 | 0.119750616 | 0.206067086 | 9.211585822 | purifying selection |
|  | TaCCHC46 | TRIDC7AG018740.1 | 0 | 0 | NaN | 0 | purifying selection |
|  | TaCCHC47 | TRIDC7AG045770.1 | 0.001914487 | 0 | NAN | 0 | purifying selection |
|  | TaCCHC48 | TRIDC7AG018740.1 | 0.002451984 | 0.044957277 | 0.054540318 | 3.458252075 | purifying selection |
|  | TaCCHC49 | TRIDC7AG045770.1 | 0.002159829 | 0.069173418 | 0.031223391 | 5.321032166 | purifying selection |
|  | TaCCHC50 | TRIDC7AG045770.1 | 0.001912961 | 0.05607177 | 0.034116301 | 4.313213046 | purifying selection |
|  | TaCCHC7 | TRIDC1BG044550.2 | 0.022118507 | 0.135549746 | 0.163176305 | 10.42690351 | purifying selection |
|  | TaCCHC4 | TRIDC1BG014830.1 | 0.015793254 | 0.064949282 | 0.243162876 | 4.996098593 | purifying selection |
|  | TaCCHC2 | TRIDC1BG006490.1 | 0.014693642 | 0.122974158 | 0.119485607 | 9.459550622 | purifying selection |
|  | TaCCHC6 | TRIDC1BG019080.1 | 0.107187034 | 0.166838453 | 0.642460007 | 12.83372713 | purifying selection |
|  | TaCCHC1 | TRIDC1BG004320.5 | 0.016667353 | 0.06298564 | 0.26462147 | 4.845049211 | purifying selection |
|  | TaCCHC12 | TRIDC1BG014830.1 | 0.002406982 | 0.011776493 | 0.204388715 | 0.905884091 | purifying selection |
|  | TaCCHC14 | TRIDC1BG044550.2 | 0.002988052 | 0.027358656 | 0.109217781 | 2.104512035 | purifying selection |
|  | TaCCHC13 | TRIDC1BG019080.1 | 0.044994969 | 0.053749162 | 0.837128743 | 4.134550958 | purifying selection |
|  | TaCCHC10 | TRIDC1BG006490.1 | 0.012074431 | 0.087228891 | 0.13842238 | 6.70991471 | purifying selection |
|  | TaCCHC9 | TRIDC1BG004320.5 | 0.00349302 | 0.009564423 | 0.365209727 | 0.73572484 | purifying selection |
|  | TaCCHC20 | TRIDC1BG044550.2 | 0.009009117 | 0.065258533 | 0.138052711 | 5.019887134 | purifying selection |
|  | TaCCHC17 | TRIDC1BG014830.1 | 0.017015111 | 0.058694807 | 0.28989125 | 4.514985151 | purifying selection |
|  | TaCCHC15 | TRIDC1BG006490.1 | 0.014698936 | 0.083815005 | 0.17537356 | 6.447308054 | purifying selection |
|  | TaCCHC25 | TRIDC3BG021350.1 | 0.00142315 | 0.072287048 | 0.019687487 | 5.560542131 | purifying selection |
|  | TaCCHC26 | TRIDC3BG021350.1 | 0 | 0.004601241 | 0 | 0.353941648 | purifying selection |
|  | TaCCHC27 | TRIDC3BG021350.1 | 0.010024016 | 0.119061292 | 0.084192061 | 9.158560908 | purifying selection |
|  | TaCCHC28 | TRIDC5BG077060.1 | 0.008480656 | 0.083034018 | 0.102134714 | 6.387232178 | purifying selection |
|  | TaCCHC32 | TRIDC5BG065570.2 | 0.013608901 | 0.056344174 | 0.24153165 | 4.334167263 | purifying selection |
|  | TaCCHC34 | TRIDC5BG077060.1 | 0.0020141 | 0 | NAN | 0 | purifying selection |
|  | TaCCHC33 | TRIDC5BG065570.2 | 0.001328022 | 0.004087204 | 0.324921813 | 0.314400275 | purifying selection |
|  | TaCCHC36 | TRIDC5BG077060.1 | 0.002015453 | 0.045029599 | 0.044758405 | 3.463815304 | purifying selection |
|  | TaCCHC39 | TRIDC6BG061250.6 | 0.002316604 | 0.115334665 | 0.020085931 | 8.871897315 | purifying selection |
|  | TaCCHC37 | TRIDC6BG012830.2 | 0.023458702 | 0.1934742 | 0.121249771 | 14.88263078 | purifying selection |
|  | TaCCHC38 | TRIDC6BG040440.1 | 0.009615516 | 0.087477678 | 0.109919657 | 6.729052138 | purifying selection |
|  | TaCCHC42 | TRIDC6BG061250.6 | 0 | 0.041200289 | 0 | 3.169253009 | purifying selection |
|  | TaCCHC40 | TRIDC6BG012830.2 | 0.002324682 | 0.013086483 | 0.177639959 | 1.0066525 | purifying selection |
|  | TaCCHC41 | TRIDC6BG040440.1 | 0 | 0 | 0 | 0 | purifying selection |
|  | TaCCHC45 | TRIDC6BG061250.6 | 0.0046368 | 0.034252528 | 0.13537103 | 2.634809817 | purifying selection |
|  | TaCCHC43 | TRIDC6BG012830.2 | 0.008135673 | 0.079829885 | 0.101912623 | 6.140760379 | purifying selection |
|  | TaCCHC44 | TRIDC6BG040440.1 | 0.007324948 | 0.05951747 | 0.123072228 | 4.578266889 | purifying selection |
|  | TaCCHC47 | TRIDC7BG037700.2 | 0.009615516 | 0.075694439 | 0.127030684 | 5.822649149 | purifying selection |
|  | TaCCHC46 | TRIDC7BG008240.1 | 0.027016844 | 0.077694008 | 0.347733943 | 5.97646213 | purifying selection |
|  | TaCCHC49 | TRIDC7BG037700.2 | 0.002158275 | 0 | NAN | 0 | purifying selection |
|  | TaCCHC48 | TRIDC7BG008240.1 | 0.02957279 | 0.06168289 | 0.479432638 | 4.744837663 | purifying selection |
|  | TaCCHC50 | TRIDC7BG037700.2 | 0.005749908 | 0.043333351 | 0.132690141 | 3.333334654 | purifying selection |
| *T. aestivum & H. vulgare* | TaCCHC1 | HORVU1Hr1G004910.1 | 0.04608144 | 0.11159579 | 0.412931707 | 8.584291532 | purifying selection |
|  | TaCCHC4 | HORVU1Hr1G018380.1 | 0.040873349 | 0.103863079 | 0.393531074 | 7.989467618 | purifying selection |
|  | TaCCHC7 | HORVU1Hr1G064210.1 | 0.045715999 | 0.219206393 | 0.208552308 | 16.86203024 | purifying selection |
|  | TaCCHC12 | HORVU1Hr1G018380.1 | 0.042802585 | 0.110691729 | 0.386682775 | 8.514748355 | purifying selection |
|  | TaCCHC9 | HORVU1Hr1G004910.1 | 0.04385138 | 0.135148918 | 0.324467115 | 10.39607063 | purifying selection |
|  | TaCCHC19 | HORVU1Hr1G024060.10 | 0.03850246 | 0.155392218 | 0.247775987 | 11.9532475 | purifying selection |
|  | TaCCHC17 | HORVU1Hr1G018380.1 | 0.040845517 | 0.113309421 | 0.360477682 | 8.716109281 | purifying selection |
|  | TaCCHC20 | HORVU1Hr1G064210.1 | 0.020754591 | 0.126764621 | 0.163725423 | 9.75112466 | purifying selection |
|  | TaCCHC21 | HORVU1Hr1G064210.1 | 0.037352117 | 0.300758068 | 0.124193233 | 23.13523602 | purifying selection |
|  | TaCCHC28 | HORVU5Hr1G118010.1 | 0.002093146 | 0.1265413 | 0.016541211 | 9.733946116 | purifying selection |
|  | TaCCHC39 | HORVU6Hr1G083840.1 | 0 | 0 | NaN | 0 | purifying selection |
|  | TaCCHC37 | HORVU6Hr1G012510.1 | 0.022132754 | 0.257288406 | 0.086023129 | 19.79141583 | purifying selection |
|  | TaCCHC42 | HORVU6Hr1G083840.1 | 0.004601241 | 0.068855662 | 0.066824445 | 5.29658938 | purifying selection |
|  | TaCCHC40 | HORVU6Hr1G012510.1 | 0.011718988 | 0.232616196 | 0.050379073 | 17.89355356 | purifying selection |
|  | TaCCHC45 | HORVU6Hr1G083840.1 | 0.009216706 | 0.054818913 | 0.168130037 | 4.216839462 | purifying selection |
|  | TaCCHC46 | HORVU7Hr1G031320.1 | 0.004903982 | 0.101585452 | 0.048274447 | 7.814265511 | purifying selection |
|  | TaCCHC48 | HORVU7Hr1G031320.1 | 0.007371067 | 0.084923525 | 0.086796523 | 6.532578833 | purifying selection |
| *T. aestivum & O. sativa* | TaCCHC1 | Os05t0111000-01 | 0.269941467 | 0.713174535 | 0.378506878 | 54.85957961 | purifying selection |
|  | TaCCHC3 | Os05t0120100-01 | 0.117279775 | 0.692902971 | 0.169258583 | 53.3002285 | purifying selection |
|  | TaCCHC13 | Os05t0162600-01 | 0.397073109 | 0.706729478 | 0.561845969 | 54.36380602 | purifying selection |
|  | TaCCHC11 | Os05t0120100-01 | 0.117710484 | 0.771585002 | 0.152556729 | 59.35269246 | purifying selection |
|  | TaCCHC19 | Os05t0162600-01 | 0.35082144 | 0.732938574 | 0.478650534 | 56.37989029 | purifying selection |
|  | TaCCHC16 | Os05t0120100-01 | 0.084954178 | 0.720328628 | 0.117938084 | 55.40989443 | purifying selection |
|  | TaCCHC25 | Os01t0155600-01 | 0.06018752 | 0.578299213 | 0.104076779 | 44.48455486 | purifying selection |
|  | TaCCHC27 | Os01t0155600-01 | 0.073257563 | 0.620094308 | 0.118139389 | 47.69956212 | purifying selection |
|  | TaCCHC28 | Os03t0836200-01 | 0.080710211 | 0.57649105 | 0.140002539 | 44.34546537 | purifying selection |
|  | TaCCHC29 | Os03t0681900-01 | 0.102292569 | 0.605250704 | 0.169008591 | 46.55774643 | purifying selection |
|  | TaCCHC30 | Os03t0681900-01 | 0.103117193 | 0.551085521 | 0.187116498 | 42.3911939 | purifying selection |
|  | TaCCHC31 | Os12t0564600-01 | 0.092906916 | 0.474997213 | 0.195594655 | 36.53824712 | purifying selection |
|  | TaCCHC32 | Os03t0786500-01 | 0.153588913 | 0.512593037 | 0.29963129 | 39.43023358 | purifying selection |
|  | TaCCHC33 | Os03t0786500-01 | 0.152100694 | 0.505243039 | 0.301044611 | 38.86484915 | purifying selection |
|  | TaCCHC34 | Os03t0836200-01 | 0.079572466 | 0.527245328 | 0.150921141 | 40.55733291 | purifying selection |
|  | TaCCHC35 | Os12t0564600-01 | 0.099035013 | 0.480380433 | 0.206159548 | 36.952341 | purifying selection |
|  | TaCCHC36 | Os03t0836200-01 | 0.066223809 | 0.578967061 | 0.114382688 | 44.5359278 | purifying selection |
|  | TaCCHC39 | Os02t0789400-01 | 0.060953814 | 0.514806768 | 0.118401345 | 39.60052058 | purifying selection |
|  | TaCCHC38 | Os02t0610600-01 | 0.052516974 | 0.744812365 | 0.070510341 | 57.29325884 | purifying selection |
|  | TaCCHC37 | Os02t0121100-01 | 0.133624623 | 0.517595207 | 0.258164337 | 39.81501592 | purifying selection |
|  | TaCCHC41 | Os02t0610600-01 | 0.051948375 | 0.681394392 | 0.076238336 | 52.41495326 | purifying selection |
|  | TaCCHC42 | Os02t0789400-01 | 0.060953814 | 0.514806768 | 0.118401345 | 39.60052058 | purifying selection |
|  | TaCCHC40 | Os02t0121100-01 | 0.113580044 | 0.434008462 | 0.261700068 | 33.38526627 | purifying selection |
|  | TaCCHC44 | Os02t0610600-01 | 0.050048344 | 0.675298099 | 0.074112964 | 51.94600763 | purifying selection |
|  | TaCCHC45 | Os02t0789400-01 | 0.063605663 | 0.446405887 | 0.142483925 | 34.33891438 | purifying selection |
|  | TaCCHC43 | Os02t0121100-01 | 0.133317105 | 0.498376187 | 0.267502961 | 38.33662976 | purifying selection |
|  | TaCCHC46 | Os06t0187900-01 | 0.056436872 | 0.480282858 | 0.117507571 | 36.94483526 | purifying selection |
|  | TaCCHC48 | Os06t0187900-01 | 0.059131253 | 0.438629238 | 0.134809193 | 33.74071062 | purifying selection |
| *T. aestivum & Z. mays* | TaCCHC38 | Zm00001d051052_P001 | 0.126619362 | 0.692486629 | 0.182847374 | 53.26820222 | purifying selection |
|  | TaCCHC37 | Zm00001d053908_P001 | 0.137243008 | 0.642582453 | 0.213580385 | 49.42941948 | purifying selection |
|  | TaCCHC41 | Zm00001d051052_P001 | 0.128719701 | 0.770919881 | 0.166968973 | 59.30152932 | purifying selection |
|  | TaCCHC44 | Zm00001d051052_P001 | 0.119994427 | 0.730643756 | 0.164231099 | 56.20336581 | purifying selection |
|  | TaCCHC29 | Zm00001d013543_P001 | 0.098957543 | 0.673943366 | 0.146833618 | 51.84179739 | purifying selection |
|  | TaCCHC30 | Zm00001d013543_P001 | 0.098859572 | 0.589465337 | 0.167710577 | 45.34348745 | purifying selection |
|  | TaCCHC38 | Zm00001d017177_P001 | 0.107643977 | 0.688813983 | 0.156274378 | 52.98569099 | purifying selection |
|  | TaCCHC41 | Zm00001d017177_P001 | 0.114796461 | 0.71446869 | 0.160673886 | 54.95912997 | purifying selection |
|  | TaCCHC44 | Zm00001d017177_P001 | 0.109339276 | 0.722660463 | 0.151301035 | 55.58926639 | purifying selection |
|  | TaCCHC6 | Zm00001d037543_P001 | 0.364190438 | 0.701730375 | 0.518989132 | 53.97925962 | purifying selection |
|  | TaCCHC2 | Zm00001d035608_P001 | 0.207258957 | 0.801322914 | 0.258645989 | 61.64022418 | purifying selection |
|  | TaCCHC3 | Zm00001d035551_P001 | 0.116068024 | 0.799039981 | 0.145259345 | 61.46461391 | purifying selection |
|  | TaCCHC15 | Zm00001d035608_P001 | 0.200841276 | 0.828573669 | 0.242393988 | 63.73643609 | purifying selection |
|  | TaCCHC19 | Zm00001d037543_P001 | 0.364879223 | 0.741861528 | 0.491842762 | 57.06627138 | purifying selection |
|  | TaCCHC46 | Zm00001d045076_P001 | 0.213145577 | 1.292262238 | 0.164939879 | 99.40478751 | purifying selection |
|  | TaCCHC48 | Zm00001d045076_P001 | 0.213248989 | 1.247421065 | 0.17095189 | 95.95546656 | purifying selection |

**Supplementary Table 7.** Information for cis-acting elements of *TaCCHC-ZF* genes.

| **Genes** | **Cis-acting elements** | **Starts** | **Ends** | **Types** |
| --- | --- | --- | --- | --- |
| TaCCHC1 | CGTCA-motif | 408 | 413 | MeJA responsive |
| TaCCHC1 | CGTCA-motif | 801 | 806 | MeJA responsive |
| TaCCHC1 | LTR | 614 | 620 | low-temperature responsive |
| TaCCHC1 | LTR | 666 | 672 | low-temperature responsive |
| TaCCHC1 | LTR | 718 | 724 | low-temperature responsive |
| TaCCHC1 | LTR | 1268 | 1274 | low-temperature responsive |
| TaCCHC1 | AuxRR-core | 1335 | 1342 | auxin responsive |
| TaCCHC1 | TGACG-motif | 408 | 413 | MeJA responsive |
| TaCCHC1 | TGACG-motif | 801 | 806 | MeJA responsive |
| TaCCHC1 | ARE | 293 | 299 | anaerobic induction |
| TaCCHC1 | ARE | 783 | 789 | anaerobic induction |
| TaCCHC1 | GC-motif | 606 | 612 | anoxic specific inducibility |
| TaCCHC1 | GC-motif | 658 | 664 | anoxic specific inducibility |
| TaCCHC1 | GC-motif | 710 | 716 | anoxic specific inducibility |
| TaCCHC1 | GC-motif | 1376 | 1382 | anoxic specific inducibility |
| TaCCHC1 | TGA-element | 625 | 631 | auxin responsive |
| TaCCHC1 | TGA-element | 677 | 683 | auxin responsive |
| TaCCHC1 | TGA-element | 729 | 735 | auxin responsive |
| TaCCHC1 | TATA-box | 287 | 293 | conservative cis-element |
| TaCCHC1 | TATA-box | 289 | 293 | conservative cis-element |
| TaCCHC1 | TATA-box | 432 | 439 | conservative cis-element |
| TaCCHC1 | CAAT-box | 31 | 35 | conservative cis-element |
| TaCCHC1 | CAAT-box | 48 | 53 | conservative cis-element |
| TaCCHC1 | CAAT-box | 49 | 53 | conservative cis-element |
| TaCCHC1 | CAAT-box | 193 | 203 | conservative cis-element |
| TaCCHC1 | CAAT-box | 361 | 366 | conservative cis-element |
| TaCCHC1 | CAAT-box | 385 | 390 | conservative cis-element |
| TaCCHC1 | CAAT-box | 398 | 403 | conservative cis-element |
| TaCCHC1 | CAAT-box | 438 | 442 | conservative cis-element |
| TaCCHC1 | CAAT-box | 454 | 458 | conservative cis-element |
| TaCCHC1 | CAAT-box | 632 | 636 | conservative cis-element |
| TaCCHC1 | CAAT-box | 736 | 740 | conservative cis-element |
| TaCCHC1 | CAAT-box | 815 | 819 | conservative cis-element |
| TaCCHC1 | CAAT-box | 1460 | 1465 | conservative cis-element |
| TaCCHC1 | CAAT-box | 1471 | 1476 | conservative cis-element |
| TaCCHC1 | CAAT-box | 1472 | 1476 | conservative cis-element |
| TaCCHC1 | CAAT-box | 1476 | 1481 | conservative cis-element |
| TaCCHC1 | CAAT-box | 1477 | 1481 | conservative cis-element |
| TaCCHC1 | CAAT-box | 1481 | 1486 | conservative cis-element |
| TaCCHC1 | CAAT-box | 1482 | 1486 | conservative cis-element |
| TaCCHC2 | MBS | 227 | 233 | drought responsive |
| TaCCHC2 | GARE-motif | 583 | 590 | gibberellin responsive |
| TaCCHC2 | CGTCA-motif | 1440 | 1445 | MeJA responsive |
| TaCCHC2 | TGACG-motif | 1440 | 1445 | MeJA responsive |
| TaCCHC2 | ABRE | 1054 | 1063 | ABA responsive |
| TaCCHC2 | ABRE | 1383 | 1388 | ABA responsive |
| TaCCHC2 | GC-motif | 1268 | 1274 | anoxic specific inducibility |
| TaCCHC2 | ARE | 908 | 914 | anaerobic induction |
| TaCCHC2 | ARE | 913 | 919 | anaerobic induction |
| TaCCHC2 | ARE | 918 | 924 | anaerobic induction |
| TaCCHC2 | ARE | 923 | 929 | anaerobic induction |
| TaCCHC2 | TATA-box | 122 | 128 | conservative cis-element |
| TaCCHC2 | TATA-box | 124 | 128 | conservative cis-element |
| TaCCHC2 | TATA-box | 530 | 535 | conservative cis-element |
| TaCCHC2 | TATA-box | 531 | 535 | conservative cis-element |
| TaCCHC2 | TATA-box | 710 | 714 | conservative cis-element |
| TaCCHC2 | TATA-box | 738 | 744 | conservative cis-element |
| TaCCHC2 | TATA-box | 739 | 744 | conservative cis-element |
| TaCCHC2 | TATA-box | 740 | 744 | conservative cis-element |
| TaCCHC2 | TATA-box | 1423 | 1427 | conservative cis-element |
| TaCCHC2 | CAAT-box | 108 | 112 | conservative cis-element |
| TaCCHC2 | CAAT-box | 190 | 195 | conservative cis-element |
| TaCCHC2 | CAAT-box | 191 | 195 | conservative cis-element |
| TaCCHC2 | CAAT-box | 209 | 214 | conservative cis-element |
| TaCCHC2 | CAAT-box | 270 | 275 | conservative cis-element |
| TaCCHC2 | CAAT-box | 436 | 441 | conservative cis-element |
| TaCCHC2 | CAAT-box | 635 | 640 | conservative cis-element |
| TaCCHC2 | CAAT-box | 651 | 655 | conservative cis-element |
| TaCCHC2 | CAAT-box | 719 | 723 | conservative cis-element |
| TaCCHC2 | CAAT-box | 734 | 738 | conservative cis-element |
| TaCCHC2 | CAAT-box | 754 | 758 | conservative cis-element |
| TaCCHC2 | CAAT-box | 763 | 767 | conservative cis-element |
| TaCCHC2 | CAAT-box | 765 | 769 | conservative cis-element |
| TaCCHC2 | CAAT-box | 769 | 773 | conservative cis-element |
| TaCCHC2 | CAAT-box | 888 | 895 | conservative cis-element |
| TaCCHC2 | CAAT-box | 963 | 968 | conservative cis-element |
| TaCCHC2 | CAAT-box | 1393 | 1397 | conservative cis-element |
| TaCCHC3 | TC-rich repeats | 1128 | 1137 | defense and stress responsive |
| TaCCHC3 | TC-rich repeats | 1381 | 1390 | defense and stress responsive |
| TaCCHC3 | ARE | 1332 | 1338 | anaerobic induction |
| TaCCHC3 | GC-motif | 596 | 602 | anoxic specific inducibility |
| TaCCHC3 | LTR | 612 | 618 | low-temperature responsive |
| TaCCHC3 | LTR | 627 | 633 | low-temperature responsive |
| TaCCHC3 | MBS | 883 | 889 | drought responsive |
| TaCCHC3 | MBS | 1287 | 1293 | drought responsive |
| TaCCHC3 | CAAT-box | 718 | 723 | conservative cis-element |
| TaCCHC3 | CAAT-box | 787 | 791 | conservative cis-element |
| TaCCHC3 | CAAT-box | 923 | 930 | conservative cis-element |
| TaCCHC3 | CAAT-box | 928 | 933 | conservative cis-element |
| TaCCHC3 | CAAT-box | 957 | 962 | conservative cis-element |
| TaCCHC3 | CAAT-box | 1093 | 1098 | conservative cis-element |
| TaCCHC3 | CAAT-box | 1180 | 1184 | conservative cis-element |
| TaCCHC3 | CAAT-box | 1275 | 1280 | conservative cis-element |
| TaCCHC3 | CAAT-box | 1330 | 1335 | conservative cis-element |
| TaCCHC3 | CAAT-box | 1350 | 1355 | conservative cis-element |
| TaCCHC3 | CAAT-box | 1358 | 1362 | conservative cis-element |
| TaCCHC3 | CAAT-box | 1451 | 1455 | conservative cis-element |
| TaCCHC3 | CAAT-box | 1496 | 1501 | conservative cis-element |
| TaCCHC3 | TATA-box | 836 | 840 | conservative cis-element |
| TaCCHC3 | TATA-box | 870 | 876 | conservative cis-element |
| TaCCHC3 | TATA-box | 872 | 876 | conservative cis-element |
| TaCCHC3 | TATA-box | 935 | 943 | conservative cis-element |
| TaCCHC3 | TATA-box | 1013 | 1020 | conservative cis-element |
| TaCCHC3 | TATA-box | 1014 | 1020 | conservative cis-element |
| TaCCHC3 | TATA-box | 1015 | 1021 | conservative cis-element |
| TaCCHC3 | TATA-box | 1016 | 1020 | conservative cis-element |
| TaCCHC3 | TATA-box | 1045 | 1052 | conservative cis-element |
| TaCCHC3 | TATA-box | 1046 | 1052 | conservative cis-element |
| TaCCHC3 | TATA-box | 1047 | 1052 | conservative cis-element |
| TaCCHC3 | TATA-box | 1048 | 1052 | conservative cis-element |
| TaCCHC3 | TATA-box | 1218 | 1222 | conservative cis-element |
| TaCCHC4 | TGACG-motif | 1141 | 1146 | MeJA responsive |
| TaCCHC4 | AuxRR-core | 1323 | 1330 | auxin responsive |
| TaCCHC4 | LTR | 1291 | 1297 | low-temperature responsive |
| TaCCHC4 | CGTCA-motif | 1141 | 1146 | MeJA responsive |
| TaCCHC4 | GC-motif | 656 | 662 | anoxic specific inducibility |
| TaCCHC4 | GC-motif | 1283 | 1289 | anoxic specific inducibility |
| TaCCHC4 | TATA-box | 308 | 317 | conservative cis-element |
| TaCCHC4 | TATA-box | 310 | 316 | conservative cis-element |
| TaCCHC4 | TATA-box | 311 | 317 | conservative cis-element |
| TaCCHC4 | TATA-box | 312 | 316 | conservative cis-element |
| TaCCHC4 | TATA-box | 1114 | 1118 | conservative cis-element |
| TaCCHC4 | CAAT-box | 74 | 78 | conservative cis-element |
| TaCCHC4 | CAAT-box | 85 | 90 | conservative cis-element |
| TaCCHC4 | CAAT-box | 86 | 90 | conservative cis-element |
| TaCCHC4 | CAAT-box | 232 | 236 | conservative cis-element |
| TaCCHC4 | CAAT-box | 248 | 253 | conservative cis-element |
| TaCCHC4 | CAAT-box | 541 | 545 | conservative cis-element |
| TaCCHC4 | CAAT-box | 807 | 811 | conservative cis-element |
| TaCCHC4 | CAAT-box | 934 | 939 | conservative cis-element |
| TaCCHC4 | CAAT-box | 948 | 953 | conservative cis-element |
| TaCCHC4 | CAAT-box | 957 | 961 | conservative cis-element |
| TaCCHC4 | CAAT-box | 1354 | 1359 | conservative cis-element |
| TaCCHC4 | CAAT-box | 1355 | 1359 | conservative cis-element |
| TaCCHC4 | CAAT-box | 1458 | 1463 | conservative cis-element |
| TaCCHC5 | ERE | 950 | 958 | ethylene responsive |
| TaCCHC5 | ARE | 538 | 544 | anaerobic induction |
| TaCCHC5 | TGACG-motif | 334 | 339 | MeJA responsive |
| TaCCHC5 | TGACG-motif | 345 | 350 | MeJA responsive |
| TaCCHC5 | CGTCA-motif | 334 | 339 | MeJA responsive |
| TaCCHC5 | CGTCA-motif | 345 | 350 | MeJA responsive |
| TaCCHC5 | MBS | 348 | 354 | drought responsive |
| TaCCHC5 | MBS | 787 | 793 | drought responsive |
| TaCCHC5 | GARE-motif | 767 | 774 | gibberellin responsive |
| TaCCHC5 | CAAT-box | 251 | 255 | conservative cis-element |
| TaCCHC5 | CAAT-box | 318 | 323 | conservative cis-element |
| TaCCHC5 | CAAT-box | 332 | 336 | conservative cis-element |
| TaCCHC5 | CAAT-box | 429 | 437 | conservative cis-element |
| TaCCHC5 | CAAT-box | 430 | 435 | conservative cis-element |
| TaCCHC5 | CAAT-box | 431 | 435 | conservative cis-element |
| TaCCHC5 | CAAT-box | 448 | 453 | conservative cis-element |
| TaCCHC5 | CAAT-box | 476 | 481 | conservative cis-element |
| TaCCHC5 | CAAT-box | 535 | 540 | conservative cis-element |
| TaCCHC5 | CAAT-box | 562 | 566 | conservative cis-element |
| TaCCHC5 | CAAT-box | 571 | 576 | conservative cis-element |
| TaCCHC5 | CAAT-box | 635 | 639 | conservative cis-element |
| TaCCHC5 | CAAT-box | 644 | 648 | conservative cis-element |
| TaCCHC5 | CAAT-box | 651 | 656 | conservative cis-element |
| TaCCHC5 | CAAT-box | 652 | 656 | conservative cis-element |
| TaCCHC5 | CAAT-box | 661 | 666 | conservative cis-element |
| TaCCHC5 | CAAT-box | 748 | 752 | conservative cis-element |
| TaCCHC5 | CAAT-box | 818 | 822 | conservative cis-element |
| TaCCHC5 | CAAT-box | 827 | 831 | conservative cis-element |
| TaCCHC5 | CAAT-box | 874 | 879 | conservative cis-element |
| TaCCHC5 | CAAT-box | 920 | 924 | conservative cis-element |
| TaCCHC5 | CAAT-box | 938 | 942 | conservative cis-element |
| TaCCHC5 | CAAT-box | 981 | 986 | conservative cis-element |
| TaCCHC5 | CAAT-box | 1029 | 1034 | conservative cis-element |
| TaCCHC5 | CAAT-box | 1030 | 1034 | conservative cis-element |
| TaCCHC5 | CAAT-box | 1032 | 1036 | conservative cis-element |
| TaCCHC5 | CAAT-box | 1101 | 1105 | conservative cis-element |
| TaCCHC5 | CAAT-box | 1336 | 1341 | conservative cis-element |
| TaCCHC5 | CAAT-box | 1337 | 1341 | conservative cis-element |
| TaCCHC5 | TATA-box | 65 | 69 | conservative cis-element |
| TaCCHC5 | TATA-box | 759 | 763 | conservative cis-element |
| TaCCHC5 | TATA-box | 837 | 841 | conservative cis-element |
| TaCCHC5 | TATA-box | 964 | 971 | conservative cis-element |
| TaCCHC5 | TATA-box | 1011 | 1015 | conservative cis-element |
| TaCCHC5 | TATA-box | 1018 | 1027 | conservative cis-element |
| TaCCHC5 | TATA-box | 1019 | 1026 | conservative cis-element |
| TaCCHC5 | TATA-box | 1020 | 1026 | conservative cis-element |
| TaCCHC5 | TATA-box | 1021 | 1026 | conservative cis-element |
| TaCCHC5 | TATA-box | 1022 | 1026 | conservative cis-element |
| TaCCHC5 | TATA-box | 1052 | 1058 | conservative cis-element |
| TaCCHC5 | TATA-box | 1053 | 1060 | conservative cis-element |
| TaCCHC5 | TATA-box | 1054 | 1060 | conservative cis-element |
| TaCCHC5 | TATA-box | 1056 | 1060 | conservative cis-element |
| TaCCHC5 | TATA-box | 1061 | 1067 | conservative cis-element |
| TaCCHC5 | TATA-box | 1062 | 1067 | conservative cis-element |
| TaCCHC5 | TATA-box | 1063 | 1067 | conservative cis-element |
| TaCCHC5 | TATA-box | 1077 | 1083 | conservative cis-element |
| TaCCHC5 | TATA-box | 1078 | 1084 | conservative cis-element |
| TaCCHC5 | TATA-box | 1079 | 1085 | conservative cis-element |
| TaCCHC5 | TATA-box | 1080 | 1086 | conservative cis-element |
| TaCCHC5 | TATA-box | 1081 | 1087 | conservative cis-element |
| TaCCHC5 | TATA-box | 1082 | 1088 | conservative cis-element |
| TaCCHC5 | TATA-box | 1083 | 1089 | conservative cis-element |
| TaCCHC5 | TATA-box | 1084 | 1090 | conservative cis-element |
| TaCCHC5 | TATA-box | 1085 | 1091 | conservative cis-element |
| TaCCHC5 | TATA-box | 1086 | 1092 | conservative cis-element |
| TaCCHC5 | TATA-box | 1087 | 1093 | conservative cis-element |
| TaCCHC5 | TATA-box | 1088 | 1094 | conservative cis-element |
| TaCCHC5 | TATA-box | 1090 | 1094 | conservative cis-element |
| TaCCHC5 | TATA-box | 1095 | 1101 | conservative cis-element |
| TaCCHC5 | TATA-box | 1096 | 1101 | conservative cis-element |
| TaCCHC5 | TATA-box | 1097 | 1101 | conservative cis-element |
| TaCCHC6 | P-box | 717 | 724 | gibberellin responsive |
| TaCCHC6 | ARE | 811 | 817 | anaerobic induction |
| TaCCHC6 | ABRE | 356 | 365 | ABA responsive |
| TaCCHC6 | ABRE | 1198 | 1203 | ABA responsive |
| TaCCHC6 | ABRE | 1378 | 1383 | ABA responsive |
| TaCCHC6 | GC-motif | 387 | 393 | anoxic specific inducibility |
| TaCCHC6 | GC-motif | 489 | 495 | anoxic specific inducibility |
| TaCCHC6 | TCA-element | 848 | 857 | salicylic acid responsive |
| TaCCHC6 | MBS | 222 | 228 | drought responsive |
| TaCCHC6 | MBS | 1492 | 1498 | drought responsive |
| TaCCHC6 | CAAT-box | 19 | 23 | conservative cis-element |
| TaCCHC6 | CAAT-box | 502 | 506 | conservative cis-element |
| TaCCHC6 | CAAT-box | 708 | 713 | conservative cis-element |
| TaCCHC6 | CAAT-box | 786 | 791 | conservative cis-element |
| TaCCHC6 | CAAT-box | 891 | 896 | conservative cis-element |
| TaCCHC6 | CAAT-box | 901 | 905 | conservative cis-element |
| TaCCHC6 | CAAT-box | 960 | 965 | conservative cis-element |
| TaCCHC6 | CAAT-box | 981 | 985 | conservative cis-element |
| TaCCHC6 | CAAT-box | 1006 | 1010 | conservative cis-element |
| TaCCHC6 | CAAT-box | 1092 | 1097 | conservative cis-element |
| TaCCHC6 | CAAT-box | 1182 | 1186 | conservative cis-element |
| TaCCHC6 | CAAT-box | 1191 | 1195 | conservative cis-element |
| TaCCHC6 | CAAT-box | 1263 | 1268 | conservative cis-element |
| TaCCHC6 | CAAT-box | 1319 | 1323 | conservative cis-element |
| TaCCHC6 | CAAT-box | 1330 | 1334 | conservative cis-element |
| TaCCHC6 | CAAT-box | 1354 | 1358 | conservative cis-element |
| TaCCHC6 | CAAT-box | 1398 | 1402 | conservative cis-element |
| TaCCHC6 | CAAT-box | 1404 | 1409 | conservative cis-element |
| TaCCHC6 | CAAT-box | 1421 | 1426 | conservative cis-element |
| TaCCHC6 | CAAT-box | 1449 | 1453 | conservative cis-element |
| TaCCHC6 | CAAT-box | 1479 | 1483 | conservative cis-element |
| TaCCHC6 | TATA-box | 266 | 272 | conservative cis-element |
| TaCCHC6 | TATA-box | 267 | 271 | conservative cis-element |
| TaCCHC6 | TATA-box | 719 | 728 | conservative cis-element |
| TaCCHC6 | TATA-box | 951 | 955 | conservative cis-element |
| TaCCHC6 | TATA-box | 997 | 1003 | conservative cis-element |
| TaCCHC6 | TATA-box | 999 | 1003 | conservative cis-element |
| TaCCHC6 | TATA-box | 1046 | 1052 | conservative cis-element |
| TaCCHC6 | TATA-box | 1048 | 1052 | conservative cis-element |
| TaCCHC6 | TATA-box | 1126 | 1130 | conservative cis-element |
| TaCCHC6 | TATA-box | 1308 | 1314 | conservative cis-element |
| TaCCHC6 | TATA-box | 1309 | 1315 | conservative cis-element |
| TaCCHC6 | TATA-box | 1311 | 1315 | conservative cis-element |
| TaCCHC6 | TATA-box | 1394 | 1400 | conservative cis-element |
| TaCCHC6 | TATA-box | 1395 | 1399 | conservative cis-element |
| TaCCHC6 | TATA-box | 1410 | 1416 | conservative cis-element |
| TaCCHC6 | TATA-box | 1412 | 1416 | conservative cis-element |
| TaCCHC7 | AuxRR-core | 956 | 963 | auxin responsive |
| TaCCHC7 | TGACG-motif | 48 | 53 | MeJA responsive |
| TaCCHC7 | TGACG-motif | 675 | 680 | MeJA responsive |
| TaCCHC7 | TCA-element | 1284 | 1293 | salicylic acid responsive |
| TaCCHC7 | CGTCA-motif | 48 | 53 | MeJA responsive |
| TaCCHC7 | CGTCA-motif | 675 | 680 | MeJA responsive |
| TaCCHC7 | MBS | 73 | 79 | drought responsive |
| TaCCHC7 | LTR | 1486 | 1492 | low-temperature responsive |
| TaCCHC7 | TC-rich repeats | 1167 | 1176 | defense and stress responsive |
| TaCCHC7 | ARE | 579 | 585 | anaerobic induction |
| TaCCHC7 | ARE | 856 | 862 | anaerobic induction |
| TaCCHC7 | GC-motif | 1371 | 1377 | anoxic specific inducibility |
| TaCCHC7 | ABRE | 691 | 698 | ABA responsive |
| TaCCHC7 | ABRE | 1103 | 1108 | ABA responsive |
| TaCCHC7 | ABRE | 1153 | 1161 | ABA responsive |
| TaCCHC7 | ABRE | 1154 | 1159 | ABA responsive |
| TaCCHC7 | TATA-box | 275 | 279 | conservative cis-element |
| TaCCHC7 | TATA-box | 374 | 380 | conservative cis-element |
| TaCCHC7 | TATA-box | 375 | 381 | conservative cis-element |
| TaCCHC7 | TATA-box | 376 | 382 | conservative cis-element |
| TaCCHC7 | TATA-box | 377 | 383 | conservative cis-element |
| TaCCHC7 | TATA-box | 378 | 382 | conservative cis-element |
| TaCCHC7 | TATA-box | 411 | 416 | conservative cis-element |
| TaCCHC7 | TATA-box | 412 | 416 | conservative cis-element |
| TaCCHC7 | TATA-box | 794 | 800 | conservative cis-element |
| TaCCHC7 | TATA-box | 795 | 801 | conservative cis-element |
| TaCCHC7 | TATA-box | 796 | 802 | conservative cis-element |
| TaCCHC7 | TATA-box | 797 | 803 | conservative cis-element |
| TaCCHC7 | TATA-box | 798 | 804 | conservative cis-element |
| TaCCHC7 | TATA-box | 799 | 803 | conservative cis-element |
| TaCCHC7 | TATA-box | 890 | 897 | conservative cis-element |
| TaCCHC7 | TATA-box | 1202 | 1209 | conservative cis-element |
| TaCCHC7 | TATA-box | 1203 | 1209 | conservative cis-element |
| TaCCHC7 | TATA-box | 1205 | 1209 | conservative cis-element |
| TaCCHC7 | TATA-box | 1246 | 1252 | conservative cis-element |
| TaCCHC7 | TATA-box | 1247 | 1252 | conservative cis-element |
| TaCCHC7 | TATA-box | 1248 | 1252 | conservative cis-element |
| TaCCHC7 | TATA-box | 1386 | 1392 | conservative cis-element |
| TaCCHC7 | TATA-box | 1388 | 1392 | conservative cis-element |
| TaCCHC7 | TATA-box | 1409 | 1417 | conservative cis-element |
| TaCCHC7 | TATA-box | 1410 | 1417 | conservative cis-element |
| TaCCHC7 | TATA-box | 1411 | 1417 | conservative cis-element |
| TaCCHC7 | TATA-box | 1412 | 1417 | conservative cis-element |
| TaCCHC7 | TATA-box | 1413 | 1417 | conservative cis-element |
| TaCCHC7 | CAAT-box | 44 | 48 | conservative cis-element |
| TaCCHC7 | CAAT-box | 46 | 50 | conservative cis-element |
| TaCCHC7 | CAAT-box | 260 | 264 | conservative cis-element |
| TaCCHC7 | CAAT-box | 631 | 635 | conservative cis-element |
| TaCCHC7 | CAAT-box | 656 | 661 | conservative cis-element |
| TaCCHC7 | CAAT-box | 876 | 880 | conservative cis-element |
| TaCCHC7 | CAAT-box | 878 | 882 | conservative cis-element |
| TaCCHC7 | CAAT-box | 899 | 903 | conservative cis-element |
| TaCCHC7 | CAAT-box | 941 | 945 | conservative cis-element |
| TaCCHC7 | CAAT-box | 948 | 952 | conservative cis-element |
| TaCCHC7 | CAAT-box | 971 | 975 | conservative cis-element |
| TaCCHC7 | CAAT-box | 1148 | 1153 | conservative cis-element |
| TaCCHC7 | CAAT-box | 1211 | 1216 | conservative cis-element |
| TaCCHC7 | CAAT-box | 1222 | 1227 | conservative cis-element |
| TaCCHC8 | ARE | 226 | 232 | anaerobic induction |
| TaCCHC8 | ARE | 620 | 626 | anaerobic induction |
| TaCCHC8 | ARE | 840 | 846 | anaerobic induction |
| TaCCHC8 | ARE | 913 | 919 | anaerobic induction |
| TaCCHC8 | ARE | 1005 | 1011 | anaerobic induction |
| TaCCHC8 | ARE | 1222 | 1228 | anaerobic induction |
| TaCCHC8 | TCA-element | 843 | 852 | salicylic acid responsive |
| TaCCHC8 | CGTCA-motif | 566 | 571 | MeJA responsive |
| TaCCHC8 | LTR | 605 | 611 | low-temperature responsive |
| TaCCHC8 | LTR | 636 | 642 | low-temperature responsive |
| TaCCHC8 | LTR | 859 | 865 | low-temperature responsive |
| TaCCHC8 | ABRE | 387 | 396 | ABA responsive |
| TaCCHC8 | ABRE | 1105 | 1114 | ABA responsive |
| TaCCHC8 | ABRE | 1107 | 1113 | ABA responsive |
| TaCCHC8 | ABRE | 1108 | 1113 | ABA responsive |
| TaCCHC8 | ABRE | 1201 | 1206 | ABA responsive |
| TaCCHC8 | TGACG-motif | 566 | 571 | MeJA responsive |
| TaCCHC8 | TATA-box | 154 | 160 | conservative cis-element |
| TaCCHC8 | TATA-box | 155 | 159 | conservative cis-element |
| TaCCHC8 | TATA-box | 164 | 171 | conservative cis-element |
| TaCCHC8 | TATA-box | 165 | 171 | conservative cis-element |
| TaCCHC8 | TATA-box | 166 | 171 | conservative cis-element |
| TaCCHC8 | TATA-box | 167 | 171 | conservative cis-element |
| TaCCHC8 | TATA-box | 197 | 203 | conservative cis-element |
| TaCCHC8 | TATA-box | 198 | 202 | conservative cis-element |
| TaCCHC8 | CAAT-box | 1 | 5 | conservative cis-element |
| TaCCHC8 | CAAT-box | 16 | 21 | conservative cis-element |
| TaCCHC8 | CAAT-box | 19 | 23 | conservative cis-element |
| TaCCHC8 | CAAT-box | 38 | 43 | conservative cis-element |
| TaCCHC8 | CAAT-box | 93 | 97 | conservative cis-element |
| TaCCHC8 | CAAT-box | 151 | 156 | conservative cis-element |
| TaCCHC8 | CAAT-box | 195 | 199 | conservative cis-element |
| TaCCHC8 | CAAT-box | 491 | 496 | conservative cis-element |
| TaCCHC8 | CAAT-box | 513 | 518 | conservative cis-element |
| TaCCHC8 | CAAT-box | 942 | 946 | conservative cis-element |
| TaCCHC8 | CAAT-box | 1242 | 1246 | conservative cis-element |
| TaCCHC9 | TGACG-motif | 158 | 163 | MeJA responsive |
| TaCCHC9 | TGACG-motif | 603 | 608 | MeJA responsive |
| TaCCHC9 | CGTCA-motif | 158 | 163 | MeJA responsive |
| TaCCHC9 | CGTCA-motif | 603 | 608 | MeJA responsive |
| TaCCHC9 | MBS | 927 | 933 | drought responsive |
| TaCCHC9 | LTR | 671 | 677 | low-temperature responsive |
| TaCCHC9 | LTR | 981 | 987 | low-temperature responsive |
| TaCCHC9 | TGA-element | 682 | 688 | auxin responsive |
| TaCCHC9 | ARE | 714 | 720 | anaerobic induction |
| TaCCHC9 | ARE | 719 | 725 | anaerobic induction |
| TaCCHC9 | ARE | 1447 | 1453 | anaerobic induction |
| TaCCHC9 | GC-motif | 756 | 762 | anoxic specific inducibility |
| TaCCHC9 | GC-motif | 972 | 978 | anoxic specific inducibility |
| TaCCHC9 | GC-motif | 1391 | 1397 | anoxic specific inducibility |
| TaCCHC9 | ABRE | 694 | 700 | ABA responsive |
| TaCCHC9 | ABRE | 695 | 700 | ABA responsive |
| TaCCHC9 | TATA-box | 41 | 45 | conservative cis-element |
| TaCCHC9 | TATA-box | 488 | 497 | conservative cis-element |
| TaCCHC9 | TATA-box | 490 | 494 | conservative cis-element |
| TaCCHC9 | CAAT-box | 22 | 26 | conservative cis-element |
| TaCCHC9 | CAAT-box | 309 | 313 | conservative cis-element |
| TaCCHC9 | CAAT-box | 512 | 517 | conservative cis-element |
| TaCCHC9 | CAAT-box | 513 | 517 | conservative cis-element |
| TaCCHC9 | CAAT-box | 1450 | 1455 | conservative cis-element |
| TaCCHC9 | CAAT-box | 1451 | 1455 | conservative cis-element |
| TaCCHC9 | CAAT-box | 1455 | 1460 | conservative cis-element |
| TaCCHC9 | CAAT-box | 1456 | 1460 | conservative cis-element |
| TaCCHC9 | CAAT-box | 1460 | 1465 | conservative cis-element |
| TaCCHC9 | CAAT-box | 1461 | 1465 | conservative cis-element |
| TaCCHC9 | CAAT-box | 1465 | 1470 | conservative cis-element |
| TaCCHC9 | CAAT-box | 1466 | 1470 | conservative cis-element |
| TaCCHC9 | CAAT-box | 1470 | 1475 | conservative cis-element |
| TaCCHC9 | CAAT-box | 1471 | 1475 | conservative cis-element |
| TaCCHC10 | GC-motif | 1236 | 1242 | anoxic specific inducibility |
| TaCCHC10 | ABRE | 1021 | 1030 | ABA responsive |
| TaCCHC10 | ABRE | 1278 | 1283 | ABA responsive |
| TaCCHC10 | ARE | 549 | 555 | anaerobic induction |
| TaCCHC10 | ARE | 888 | 894 | anaerobic induction |
| TaCCHC10 | TGACG-motif | 1240 | 1245 | MeJA responsive |
| TaCCHC10 | CGTCA-motif | 1240 | 1245 | MeJA responsive |
| TaCCHC10 | CAAT-box | 298 | 303 | conservative cis-element |
| TaCCHC10 | CAAT-box | 491 | 495 | conservative cis-element |
| TaCCHC10 | CAAT-box | 505 | 509 | conservative cis-element |
| TaCCHC10 | CAAT-box | 538 | 542 | conservative cis-element |
| TaCCHC10 | CAAT-box | 655 | 659 | conservative cis-element |
| TaCCHC10 | CAAT-box | 754 | 758 | conservative cis-element |
| TaCCHC10 | CAAT-box | 776 | 781 | conservative cis-element |
| TaCCHC10 | CAAT-box | 868 | 875 | conservative cis-element |
| TaCCHC10 | CAAT-box | 892 | 897 | conservative cis-element |
| TaCCHC10 | CAAT-box | 1332 | 1336 | conservative cis-element |
| TaCCHC10 | TATA-box | 369 | 380 | conservative cis-element |
| TaCCHC10 | TATA-box | 373 | 379 | conservative cis-element |
| TaCCHC10 | TATA-box | 374 | 379 | conservative cis-element |
| TaCCHC10 | TATA-box | 375 | 379 | conservative cis-element |
| TaCCHC10 | TATA-box | 452 | 457 | conservative cis-element |
| TaCCHC10 | TATA-box | 453 | 457 | conservative cis-element |
| TaCCHC10 | TATA-box | 532 | 536 | conservative cis-element |
| TaCCHC10 | TATA-box | 685 | 689 | conservative cis-element |
| TaCCHC10 | TATA-box | 1411 | 1415 | conservative cis-element |
| TaCCHC11 | ARE | 859 | 865 | anaerobic induction |
| TaCCHC11 | ARE | 1332 | 1338 | anaerobic induction |
| TaCCHC11 | GC-motif | 334 | 340 | anoxic specific inducibility |
| TaCCHC11 | TC-rich repeats | 1381 | 1391 | defense and stress responsive |
| TaCCHC11 | LTR | 454 | 460 | low-temperature responsive |
| TaCCHC11 | CAAT-box | 469 | 474 | conservative cis-element |
| TaCCHC11 | CAAT-box | 506 | 511 | conservative cis-element |
| TaCCHC11 | CAAT-box | 538 | 543 | conservative cis-element |
| TaCCHC11 | CAAT-box | 609 | 613 | conservative cis-element |
| TaCCHC11 | CAAT-box | 635 | 640 | conservative cis-element |
| TaCCHC11 | CAAT-box | 768 | 773 | conservative cis-element |
| TaCCHC11 | CAAT-box | 910 | 914 | conservative cis-element |
| TaCCHC11 | CAAT-box | 1027 | 1031 | conservative cis-element |
| TaCCHC11 | CAAT-box | 1189 | 1193 | conservative cis-element |
| TaCCHC11 | CAAT-box | 1227 | 1232 | conservative cis-element |
| TaCCHC11 | CAAT-box | 1350 | 1355 | conservative cis-element |
| TaCCHC11 | CAAT-box | 1358 | 1362 | conservative cis-element |
| TaCCHC11 | CAAT-box | 1451 | 1455 | conservative cis-element |
| TaCCHC11 | CAAT-box | 1496 | 1501 | conservative cis-element |
| TaCCHC11 | TATA-box | 719 | 723 | conservative cis-element |
| TaCCHC11 | TATA-box | 749 | 756 | conservative cis-element |
| TaCCHC11 | TATA-box | 750 | 756 | conservative cis-element |
| TaCCHC11 | TATA-box | 752 | 756 | conservative cis-element |
| TaCCHC11 | TATA-box | 764 | 770 | conservative cis-element |
| TaCCHC11 | TATA-box | 765 | 769 | conservative cis-element |
| TaCCHC11 | TATA-box | 832 | 838 | conservative cis-element |
| TaCCHC11 | TATA-box | 833 | 837 | conservative cis-element |
| TaCCHC11 | TATA-box | 844 | 851 | conservative cis-element |
| TaCCHC11 | TATA-box | 1128 | 1132 | conservative cis-element |
| TaCCHC11 | TATA-box | 1241 | 1247 | conservative cis-element |
| TaCCHC11 | TATA-box | 1242 | 1246 | conservative cis-element |
| TaCCHC12 | TGA-element | 919 | 925 | auxin responsive |
| TaCCHC12 | GC-motif | 1123 | 1129 | anoxic specific inducibility |
| TaCCHC12 | ARE | 137 | 143 | anaerobic induction |
| TaCCHC12 | TGACG-motif | 673 | 678 | MeJA responsive |
| TaCCHC12 | TGACG-motif | 952 | 957 | MeJA responsive |
| TaCCHC12 | TGACG-motif | 1476 | 1481 | MeJA responsive |
| TaCCHC12 | TGACG-motif | 1491 | 1496 | MeJA responsive |
| TaCCHC12 | MBS | 961 | 967 | drought responsive |
| TaCCHC12 | CGTCA-motif | 673 | 678 | MeJA responsive |
| TaCCHC12 | CGTCA-motif | 952 | 957 | MeJA responsive |
| TaCCHC12 | CGTCA-motif | 1476 | 1481 | MeJA responsive |
| TaCCHC12 | CGTCA-motif | 1491 | 1496 | MeJA responsive |
| TaCCHC12 | CAAT-box | 113 | 117 | conservative cis-element |
| TaCCHC12 | CAAT-box | 163 | 168 | conservative cis-element |
| TaCCHC12 | CAAT-box | 164 | 168 | conservative cis-element |
| TaCCHC12 | CAAT-box | 173 | 178 | conservative cis-element |
| TaCCHC12 | CAAT-box | 174 | 178 | conservative cis-element |
| TaCCHC12 | CAAT-box | 184 | 188 | conservative cis-element |
| TaCCHC12 | CAAT-box | 371 | 375 | conservative cis-element |
| TaCCHC12 | CAAT-box | 455 | 459 | conservative cis-element |
| TaCCHC12 | CAAT-box | 642 | 646 | conservative cis-element |
| TaCCHC12 | CAAT-box | 722 | 727 | conservative cis-element |
| TaCCHC12 | CAAT-box | 723 | 727 | conservative cis-element |
| TaCCHC12 | CAAT-box | 791 | 796 | conservative cis-element |
| TaCCHC12 | CAAT-box | 792 | 796 | conservative cis-element |
| TaCCHC12 | CAAT-box | 865 | 870 | conservative cis-element |
| TaCCHC12 | CAAT-box | 866 | 870 | conservative cis-element |
| TaCCHC12 | CAAT-box | 944 | 949 | conservative cis-element |
| TaCCHC12 | CAAT-box | 1017 | 1021 | conservative cis-element |
| TaCCHC12 | TATA-box | 334 | 340 | conservative cis-element |
| TaCCHC12 | TATA-box | 335 | 341 | conservative cis-element |
| TaCCHC12 | TATA-box | 336 | 340 | conservative cis-element |
| TaCCHC12 | TATA-box | 440 | 444 | conservative cis-element |
| TaCCHC12 | TATA-box | 855 | 864 | conservative cis-element |
| TaCCHC12 | TATA-box | 861 | 865 | conservative cis-element |
| TaCCHC12 | TATA-box | 971 | 977 | conservative cis-element |
| TaCCHC12 | TATA-box | 972 | 976 | conservative cis-element |
| TaCCHC13 | TGACG-motif | 473 | 478 | MeJA responsive |
| TaCCHC13 | CGTCA-motif | 473 | 478 | MeJA responsive |
| TaCCHC13 | TCA-element | 551 | 560 | salicylic acid responsive |
| TaCCHC13 | GARE-motif | 1452 | 1459 | gibberellin responsive |
| TaCCHC13 | LTR | 593 | 599 | low-temperature responsive |
| TaCCHC13 | LTR | 1117 | 1123 | low-temperature responsive |
| TaCCHC13 | P-box | 636 | 643 | gibberellin responsive |
| TaCCHC13 | ARE | 627 | 633 | anaerobic induction |
| TaCCHC13 | ABRE | 118 | 124 | ABA responsive |
| TaCCHC13 | ABRE | 119 | 124 | ABA responsive |
| TaCCHC13 | ABRE | 299 | 304 | ABA responsive |
| TaCCHC13 | ABRE | 544 | 549 | ABA responsive |
| TaCCHC13 | TATA-box | 45 | 49 | conservative cis-element |
| TaCCHC13 | TATA-box | 229 | 235 | conservative cis-element |
| TaCCHC13 | TATA-box | 230 | 236 | conservative cis-element |
| TaCCHC13 | TATA-box | 232 | 236 | conservative cis-element |
| TaCCHC13 | TATA-box | 315 | 321 | conservative cis-element |
| TaCCHC13 | TATA-box | 316 | 320 | conservative cis-element |
| TaCCHC13 | TATA-box | 329 | 338 | conservative cis-element |
| TaCCHC13 | TATA-box | 331 | 337 | conservative cis-element |
| TaCCHC13 | TATA-box | 332 | 337 | conservative cis-element |
| TaCCHC13 | TATA-box | 333 | 337 | conservative cis-element |
| TaCCHC13 | TATA-box | 492 | 496 | conservative cis-element |
| TaCCHC13 | TATA-box | 1091 | 1095 | conservative cis-element |
| TaCCHC13 | CAAT-box | 11 | 16 | conservative cis-element |
| TaCCHC13 | CAAT-box | 102 | 106 | conservative cis-element |
| TaCCHC13 | CAAT-box | 111 | 115 | conservative cis-element |
| TaCCHC13 | CAAT-box | 187 | 191 | conservative cis-element |
| TaCCHC13 | CAAT-box | 206 | 210 | conservative cis-element |
| TaCCHC13 | CAAT-box | 240 | 244 | conservative cis-element |
| TaCCHC13 | CAAT-box | 251 | 255 | conservative cis-element |
| TaCCHC13 | CAAT-box | 275 | 279 | conservative cis-element |
| TaCCHC13 | CAAT-box | 325 | 330 | conservative cis-element |
| TaCCHC13 | CAAT-box | 342 | 347 | conservative cis-element |
| TaCCHC13 | CAAT-box | 370 | 374 | conservative cis-element |
| TaCCHC13 | CAAT-box | 400 | 404 | conservative cis-element |
| TaCCHC13 | CAAT-box | 619 | 624 | conservative cis-element |
| TaCCHC13 | CAAT-box | 660 | 664 | conservative cis-element |
| TaCCHC13 | CAAT-box | 662 | 666 | conservative cis-element |
| TaCCHC13 | CAAT-box | 674 | 678 | conservative cis-element |
| TaCCHC13 | CAAT-box | 676 | 681 | conservative cis-element |
| TaCCHC13 | CAAT-box | 715 | 720 | conservative cis-element |
| TaCCHC13 | CAAT-box | 758 | 763 | conservative cis-element |
| TaCCHC13 | CAAT-box | 764 | 768 | conservative cis-element |
| TaCCHC13 | CAAT-box | 848 | 853 | conservative cis-element |
| TaCCHC13 | CAAT-box | 849 | 853 | conservative cis-element |
| TaCCHC13 | CAAT-box | 970 | 974 | conservative cis-element |
| TaCCHC13 | CAAT-box | 988 | 992 | conservative cis-element |
| TaCCHC13 | CAAT-box | 1096 | 1100 | conservative cis-element |
| TaCCHC13 | CAAT-box | 1153 | 1157 | conservative cis-element |
| TaCCHC13 | CAAT-box | 1168 | 1172 | conservative cis-element |
| TaCCHC13 | CAAT-box | 1283 | 1288 | conservative cis-element |
| TaCCHC13 | CAAT-box | 1284 | 1288 | conservative cis-element |
| TaCCHC13 | CAAT-box | 1286 | 1291 | conservative cis-element |
| TaCCHC13 | CAAT-box | 1347 | 1351 | conservative cis-element |
| TaCCHC13 | CAAT-box | 1371 | 1375 | conservative cis-element |
| TaCCHC13 | CAAT-box | 1399 | 1404 | conservative cis-element |
| TaCCHC14 | ERE | 7 | 15 | ethylene responsive |
| TaCCHC14 | TGA-element | 1062 | 1068 | auxin responsive |
| TaCCHC14 | GC-motif | 1363 | 1369 | anoxic specific inducibility |
| TaCCHC14 | ABRE | 594 | 599 | ABA responsive |
| TaCCHC14 | ABRE | 760 | 768 | ABA responsive |
| TaCCHC14 | ABRE | 761 | 766 | ABA responsive |
| TaCCHC14 | ABRE | 1097 | 1102 | ABA responsive |
| TaCCHC14 | ABRE | 1147 | 1155 | ABA responsive |
| TaCCHC14 | ABRE | 1148 | 1153 | ABA responsive |
| TaCCHC14 | AuxRR-core | 951 | 958 | auxin responsive |
| TaCCHC14 | TGACG-motif | 272 | 277 | MeJA responsive |
| TaCCHC14 | TGACG-motif | 502 | 507 | MeJA responsive |
| TaCCHC14 | TCA-element | 881 | 890 | salicylic acid responsive |
| TaCCHC14 | TCA-element | 1289 | 1298 | salicylic acid responsive |
| TaCCHC14 | CGTCA-motif | 272 | 277 | MeJA responsive |
| TaCCHC14 | CGTCA-motif | 502 | 507 | MeJA responsive |
| TaCCHC14 | LTR | 185 | 191 | low-temperature responsive |
| TaCCHC14 | LTR | 1486 | 1492 | low-temperature responsive |
| TaCCHC14 | CAAT-box | 19 | 24 | conservative cis-element |
| TaCCHC14 | CAAT-box | 31 | 36 | conservative cis-element |
| TaCCHC14 | CAAT-box | 37 | 42 | conservative cis-element |
| TaCCHC14 | CAAT-box | 82 | 86 | conservative cis-element |
| TaCCHC14 | CAAT-box | 288 | 292 | conservative cis-element |
| TaCCHC14 | CAAT-box | 435 | 439 | conservative cis-element |
| TaCCHC14 | CAAT-box | 456 | 460 | conservative cis-element |
| TaCCHC14 | CAAT-box | 621 | 625 | conservative cis-element |
| TaCCHC14 | CAAT-box | 809 | 814 | conservative cis-element |
| TaCCHC14 | CAAT-box | 836 | 840 | conservative cis-element |
| TaCCHC14 | CAAT-box | 871 | 875 | conservative cis-element |
| TaCCHC14 | CAAT-box | 873 | 877 | conservative cis-element |
| TaCCHC14 | CAAT-box | 936 | 940 | conservative cis-element |
| TaCCHC14 | CAAT-box | 966 | 970 | conservative cis-element |
| TaCCHC14 | CAAT-box | 1141 | 1146 | conservative cis-element |
| TaCCHC14 | CAAT-box | 1205 | 1210 | conservative cis-element |
| TaCCHC14 | CAAT-box | 1216 | 1221 | conservative cis-element |
| TaCCHC14 | TATA-box | 254 | 259 | conservative cis-element |
| TaCCHC14 | TATA-box | 255 | 259 | conservative cis-element |
| TaCCHC14 | TATA-box | 341 | 345 | conservative cis-element |
| TaCCHC14 | TATA-box | 415 | 419 | conservative cis-element |
| TaCCHC14 | TATA-box | 440 | 445 | conservative cis-element |
| TaCCHC14 | TATA-box | 441 | 445 | conservative cis-element |
| TaCCHC14 | TATA-box | 458 | 464 | conservative cis-element |
| TaCCHC14 | TATA-box | 460 | 464 | conservative cis-element |
| TaCCHC14 | TATA-box | 605 | 609 | conservative cis-element |
| TaCCHC14 | TATA-box | 679 | 683 | conservative cis-element |
| TaCCHC14 | TATA-box | 787 | 793 | conservative cis-element |
| TaCCHC14 | TATA-box | 789 | 795 | conservative cis-element |
| TaCCHC14 | TATA-box | 790 | 796 | conservative cis-element |
| TaCCHC14 | TATA-box | 791 | 795 | conservative cis-element |
| TaCCHC14 | TATA-box | 840 | 846 | conservative cis-element |
| TaCCHC14 | TATA-box | 841 | 846 | conservative cis-element |
| TaCCHC14 | TATA-box | 842 | 846 | conservative cis-element |
| TaCCHC14 | TATA-box | 886 | 892 | conservative cis-element |
| TaCCHC14 | TATA-box | 887 | 892 | conservative cis-element |
| TaCCHC14 | TATA-box | 888 | 892 | conservative cis-element |
| TaCCHC14 | TATA-box | 1240 | 1246 | conservative cis-element |
| TaCCHC14 | TATA-box | 1241 | 1246 | conservative cis-element |
| TaCCHC14 | TATA-box | 1242 | 1246 | conservative cis-element |
| TaCCHC14 | TATA-box | 1409 | 1417 | conservative cis-element |
| TaCCHC14 | TATA-box | 1410 | 1417 | conservative cis-element |
| TaCCHC14 | TATA-box | 1411 | 1417 | conservative cis-element |
| TaCCHC14 | TATA-box | 1412 | 1417 | conservative cis-element |
| TaCCHC14 | TATA-box | 1413 | 1417 | conservative cis-element |
| TaCCHC15 | CGTCA-motif | 500 | 505 | MeJA responsive |
| TaCCHC15 | CGTCA-motif | 646 | 651 | MeJA responsive |
| TaCCHC15 | CGTCA-motif | 1270 | 1275 | MeJA responsive |
| TaCCHC15 | MBS | 765 | 771 | drought responsive |
| TaCCHC15 | TGACG-motif | 500 | 505 | MeJA responsive |
| TaCCHC15 | TGACG-motif | 646 | 651 | MeJA responsive |
| TaCCHC15 | TGACG-motif | 1270 | 1275 | MeJA responsive |
| TaCCHC15 | ARE | 222 | 228 | anaerobic induction |
| TaCCHC15 | ARE | 908 | 914 | anaerobic induction |
| TaCCHC15 | ABRE | 1051 | 1060 | ABA responsive |
| TaCCHC15 | ABRE | 1300 | 1305 | ABA responsive |
| TaCCHC15 | GC-motif | 1266 | 1272 | anoxic specific inducibility |
| TaCCHC15 | TATA-box | 14 | 21 | conservative cis-element |
| TaCCHC15 | TATA-box | 15 | 21 | conservative cis-element |
| TaCCHC15 | TATA-box | 16 | 21 | conservative cis-element |
| TaCCHC15 | TATA-box | 17 | 21 | conservative cis-element |
| TaCCHC15 | TATA-box | 353 | 359 | conservative cis-element |
| TaCCHC15 | TATA-box | 354 | 359 | conservative cis-element |
| TaCCHC15 | TATA-box | 355 | 359 | conservative cis-element |
| TaCCHC15 | TATA-box | 415 | 421 | conservative cis-element |
| TaCCHC15 | TATA-box | 416 | 420 | conservative cis-element |
| TaCCHC15 | TATA-box | 538 | 544 | conservative cis-element |
| TaCCHC15 | TATA-box | 539 | 543 | conservative cis-element |
| TaCCHC15 | TATA-box | 617 | 621 | conservative cis-element |
| TaCCHC15 | TATA-box | 1414 | 1420 | conservative cis-element |
| TaCCHC15 | TATA-box | 1415 | 1419 | conservative cis-element |
| TaCCHC15 | CAAT-box | 1 | 5 | conservative cis-element |
| TaCCHC15 | CAAT-box | 266 | 270 | conservative cis-element |
| TaCCHC15 | CAAT-box | 343 | 347 | conservative cis-element |
| TaCCHC15 | CAAT-box | 378 | 383 | conservative cis-element |
| TaCCHC15 | CAAT-box | 381 | 385 | conservative cis-element |
| TaCCHC15 | CAAT-box | 599 | 604 | conservative cis-element |
| TaCCHC15 | CAAT-box | 716 | 720 | conservative cis-element |
| TaCCHC15 | CAAT-box | 731 | 735 | conservative cis-element |
| TaCCHC15 | CAAT-box | 752 | 756 | conservative cis-element |
| TaCCHC15 | CAAT-box | 756 | 760 | conservative cis-element |
| TaCCHC15 | CAAT-box | 771 | 775 | conservative cis-element |
| TaCCHC15 | CAAT-box | 792 | 797 | conservative cis-element |
| TaCCHC15 | CAAT-box | 883 | 890 | conservative cis-element |
| TaCCHC15 | CAAT-box | 912 | 917 | conservative cis-element |
| TaCCHC15 | CAAT-box | 1411 | 1416 | conservative cis-element |
| TaCCHC15 | CAAT-box | 1412 | 1416 | conservative cis-element |
| TaCCHC16 | ARE | 976 | 982 | anaerobic induction |
| TaCCHC16 | ARE | 1459 | 1465 | anaerobic induction |
| TaCCHC16 | GC-motif | 203 | 209 | anoxic specific inducibility |
| TaCCHC16 | GC-motif | 316 | 322 | anoxic specific inducibility |
| TaCCHC16 | ERE | 1029 | 1037 | ethylene responsive |
| TaCCHC16 | ERE | 1031 | 1039 | ethylene responsive |
| TaCCHC16 | CGTCA-motif | 158 | 163 | MeJA responsive |
| TaCCHC16 | MBS | 504 | 510 | drought responsive |
| TaCCHC16 | MBS | 726 | 732 | drought responsive |
| TaCCHC16 | LTR | 219 | 225 | low-temperature responsive |
| TaCCHC16 | LTR | 234 | 240 | low-temperature responsive |
| TaCCHC16 | LTR | 241 | 247 | low-temperature responsive |
| TaCCHC16 | TGACG-motif | 158 | 163 | MeJA responsive |
| TaCCHC16 | CAAT-box | 325 | 330 | conservative cis-element |
| TaCCHC16 | CAAT-box | 396 | 400 | conservative cis-element |
| TaCCHC16 | CAAT-box | 422 | 427 | conservative cis-element |
| TaCCHC16 | CAAT-box | 588 | 592 | conservative cis-element |
| TaCCHC16 | CAAT-box | 714 | 719 | conservative cis-element |
| TaCCHC16 | CAAT-box | 801 | 805 | conservative cis-element |
| TaCCHC16 | CAAT-box | 829 | 833 | conservative cis-element |
| TaCCHC16 | CAAT-box | 973 | 977 | conservative cis-element |
| TaCCHC16 | CAAT-box | 1002 | 1007 | conservative cis-element |
| TaCCHC16 | CAAT-box | 1007 | 1011 | conservative cis-element |
| TaCCHC16 | CAAT-box | 1017 | 1022 | conservative cis-element |
| TaCCHC16 | CAAT-box | 1043 | 1047 | conservative cis-element |
| TaCCHC16 | CAAT-box | 1167 | 1171 | conservative cis-element |
| TaCCHC16 | CAAT-box | 1203 | 1207 | conservative cis-element |
| TaCCHC16 | CAAT-box | 1334 | 1338 | conservative cis-element |
| TaCCHC16 | CAAT-box | 1373 | 1378 | conservative cis-element |
| TaCCHC16 | CAAT-box | 1448 | 1453 | conservative cis-element |
| TaCCHC16 | CAAT-box | 1485 | 1489 | conservative cis-element |
| TaCCHC16 | TATA-box | 443 | 449 | conservative cis-element |
| TaCCHC16 | TATA-box | 445 | 449 | conservative cis-element |
| TaCCHC16 | TATA-box | 491 | 497 | conservative cis-element |
| TaCCHC16 | TATA-box | 493 | 497 | conservative cis-element |
| TaCCHC16 | TATA-box | 572 | 578 | conservative cis-element |
| TaCCHC16 | TATA-box | 573 | 577 | conservative cis-element |
| TaCCHC16 | TATA-box | 634 | 641 | conservative cis-element |
| TaCCHC16 | TATA-box | 635 | 641 | conservative cis-element |
| TaCCHC16 | TATA-box | 636 | 642 | conservative cis-element |
| TaCCHC16 | TATA-box | 637 | 641 | conservative cis-element |
| TaCCHC16 | TATA-box | 666 | 673 | conservative cis-element |
| TaCCHC16 | TATA-box | 667 | 673 | conservative cis-element |
| TaCCHC16 | TATA-box | 668 | 673 | conservative cis-element |
| TaCCHC16 | TATA-box | 669 | 673 | conservative cis-element |
| TaCCHC16 | TATA-box | 839 | 843 | conservative cis-element |
| TaCCHC16 | TATA-box | 1097 | 1104 | conservative cis-element |
| TaCCHC16 | TATA-box | 1098 | 1104 | conservative cis-element |
| TaCCHC16 | TATA-box | 1099 | 1105 | conservative cis-element |
| TaCCHC16 | TATA-box | 1100 | 1104 | conservative cis-element |
| TaCCHC16 | TATA-box | 1140 | 1146 | conservative cis-element |
| TaCCHC16 | TATA-box | 1141 | 1145 | conservative cis-element |
| TaCCHC16 | TATA-box | 1270 | 1274 | conservative cis-element |
| TaCCHC17 | TGA-element | 519 | 525 | auxin responsive |
| TaCCHC17 | TGA-element | 664 | 670 | auxin responsive |
| TaCCHC17 | ABRE | 59 | 64 | ABA responsive |
| TaCCHC17 | ABRE | 100 | 105 | ABA responsive |
| TaCCHC17 | ABRE | 389 | 396 | ABA responsive |
| TaCCHC17 | ABRE | 945 | 950 | ABA responsive |
| TaCCHC17 | ABRE | 1037 | 1043 | ABA responsive |
| TaCCHC17 | ABRE | 1038 | 1043 | ABA responsive |
| TaCCHC17 | GC-motif | 1293 | 1299 | anoxic specific inducibility |
| TaCCHC17 | ARE | 47 | 53 | anaerobic induction |
| TaCCHC17 | TGACG-motif | 969 | 974 | MeJA responsive |
| TaCCHC17 | AuxRR-core | 1332 | 1339 | auxin responsive |
| TaCCHC17 | MBS | 834 | 840 | drought responsive |
| TaCCHC17 | LTR | 1301 | 1307 | low-temperature responsive |
| TaCCHC17 | CGTCA-motif | 969 | 974 | MeJA responsive |
| TaCCHC17 | CAAT-box | 302 | 306 | conservative cis-element |
| TaCCHC17 | CAAT-box | 480 | 485 | conservative cis-element |
| TaCCHC17 | CAAT-box | 539 | 546 | conservative cis-element |
| TaCCHC17 | CAAT-box | 564 | 568 | conservative cis-element |
| TaCCHC17 | CAAT-box | 587 | 591 | conservative cis-element |
| TaCCHC17 | CAAT-box | 648 | 653 | conservative cis-element |
| TaCCHC17 | CAAT-box | 709 | 713 | conservative cis-element |
| TaCCHC17 | CAAT-box | 731 | 735 | conservative cis-element |
| TaCCHC17 | CAAT-box | 755 | 759 | conservative cis-element |
| TaCCHC17 | CAAT-box | 864 | 868 | conservative cis-element |
| TaCCHC17 | CAAT-box | 1019 | 1023 | conservative cis-element |
| TaCCHC17 | CAAT-box | 1104 | 1108 | conservative cis-element |
| TaCCHC17 | CAAT-box | 1358 | 1362 | conservative cis-element |
| TaCCHC17 | CAAT-box | 1457 | 1462 | conservative cis-element |
| TaCCHC18 | ARE | 372 | 378 | anaerobic induction |
| TaCCHC18 | ARE | 748 | 754 | anaerobic induction |
| TaCCHC18 | WUN-motif | 182 | 191 | wound responsive |
| TaCCHC18 | WUN-motif | 927 | 936 | wound responsive |
| TaCCHC18 | CGTCA-motif | 881 | 886 | MeJA responsive |
| TaCCHC18 | TCA-element | 538 | 547 | salicylic acid responsive |
| TaCCHC18 | LTR | 1116 | 1122 | low-temperature responsive |
| TaCCHC18 | GARE-motif | 1087 | 1094 | gibberellin responsive |
| TaCCHC18 | TGACG-motif | 881 | 886 | MeJA responsive |
| TaCCHC18 | CAAT-box | 170 | 175 | conservative cis-element |
| TaCCHC18 | CAAT-box | 181 | 186 | conservative cis-element |
| TaCCHC18 | CAAT-box | 327 | 332 | conservative cis-element |
| TaCCHC18 | CAAT-box | 408 | 412 | conservative cis-element |
| TaCCHC18 | CAAT-box | 510 | 515 | conservative cis-element |
| TaCCHC18 | CAAT-box | 515 | 519 | conservative cis-element |
| TaCCHC18 | CAAT-box | 530 | 535 | conservative cis-element |
| TaCCHC18 | CAAT-box | 583 | 587 | conservative cis-element |
| TaCCHC18 | CAAT-box | 677 | 682 | conservative cis-element |
| TaCCHC18 | CAAT-box | 686 | 690 | conservative cis-element |
| TaCCHC18 | CAAT-box | 714 | 718 | conservative cis-element |
| TaCCHC18 | CAAT-box | 774 | 778 | conservative cis-element |
| TaCCHC18 | CAAT-box | 852 | 856 | conservative cis-element |
| TaCCHC18 | CAAT-box | 859 | 863 | conservative cis-element |
| TaCCHC18 | CAAT-box | 864 | 868 | conservative cis-element |
| TaCCHC18 | CAAT-box | 889 | 894 | conservative cis-element |
| TaCCHC18 | CAAT-box | 982 | 986 | conservative cis-element |
| TaCCHC18 | CAAT-box | 996 | 1001 | conservative cis-element |
| TaCCHC18 | CAAT-box | 1103 | 1107 | conservative cis-element |
| TaCCHC18 | CAAT-box | 1132 | 1136 | conservative cis-element |
| TaCCHC18 | CAAT-box | 1179 | 1183 | conservative cis-element |
| TaCCHC18 | CAAT-box | 1333 | 1337 | conservative cis-element |
| TaCCHC18 | CAAT-box | 1391 | 1395 | conservative cis-element |
| TaCCHC18 | TATA-box | 30 | 37 | conservative cis-element |
| TaCCHC18 | TATA-box | 496 | 500 | conservative cis-element |
| TaCCHC18 | TATA-box | 562 | 566 | conservative cis-element |
| TaCCHC18 | TATA-box | 593 | 601 | conservative cis-element |
| TaCCHC18 | TATA-box | 637 | 643 | conservative cis-element |
| TaCCHC18 | TATA-box | 639 | 643 | conservative cis-element |
| TaCCHC18 | TATA-box | 673 | 678 | conservative cis-element |
| TaCCHC18 | TATA-box | 674 | 678 | conservative cis-element |
| TaCCHC18 | TATA-box | 842 | 849 | conservative cis-element |
| TaCCHC18 | TATA-box | 843 | 849 | conservative cis-element |
| TaCCHC18 | TATA-box | 844 | 850 | conservative cis-element |
| TaCCHC18 | TATA-box | 845 | 849 | conservative cis-element |
| TaCCHC18 | TATA-box | 870 | 874 | conservative cis-element |
| TaCCHC18 | TATA-box | 886 | 890 | conservative cis-element |
| TaCCHC19 | MBS | 209 | 215 | drought responsive |
| TaCCHC19 | ABRE | 343 | 352 | ABA responsive |
| TaCCHC19 | ABRE | 552 | 561 | ABA responsive |
| TaCCHC19 | ABRE | 1081 | 1090 | ABA responsive |
| TaCCHC19 | ABRE | 1198 | 1204 | ABA responsive |
| TaCCHC19 | ABRE | 1199 | 1204 | ABA responsive |
| TaCCHC19 | ABRE | 1472 | 1477 | ABA responsive |
| TaCCHC19 | GC-motif | 479 | 485 | anoxic specific inducibility |
| TaCCHC19 | TATA-box | 253 | 259 | conservative cis-element |
| TaCCHC19 | TATA-box | 254 | 258 | conservative cis-element |
| TaCCHC19 | TATA-box | 713 | 722 | conservative cis-element |
| TaCCHC19 | TATA-box | 949 | 953 | conservative cis-element |
| TaCCHC19 | TATA-box | 995 | 1001 | conservative cis-element |
| TaCCHC19 | TATA-box | 997 | 1001 | conservative cis-element |
| TaCCHC19 | TATA-box | 1044 | 1050 | conservative cis-element |
| TaCCHC19 | TATA-box | 1046 | 1050 | conservative cis-element |
| TaCCHC19 | TATA-box | 1124 | 1128 | conservative cis-element |
| TaCCHC19 | TATA-box | 1309 | 1315 | conservative cis-element |
| TaCCHC19 | TATA-box | 1310 | 1316 | conservative cis-element |
| TaCCHC19 | TATA-box | 1312 | 1316 | conservative cis-element |
| TaCCHC19 | TATA-box | 1394 | 1400 | conservative cis-element |
| TaCCHC19 | TATA-box | 1395 | 1399 | conservative cis-element |
| TaCCHC19 | TATA-box | 1410 | 1416 | conservative cis-element |
| TaCCHC19 | TATA-box | 1412 | 1416 | conservative cis-element |
| TaCCHC19 | CAAT-box | 21 | 25 | conservative cis-element |
| TaCCHC19 | CAAT-box | 47 | 51 | conservative cis-element |
| TaCCHC19 | CAAT-box | 280 | 284 | conservative cis-element |
| TaCCHC19 | CAAT-box | 492 | 496 | conservative cis-element |
| TaCCHC19 | CAAT-box | 702 | 707 | conservative cis-element |
| TaCCHC19 | CAAT-box | 780 | 785 | conservative cis-element |
| TaCCHC19 | CAAT-box | 859 | 864 | conservative cis-element |
| TaCCHC19 | CAAT-box | 889 | 894 | conservative cis-element |
| TaCCHC19 | CAAT-box | 899 | 903 | conservative cis-element |
| TaCCHC19 | CAAT-box | 958 | 963 | conservative cis-element |
| TaCCHC19 | CAAT-box | 979 | 983 | conservative cis-element |
| TaCCHC19 | CAAT-box | 1004 | 1008 | conservative cis-element |
| TaCCHC19 | CAAT-box | 1042 | 1046 | conservative cis-element |
| TaCCHC19 | CAAT-box | 1090 | 1095 | conservative cis-element |
| TaCCHC19 | CAAT-box | 1182 | 1186 | conservative cis-element |
| TaCCHC19 | CAAT-box | 1191 | 1195 | conservative cis-element |
| TaCCHC19 | CAAT-box | 1320 | 1324 | conservative cis-element |
| TaCCHC19 | CAAT-box | 1331 | 1335 | conservative cis-element |
| TaCCHC19 | CAAT-box | 1398 | 1402 | conservative cis-element |
| TaCCHC19 | CAAT-box | 1404 | 1409 | conservative cis-element |
| TaCCHC19 | CAAT-box | 1421 | 1426 | conservative cis-element |
| TaCCHC19 | CAAT-box | 1449 | 1453 | conservative cis-element |
| TaCCHC19 | CAAT-box | 1479 | 1483 | conservative cis-element |
| TaCCHC20 | GC-motif | 1363 | 1369 | anoxic specific inducibility |
| TaCCHC20 | ABRE | 760 | 768 | ABA responsive |
| TaCCHC20 | ABRE | 761 | 766 | ABA responsive |
| TaCCHC20 | ABRE | 1094 | 1099 | ABA responsive |
| TaCCHC20 | ABRE | 1144 | 1152 | ABA responsive |
| TaCCHC20 | ABRE | 1145 | 1150 | ABA responsive |
| TaCCHC20 | TGA-element | 1059 | 1065 | auxin responsive |
| TaCCHC20 | LTR | 1486 | 1492 | low-temperature responsive |
| TaCCHC20 | TCA-element | 1283 | 1292 | salicylic acid responsive |
| TaCCHC20 | CGTCA-motif | 431 | 436 | MeJA responsive |
| TaCCHC20 | TGACG-motif | 431 | 436 | MeJA responsive |
| TaCCHC20 | AuxRR-core | 948 | 955 | auxin responsive |
| TaCCHC20 | CAAT-box | 1 | 6 | conservative cis-element |
| TaCCHC20 | CAAT-box | 110 | 114 | conservative cis-element |
| TaCCHC20 | CAAT-box | 247 | 251 | conservative cis-element |
| TaCCHC20 | CAAT-box | 290 | 294 | conservative cis-element |
| TaCCHC20 | CAAT-box | 368 | 372 | conservative cis-element |
| TaCCHC20 | CAAT-box | 608 | 612 | conservative cis-element |
| TaCCHC20 | CAAT-box | 633 | 638 | conservative cis-element |
| TaCCHC20 | CAAT-box | 809 | 814 | conservative cis-element |
| TaCCHC20 | CAAT-box | 812 | 816 | conservative cis-element |
| TaCCHC20 | CAAT-box | 867 | 872 | conservative cis-element |
| TaCCHC20 | CAAT-box | 868 | 872 | conservative cis-element |
| TaCCHC20 | CAAT-box | 870 | 874 | conservative cis-element |
| TaCCHC20 | CAAT-box | 891 | 895 | conservative cis-element |
| TaCCHC20 | CAAT-box | 903 | 908 | conservative cis-element |
| TaCCHC20 | CAAT-box | 940 | 944 | conservative cis-element |
| TaCCHC20 | CAAT-box | 963 | 967 | conservative cis-element |
| TaCCHC20 | CAAT-box | 1202 | 1207 | conservative cis-element |
| TaCCHC20 | TATA-box | 58 | 65 | conservative cis-element |
| TaCCHC20 | TATA-box | 118 | 123 | conservative cis-element |
| TaCCHC20 | TATA-box | 119 | 123 | conservative cis-element |
| TaCCHC20 | TATA-box | 205 | 209 | conservative cis-element |
| TaCCHC20 | TATA-box | 213 | 218 | conservative cis-element |
| TaCCHC20 | TATA-box | 214 | 218 | conservative cis-element |
| TaCCHC20 | TATA-box | 267 | 273 | conservative cis-element |
| TaCCHC20 | TATA-box | 268 | 272 | conservative cis-element |
| TaCCHC20 | TATA-box | 331 | 335 | conservative cis-element |
| TaCCHC20 | TATA-box | 344 | 350 | conservative cis-element |
| TaCCHC20 | TATA-box | 346 | 350 | conservative cis-element |
| TaCCHC20 | TATA-box | 388 | 393 | conservative cis-element |
| TaCCHC20 | TATA-box | 389 | 393 | conservative cis-element |
| TaCCHC20 | TATA-box | 552 | 558 | conservative cis-element |
| TaCCHC20 | TATA-box | 554 | 558 | conservative cis-element |
| TaCCHC20 | TATA-box | 610 | 616 | conservative cis-element |
| TaCCHC20 | TATA-box | 611 | 616 | conservative cis-element |
| TaCCHC20 | TATA-box | 612 | 616 | conservative cis-element |
| TaCCHC20 | TATA-box | 661 | 665 | conservative cis-element |
| TaCCHC20 | TATA-box | 679 | 683 | conservative cis-element |
| TaCCHC20 | TATA-box | 787 | 793 | conservative cis-element |
| TaCCHC20 | TATA-box | 789 | 795 | conservative cis-element |
| TaCCHC20 | TATA-box | 790 | 796 | conservative cis-element |
| TaCCHC20 | TATA-box | 791 | 795 | conservative cis-element |
| TaCCHC20 | TATA-box | 876 | 882 | conservative cis-element |
| TaCCHC20 | TATA-box | 877 | 881 | conservative cis-element |
| TaCCHC20 | TATA-box | 883 | 889 | conservative cis-element |
| TaCCHC20 | TATA-box | 884 | 889 | conservative cis-element |
| TaCCHC20 | TATA-box | 885 | 889 | conservative cis-element |
| TaCCHC20 | TATA-box | 894 | 898 | conservative cis-element |
| TaCCHC20 | TATA-box | 933 | 939 | conservative cis-element |
| TaCCHC20 | TATA-box | 934 | 939 | conservative cis-element |
| TaCCHC20 | TATA-box | 935 | 939 | conservative cis-element |
| TaCCHC20 | TATA-box | 1125 | 1129 | conservative cis-element |
| TaCCHC20 | TATA-box | 1193 | 1199 | conservative cis-element |
| TaCCHC20 | TATA-box | 1194 | 1200 | conservative cis-element |
| TaCCHC20 | TATA-box | 1196 | 1200 | conservative cis-element |
| TaCCHC20 | TATA-box | 1237 | 1243 | conservative cis-element |
| TaCCHC20 | TATA-box | 1238 | 1243 | conservative cis-element |
| TaCCHC20 | TATA-box | 1239 | 1243 | conservative cis-element |
| TaCCHC20 | TATA-box | 1409 | 1417 | conservative cis-element |
| TaCCHC20 | TATA-box | 1410 | 1417 | conservative cis-element |
| TaCCHC20 | TATA-box | 1411 | 1417 | conservative cis-element |
| TaCCHC20 | TATA-box | 1412 | 1417 | conservative cis-element |
| TaCCHC20 | TATA-box | 1413 | 1417 | conservative cis-element |
| TaCCHC21 | ARE | 912 | 918 | anaerobic induction |
| TaCCHC21 | ARE | 1324 | 1330 | anaerobic induction |
| TaCCHC21 | ABRE | 85 | 90 | ABA responsive |
| TaCCHC21 | ABRE | 512 | 517 | ABA responsive |
| TaCCHC21 | ABRE | 661 | 666 | ABA responsive |
| TaCCHC21 | ABRE | 796 | 801 | ABA responsive |
| TaCCHC21 | TC-rich repeats | 1220 | 1229 | defense and stress responsive |
| TaCCHC21 | TGA-element | 460 | 466 | auxin responsive |
| TaCCHC21 | CGTCA-motif | 392 | 397 | MeJA responsive |
| TaCCHC21 | CGTCA-motif | 659 | 664 | MeJA responsive |
| TaCCHC21 | CGTCA-motif | 881 | 886 | MeJA responsive |
| TaCCHC21 | MBS | 897 | 903 | drought responsive |
| TaCCHC21 | GARE-motif | 454 | 461 | gibberellin responsive |
| TaCCHC21 | TGACG-motif | 392 | 397 | MeJA responsive |
| TaCCHC21 | TGACG-motif | 659 | 664 | MeJA responsive |
| TaCCHC21 | TGACG-motif | 881 | 886 | MeJA responsive |
| TaCCHC21 | CAAT-box | 415 | 420 | conservative cis-element |
| TaCCHC21 | CAAT-box | 428 | 432 | conservative cis-element |
| TaCCHC21 | CAAT-box | 545 | 550 | conservative cis-element |
| TaCCHC21 | CAAT-box | 654 | 658 | conservative cis-element |
| TaCCHC21 | CAAT-box | 763 | 768 | conservative cis-element |
| TaCCHC21 | CAAT-box | 971 | 976 | conservative cis-element |
| TaCCHC21 | CAAT-box | 1049 | 1054 | conservative cis-element |
| TaCCHC21 | CAAT-box | 1071 | 1076 | conservative cis-element |
| TaCCHC21 | CAAT-box | 1152 | 1156 | conservative cis-element |
| TaCCHC21 | CAAT-box | 1167 | 1172 | conservative cis-element |
| TaCCHC21 | CAAT-box | 1170 | 1174 | conservative cis-element |
| TaCCHC21 | CAAT-box | 1191 | 1196 | conservative cis-element |
| TaCCHC21 | CAAT-box | 1211 | 1215 | conservative cis-element |
| TaCCHC21 | CAAT-box | 1274 | 1278 | conservative cis-element |
| TaCCHC21 | CAAT-box | 1291 | 1295 | conservative cis-element |
| TaCCHC21 | CAAT-box | 1344 | 1348 | conservative cis-element |
| TaCCHC21 | TATA-box | 289 | 295 | conservative cis-element |
| TaCCHC21 | TATA-box | 290 | 295 | conservative cis-element |
| TaCCHC21 | TATA-box | 291 | 295 | conservative cis-element |
| TaCCHC21 | TATA-box | 335 | 339 | conservative cis-element |
| TaCCHC21 | TATA-box | 357 | 361 | conservative cis-element |
| TaCCHC21 | TATA-box | 409 | 413 | conservative cis-element |
| TaCCHC21 | TATA-box | 441 | 445 | conservative cis-element |
| TaCCHC21 | TATA-box | 597 | 601 | conservative cis-element |
| TaCCHC21 | TATA-box | 1243 | 1250 | conservative cis-element |
| TaCCHC21 | TATA-box | 1244 | 1250 | conservative cis-element |
| TaCCHC21 | TATA-box | 1245 | 1250 | conservative cis-element |
| TaCCHC21 | TATA-box | 1246 | 1250 | conservative cis-element |
| TaCCHC22 | TGA-element | 726 | 732 | auxin responsive |
| TaCCHC22 | ARE | 599 | 605 | anaerobic induction |
| TaCCHC22 | ARE | 903 | 909 | anaerobic induction |
| TaCCHC22 | ABRE | 936 | 941 | ABA responsive |
| TaCCHC22 | ABRE | 1188 | 1197 | ABA responsive |
| TaCCHC22 | ABRE | 1190 | 1196 | ABA responsive |
| TaCCHC22 | ABRE | 1191 | 1196 | ABA responsive |
| TaCCHC22 | TGACG-motif | 743 | 748 | MeJA responsive |
| TaCCHC22 | TGACG-motif | 953 | 958 | MeJA responsive |
| TaCCHC22 | CGTCA-motif | 743 | 748 | MeJA responsive |
| TaCCHC22 | CGTCA-motif | 953 | 958 | MeJA responsive |
| TaCCHC22 | LTR | 1439 | 1445 | low-temperature responsive |
| TaCCHC22 | LTR | 1479 | 1485 | low-temperature responsive |
| TaCCHC22 | CAAT-box | 75 | 79 | conservative cis-element |
| TaCCHC22 | CAAT-box | 77 | 81 | conservative cis-element |
| TaCCHC22 | CAAT-box | 118 | 122 | conservative cis-element |
| TaCCHC22 | CAAT-box | 132 | 136 | conservative cis-element |
| TaCCHC22 | CAAT-box | 139 | 143 | conservative cis-element |
| TaCCHC22 | CAAT-box | 171 | 176 | conservative cis-element |
| TaCCHC22 | CAAT-box | 178 | 182 | conservative cis-element |
| TaCCHC22 | CAAT-box | 201 | 205 | conservative cis-element |
| TaCCHC22 | CAAT-box | 203 | 207 | conservative cis-element |
| TaCCHC22 | CAAT-box | 236 | 240 | conservative cis-element |
| TaCCHC22 | CAAT-box | 272 | 277 | conservative cis-element |
| TaCCHC22 | CAAT-box | 765 | 769 | conservative cis-element |
| TaCCHC22 | CAAT-box | 804 | 808 | conservative cis-element |
| TaCCHC22 | CAAT-box | 885 | 889 | conservative cis-element |
| TaCCHC22 | CAAT-box | 1019 | 1023 | conservative cis-element |
| TaCCHC22 | CAAT-box | 1107 | 1112 | conservative cis-element |
| TaCCHC22 | CAAT-box | 1108 | 1112 | conservative cis-element |
| TaCCHC22 | CAAT-box | 1415 | 1419 | conservative cis-element |
| TaCCHC22 | CAAT-box | 1431 | 1435 | conservative cis-element |
| TaCCHC22 | CAAT-box | 1449 | 1454 | conservative cis-element |
| TaCCHC22 | CAAT-box | 1489 | 1494 | conservative cis-element |
| TaCCHC22 | CAAT-box | 1494 | 1499 | conservative cis-element |
| TaCCHC22 | TATA-box | 164 | 168 | conservative cis-element |
| TaCCHC22 | TATA-box | 194 | 198 | conservative cis-element |
| TaCCHC22 | TATA-box | 238 | 244 | conservative cis-element |
| TaCCHC22 | TATA-box | 239 | 244 | conservative cis-element |
| TaCCHC22 | TATA-box | 240 | 244 | conservative cis-element |
| TaCCHC22 | TATA-box | 277 | 283 | conservative cis-element |
| TaCCHC22 | TATA-box | 278 | 282 | conservative cis-element |
| TaCCHC22 | TATA-box | 451 | 455 | conservative cis-element |
| TaCCHC22 | TATA-box | 488 | 493 | conservative cis-element |
| TaCCHC22 | TATA-box | 489 | 493 | conservative cis-element |
| TaCCHC22 | TATA-box | 529 | 535 | conservative cis-element |
| TaCCHC22 | TATA-box | 530 | 534 | conservative cis-element |
| TaCCHC22 | TATA-box | 551 | 555 | conservative cis-element |
| TaCCHC22 | TATA-box | 650 | 656 | conservative cis-element |
| TaCCHC22 | TATA-box | 651 | 657 | conservative cis-element |
| TaCCHC22 | TATA-box | 653 | 657 | conservative cis-element |
| TaCCHC22 | TATA-box | 747 | 753 | conservative cis-element |
| TaCCHC22 | TATA-box | 748 | 752 | conservative cis-element |
| TaCCHC23 | GARE-motif | 495 | 502 | gibberellin responsive |
| TaCCHC23 | MBS | 837 | 843 | drought responsive |
| TaCCHC23 | LTR | 1439 | 1445 | low-temperature responsive |
| TaCCHC23 | LTR | 1479 | 1485 | low-temperature responsive |
| TaCCHC23 | ABRE | 386 | 393 | ABA responsive |
| TaCCHC23 | ABRE | 1191 | 1200 | ABA responsive |
| TaCCHC23 | ABRE | 1193 | 1199 | ABA responsive |
| TaCCHC23 | ABRE | 1194 | 1199 | ABA responsive |
| TaCCHC23 | ARE | 203 | 209 | anaerobic induction |
| TaCCHC23 | ARE | 833 | 839 | anaerobic induction |
| TaCCHC23 | TATA-box | 38 | 44 | conservative cis-element |
| TaCCHC23 | TATA-box | 40 | 44 | conservative cis-element |
| TaCCHC23 | TATA-box | 320 | 324 | conservative cis-element |
| TaCCHC23 | TATA-box | 468 | 472 | conservative cis-element |
| TaCCHC23 | TATA-box | 648 | 652 | conservative cis-element |
| TaCCHC23 | TATA-box | 1135 | 1139 | conservative cis-element |
| TaCCHC23 | CAAT-box | 4 | 8 | conservative cis-element |
| TaCCHC23 | CAAT-box | 159 | 164 | conservative cis-element |
| TaCCHC23 | CAAT-box | 160 | 164 | conservative cis-element |
| TaCCHC23 | CAAT-box | 192 | 197 | conservative cis-element |
| TaCCHC23 | CAAT-box | 206 | 211 | conservative cis-element |
| TaCCHC23 | CAAT-box | 207 | 211 | conservative cis-element |
| TaCCHC23 | CAAT-box | 215 | 220 | conservative cis-element |
| TaCCHC23 | CAAT-box | 216 | 220 | conservative cis-element |
| TaCCHC23 | CAAT-box | 287 | 292 | conservative cis-element |
| TaCCHC23 | CAAT-box | 310 | 315 | conservative cis-element |
| TaCCHC23 | CAAT-box | 357 | 361 | conservative cis-element |
| TaCCHC23 | CAAT-box | 429 | 433 | conservative cis-element |
| TaCCHC23 | CAAT-box | 453 | 458 | conservative cis-element |
| TaCCHC23 | CAAT-box | 476 | 480 | conservative cis-element |
| TaCCHC23 | CAAT-box | 517 | 521 | conservative cis-element |
| TaCCHC23 | CAAT-box | 553 | 558 | conservative cis-element |
| TaCCHC23 | CAAT-box | 609 | 614 | conservative cis-element |
| TaCCHC23 | CAAT-box | 639 | 643 | conservative cis-element |
| TaCCHC23 | CAAT-box | 726 | 730 | conservative cis-element |
| TaCCHC23 | CAAT-box | 820 | 825 | conservative cis-element |
| TaCCHC23 | CAAT-box | 839 | 846 | conservative cis-element |
| TaCCHC23 | CAAT-box | 952 | 957 | conservative cis-element |
| TaCCHC23 | CAAT-box | 953 | 957 | conservative cis-element |
| TaCCHC23 | CAAT-box | 1000 | 1004 | conservative cis-element |
| TaCCHC23 | CAAT-box | 1042 | 1046 | conservative cis-element |
| TaCCHC23 | CAAT-box | 1056 | 1060 | conservative cis-element |
| TaCCHC23 | CAAT-box | 1415 | 1420 | conservative cis-element |
| TaCCHC23 | CAAT-box | 1431 | 1435 | conservative cis-element |
| TaCCHC23 | CAAT-box | 1449 | 1454 | conservative cis-element |
| TaCCHC23 | CAAT-box | 1489 | 1494 | conservative cis-element |
| TaCCHC23 | CAAT-box | 1494 | 1499 | conservative cis-element |
| TaCCHC24 | TC-rich repeats | 610 | 619 | defense and stress responsive |
| TaCCHC24 | TGA-element | 246 | 252 | auxin responsive |
| TaCCHC24 | TGA-element | 326 | 332 | auxin responsive |
| TaCCHC24 | TGA-element | 594 | 600 | auxin responsive |
| TaCCHC24 | TGA-element | 654 | 660 | auxin responsive |
| TaCCHC24 | ABRE | 1188 | 1197 | ABA responsive |
| TaCCHC24 | ABRE | 1190 | 1196 | ABA responsive |
| TaCCHC24 | ABRE | 1191 | 1196 | ABA responsive |
| TaCCHC24 | ARE | 543 | 549 | anaerobic induction |
| TaCCHC24 | TGACG-motif | 1130 | 1135 | MeJA responsive |
| TaCCHC24 | TATC-box | 1365 | 1372 | gibberellin responsive |
| TaCCHC24 | MBS | 20 | 26 | drought responsive |
| TaCCHC24 | LTR | 546 | 552 | low-temperature responsive |
| TaCCHC24 | LTR | 570 | 576 | low-temperature responsive |
| TaCCHC24 | LTR | 1439 | 1445 | low-temperature responsive |
| TaCCHC24 | LTR | 1479 | 1485 | low-temperature responsive |
| TaCCHC24 | CGTCA-motif | 1130 | 1135 | MeJA responsive |
| TaCCHC24 | CAAT-box | 62 | 66 | conservative cis-element |
| TaCCHC24 | CAAT-box | 76 | 80 | conservative cis-element |
| TaCCHC24 | CAAT-box | 83 | 87 | conservative cis-element |
| TaCCHC24 | CAAT-box | 116 | 120 | conservative cis-element |
| TaCCHC24 | CAAT-box | 122 | 127 | conservative cis-element |
| TaCCHC24 | CAAT-box | 123 | 127 | conservative cis-element |
| TaCCHC24 | CAAT-box | 146 | 150 | conservative cis-element |
| TaCCHC24 | CAAT-box | 148 | 152 | conservative cis-element |
| TaCCHC24 | CAAT-box | 181 | 185 | conservative cis-element |
| TaCCHC24 | CAAT-box | 217 | 222 | conservative cis-element |
| TaCCHC24 | CAAT-box | 226 | 231 | conservative cis-element |
| TaCCHC24 | CAAT-box | 349 | 353 | conservative cis-element |
| TaCCHC24 | CAAT-box | 692 | 696 | conservative cis-element |
| TaCCHC24 | CAAT-box | 717 | 721 | conservative cis-element |
| TaCCHC24 | CAAT-box | 810 | 814 | conservative cis-element |
| TaCCHC24 | CAAT-box | 883 | 887 | conservative cis-element |
| TaCCHC24 | CAAT-box | 1108 | 1113 | conservative cis-element |
| TaCCHC24 | CAAT-box | 1109 | 1113 | conservative cis-element |
| TaCCHC24 | CAAT-box | 1415 | 1419 | conservative cis-element |
| TaCCHC24 | CAAT-box | 1431 | 1435 | conservative cis-element |
| TaCCHC24 | CAAT-box | 1449 | 1454 | conservative cis-element |
| TaCCHC24 | CAAT-box | 1489 | 1494 | conservative cis-element |
| TaCCHC24 | CAAT-box | 1494 | 1499 | conservative cis-element |
| TaCCHC24 | TATA-box | 109 | 113 | conservative cis-element |
| TaCCHC24 | TATA-box | 160 | 164 | conservative cis-element |
| TaCCHC24 | TATA-box | 214 | 218 | conservative cis-element |
| TaCCHC24 | TATA-box | 395 | 399 | conservative cis-element |
| TaCCHC24 | TATA-box | 431 | 436 | conservative cis-element |
| TaCCHC24 | TATA-box | 432 | 436 | conservative cis-element |
| TaCCHC24 | TATA-box | 472 | 478 | conservative cis-element |
| TaCCHC24 | TATA-box | 473 | 477 | conservative cis-element |
| TaCCHC24 | TATA-box | 494 | 498 | conservative cis-element |
| TaCCHC24 | TATA-box | 503 | 512 | conservative cis-element |
| TaCCHC24 | TATA-box | 633 | 637 | conservative cis-element |
| TaCCHC24 | TATA-box | 727 | 733 | conservative cis-element |
| TaCCHC24 | TATA-box | 728 | 733 | conservative cis-element |
| TaCCHC24 | TATA-box | 729 | 733 | conservative cis-element |
| TaCCHC25 | ARE | 931 | 937 | anaerobic induction |
| TaCCHC25 | ARE | 1263 | 1269 | anaerobic induction |
| TaCCHC25 | ABRE | 551 | 558 | ABA responsive |
| TaCCHC25 | ABRE | 819 | 824 | ABA responsive |
| TaCCHC25 | GC-motif | 768 | 774 | anoxic specific inducibility |
| TaCCHC25 | GC-motif | 907 | 913 | anoxic specific inducibility |
| TaCCHC25 | GC-motif | 1043 | 1049 | anoxic specific inducibility |
| TaCCHC25 | GC-motif | 1050 | 1056 | anoxic specific inducibility |
| TaCCHC25 | GC-motif | 1069 | 1075 | anoxic specific inducibility |
| TaCCHC25 | TCA-element | 1247 | 1256 | salicylic acid responsive |
| TaCCHC25 | CGTCA-motif | 219 | 224 | MeJA responsive |
| TaCCHC25 | CGTCA-motif | 964 | 969 | MeJA responsive |
| TaCCHC25 | CGTCA-motif | 1354 | 1359 | MeJA responsive |
| TaCCHC25 | LTR | 1489 | 1495 | low-temperature responsive |
| TaCCHC25 | TGACG-motif | 219 | 224 | MeJA responsive |
| TaCCHC25 | TGACG-motif | 964 | 969 | MeJA responsive |
| TaCCHC25 | TGACG-motif | 1354 | 1359 | MeJA responsive |
| TaCCHC25 | CAAT-box | 40 | 45 | conservative cis-element |
| TaCCHC25 | CAAT-box | 70 | 75 | conservative cis-element |
| TaCCHC25 | CAAT-box | 71 | 75 | conservative cis-element |
| TaCCHC25 | CAAT-box | 235 | 239 | conservative cis-element |
| TaCCHC25 | CAAT-box | 272 | 276 | conservative cis-element |
| TaCCHC25 | CAAT-box | 301 | 305 | conservative cis-element |
| TaCCHC25 | CAAT-box | 368 | 372 | conservative cis-element |
| TaCCHC25 | CAAT-box | 480 | 484 | conservative cis-element |
| TaCCHC25 | CAAT-box | 497 | 502 | conservative cis-element |
| TaCCHC25 | CAAT-box | 517 | 521 | conservative cis-element |
| TaCCHC25 | CAAT-box | 585 | 590 | conservative cis-element |
| TaCCHC25 | CAAT-box | 628 | 633 | conservative cis-element |
| TaCCHC25 | CAAT-box | 684 | 689 | conservative cis-element |
| TaCCHC25 | CAAT-box | 714 | 719 | conservative cis-element |
| TaCCHC25 | CAAT-box | 791 | 795 | conservative cis-element |
| TaCCHC25 | CAAT-box | 854 | 859 | conservative cis-element |
| TaCCHC25 | CAAT-box | 935 | 940 | conservative cis-element |
| TaCCHC25 | CAAT-box | 1350 | 1354 | conservative cis-element |
| TaCCHC25 | CAAT-box | 1387 | 1392 | conservative cis-element |
| TaCCHC25 | CAAT-box | 1398 | 1403 | conservative cis-element |
| TaCCHC25 | TATA-box | 284 | 288 | conservative cis-element |
| TaCCHC25 | TATA-box | 565 | 569 | conservative cis-element |
| TaCCHC25 | TATA-box | 950 | 954 | conservative cis-element |
| TaCCHC25 | TATA-box | 1335 | 1339 | conservative cis-element |
| TaCCHC26 | ABRE | 606 | 611 | ABA responsive |
| TaCCHC26 | GC-motif | 909 | 915 | anoxic specific inducibility |
| TaCCHC26 | GC-motif | 1045 | 1051 | anoxic specific inducibility |
| TaCCHC26 | GC-motif | 1052 | 1058 | anoxic specific inducibility |
| TaCCHC26 | GC-motif | 1071 | 1077 | anoxic specific inducibility |
| TaCCHC26 | ARE | 933 | 939 | anaerobic induction |
| TaCCHC26 | ARE | 1265 | 1271 | anaerobic induction |
| TaCCHC26 | ARE | 1459 | 1465 | anaerobic induction |
| TaCCHC26 | P-box | 804 | 811 | gibberellin responsive |
| TaCCHC26 | LTR | 558 | 564 | low-temperature responsive |
| TaCCHC26 | LTR | 1489 | 1495 | low-temperature responsive |
| TaCCHC26 | MBS | 370 | 376 | drought responsive |
| TaCCHC26 | MBS | 711 | 717 | drought responsive |
| TaCCHC26 | TCA-element | 1249 | 1258 | salicylic acid responsive |
| TaCCHC26 | CGTCA-motif | 211 | 216 | MeJA responsive |
| TaCCHC26 | CGTCA-motif | 826 | 831 | MeJA responsive |
| TaCCHC26 | CGTCA-motif | 966 | 971 | MeJA responsive |
| TaCCHC26 | CGTCA-motif | 1472 | 1477 | MeJA responsive |
| TaCCHC26 | TGACG-motif | 211 | 216 | MeJA responsive |
| TaCCHC26 | TGACG-motif | 826 | 831 | MeJA responsive |
| TaCCHC26 | TGACG-motif | 966 | 971 | MeJA responsive |
| TaCCHC26 | TGACG-motif | 1472 | 1477 | MeJA responsive |
| TaCCHC26 | CAAT-box | 2 | 7 | conservative cis-element |
| TaCCHC26 | CAAT-box | 13 | 18 | conservative cis-element |
| TaCCHC26 | CAAT-box | 33 | 37 | conservative cis-element |
| TaCCHC26 | CAAT-box | 94 | 99 | conservative cis-element |
| TaCCHC26 | CAAT-box | 125 | 129 | conservative cis-element |
| TaCCHC26 | CAAT-box | 138 | 143 | conservative cis-element |
| TaCCHC26 | CAAT-box | 229 | 234 | conservative cis-element |
| TaCCHC26 | CAAT-box | 240 | 245 | conservative cis-element |
| TaCCHC26 | CAAT-box | 297 | 302 | conservative cis-element |
| TaCCHC26 | CAAT-box | 298 | 302 | conservative cis-element |
| TaCCHC26 | CAAT-box | 311 | 316 | conservative cis-element |
| TaCCHC26 | CAAT-box | 346 | 351 | conservative cis-element |
| TaCCHC26 | CAAT-box | 349 | 354 | conservative cis-element |
| TaCCHC26 | CAAT-box | 395 | 400 | conservative cis-element |
| TaCCHC26 | CAAT-box | 429 | 434 | conservative cis-element |
| TaCCHC26 | CAAT-box | 443 | 448 | conservative cis-element |
| TaCCHC26 | CAAT-box | 464 | 469 | conservative cis-element |
| TaCCHC26 | CAAT-box | 480 | 484 | conservative cis-element |
| TaCCHC26 | CAAT-box | 512 | 517 | conservative cis-element |
| TaCCHC26 | CAAT-box | 517 | 522 | conservative cis-element |
| TaCCHC26 | CAAT-box | 615 | 620 | conservative cis-element |
| TaCCHC26 | CAAT-box | 645 | 650 | conservative cis-element |
| TaCCHC26 | CAAT-box | 673 | 678 | conservative cis-element |
| TaCCHC26 | CAAT-box | 702 | 707 | conservative cis-element |
| TaCCHC26 | CAAT-box | 856 | 861 | conservative cis-element |
| TaCCHC26 | CAAT-box | 937 | 942 | conservative cis-element |
| TaCCHC26 | CAAT-box | 1184 | 1189 | conservative cis-element |
| TaCCHC26 | CAAT-box | 1276 | 1281 | conservative cis-element |
| TaCCHC26 | CAAT-box | 1350 | 1354 | conservative cis-element |
| TaCCHC26 | CAAT-box | 1382 | 1386 | conservative cis-element |
| TaCCHC26 | CAAT-box | 1387 | 1392 | conservative cis-element |
| TaCCHC26 | CAAT-box | 1398 | 1403 | conservative cis-element |
| TaCCHC26 | TATA-box | 100 | 107 | conservative cis-element |
| TaCCHC26 | TATA-box | 698 | 702 | conservative cis-element |
| TaCCHC26 | TATA-box | 952 | 956 | conservative cis-element |
| TaCCHC26 | TATA-box | 1407 | 1416 | conservative cis-element |
| TaCCHC27 | TGACG-motif | 337 | 342 | MeJA responsive |
| TaCCHC27 | TGACG-motif | 828 | 833 | MeJA responsive |
| TaCCHC27 | TGACG-motif | 968 | 973 | MeJA responsive |
| TaCCHC27 | TGACG-motif | 1354 | 1359 | MeJA responsive |
| TaCCHC27 | LTR | 1489 | 1495 | low-temperature responsive |
| TaCCHC27 | CGTCA-motif | 337 | 342 | MeJA responsive |
| TaCCHC27 | CGTCA-motif | 828 | 833 | MeJA responsive |
| TaCCHC27 | CGTCA-motif | 968 | 973 | MeJA responsive |
| TaCCHC27 | CGTCA-motif | 1354 | 1359 | MeJA responsive |
| TaCCHC27 | TCA-element | 1249 | 1258 | salicylic acid responsive |
| TaCCHC27 | GC-motif | 1047 | 1053 | anoxic specific inducibility |
| TaCCHC27 | GC-motif | 1071 | 1077 | anoxic specific inducibility |
| TaCCHC27 | ABRE | 554 | 560 | ABA responsive |
| TaCCHC27 | ABRE | 555 | 560 | ABA responsive |
| TaCCHC27 | ARE | 699 | 705 | anaerobic induction |
| TaCCHC27 | ARE | 935 | 941 | anaerobic induction |
| TaCCHC27 | ARE | 1265 | 1271 | anaerobic induction |
| TaCCHC27 | TATA-box | 954 | 958 | conservative cis-element |
| TaCCHC27 | TATA-box | 1407 | 1416 | conservative cis-element |
| TaCCHC27 | CAAT-box | 3 | 7 | conservative cis-element |
| TaCCHC27 | CAAT-box | 66 | 71 | conservative cis-element |
| TaCCHC27 | CAAT-box | 73 | 77 | conservative cis-element |
| TaCCHC27 | CAAT-box | 379 | 383 | conservative cis-element |
| TaCCHC27 | CAAT-box | 431 | 435 | conservative cis-element |
| TaCCHC27 | CAAT-box | 775 | 779 | conservative cis-element |
| TaCCHC27 | CAAT-box | 858 | 863 | conservative cis-element |
| TaCCHC27 | CAAT-box | 939 | 944 | conservative cis-element |
| TaCCHC27 | CAAT-box | 1276 | 1281 | conservative cis-element |
| TaCCHC27 | CAAT-box | 1382 | 1386 | conservative cis-element |
| TaCCHC27 | CAAT-box | 1398 | 1403 | conservative cis-element |
| TaCCHC28 | LTR | 6 | 12 | low-temperature responsive |
| TaCCHC28 | LTR | 721 | 727 | low-temperature responsive |
| TaCCHC28 | ARE | 770 | 776 | anaerobic induction |
| TaCCHC28 | TGA-element | 385 | 391 | auxin responsive |
| TaCCHC28 | TGA-element | 844 | 850 | auxin responsive |
| TaCCHC28 | TATA-box | 94 | 98 | conservative cis-element |
| TaCCHC28 | TATA-box | 516 | 522 | conservative cis-element |
| TaCCHC28 | TATA-box | 517 | 523 | conservative cis-element |
| TaCCHC28 | TATA-box | 518 | 522 | conservative cis-element |
| TaCCHC28 | TATA-box | 1008 | 1012 | conservative cis-element |
| TaCCHC28 | TATA-box | 1409 | 1415 | conservative cis-element |
| TaCCHC28 | TATA-box | 1410 | 1414 | conservative cis-element |
| TaCCHC28 | CAAT-box | 17 | 21 | conservative cis-element |
| TaCCHC28 | CAAT-box | 680 | 684 | conservative cis-element |
| TaCCHC28 | CAAT-box | 1087 | 1091 | conservative cis-element |
| TaCCHC28 | CAAT-box | 1167 | 1172 | conservative cis-element |
| TaCCHC28 | CAAT-box | 1168 | 1172 | conservative cis-element |
| TaCCHC28 | CAAT-box | 1290 | 1294 | conservative cis-element |
| TaCCHC28 | CAAT-box | 1295 | 1300 | conservative cis-element |
| TaCCHC28 | CAAT-box | 1298 | 1302 | conservative cis-element |
| TaCCHC28 | CAAT-box | 1395 | 1400 | conservative cis-element |
| TaCCHC28 | CAAT-box | 1422 | 1426 | conservative cis-element |
| TaCCHC28 | CAAT-box | 1451 | 1455 | conservative cis-element |
| TaCCHC29 | TGACG-motif | 41 | 46 | MeJA responsive |
| TaCCHC29 | TGACG-motif | 476 | 481 | MeJA responsive |
| TaCCHC29 | TGACG-motif | 563 | 568 | MeJA responsive |
| TaCCHC29 | TGACG-motif | 586 | 591 | MeJA responsive |
| TaCCHC29 | TGACG-motif | 1110 | 1115 | MeJA responsive |
| TaCCHC29 | TGACG-motif | 1117 | 1122 | MeJA responsive |
| TaCCHC29 | CGTCA-motif | 41 | 46 | MeJA responsive |
| TaCCHC29 | CGTCA-motif | 476 | 481 | MeJA responsive |
| TaCCHC29 | CGTCA-motif | 563 | 568 | MeJA responsive |
| TaCCHC29 | CGTCA-motif | 586 | 591 | MeJA responsive |
| TaCCHC29 | CGTCA-motif | 1110 | 1115 | MeJA responsive |
| TaCCHC29 | CGTCA-motif | 1117 | 1122 | MeJA responsive |
| TaCCHC29 | LTR | 235 | 241 | low-temperature responsive |
| TaCCHC29 | LTR | 271 | 277 | low-temperature responsive |
| TaCCHC29 | LTR | 306 | 312 | low-temperature responsive |
| TaCCHC29 | LTR | 1140 | 1146 | low-temperature responsive |
| TaCCHC29 | MBS | 574 | 580 | drought responsive |
| TaCCHC29 | MBS | 597 | 603 | drought responsive |
| TaCCHC29 | MBS | 704 | 710 | drought responsive |
| TaCCHC29 | TGA-element | 100 | 106 | auxin responsive |
| TaCCHC29 | ARE | 911 | 917 | anaerobic induction |
| TaCCHC29 | ARE | 1003 | 1009 | anaerobic induction |
| TaCCHC29 | ABRE | 177 | 184 | ABA responsive |
| TaCCHC29 | ABRE | 1085 | 1094 | ABA responsive |
| TaCCHC29 | ABRE | 1087 | 1093 | ABA responsive |
| TaCCHC29 | ABRE | 1088 | 1093 | ABA responsive |
| TaCCHC29 | ABRE | 1209 | 1214 | ABA responsive |
| TaCCHC29 | GC-motif | 1456 | 1462 | anoxic specific inducibility |
| TaCCHC29 | TATA-box | 175 | 179 | conservative cis-element |
| TaCCHC29 | TATA-box | 1053 | 1059 | conservative cis-element |
| TaCCHC29 | TATA-box | 1054 | 1058 | conservative cis-element |
| TaCCHC29 | TATA-box | 1282 | 1288 | conservative cis-element |
| TaCCHC29 | TATA-box | 1283 | 1287 | conservative cis-element |
| TaCCHC29 | CAAT-box | 21 | 25 | conservative cis-element |
| TaCCHC29 | CAAT-box | 58 | 62 | conservative cis-element |
| TaCCHC29 | CAAT-box | 138 | 142 | conservative cis-element |
| TaCCHC29 | CAAT-box | 286 | 291 | conservative cis-element |
| TaCCHC29 | CAAT-box | 287 | 291 | conservative cis-element |
| TaCCHC29 | CAAT-box | 401 | 406 | conservative cis-element |
| TaCCHC29 | CAAT-box | 803 | 807 | conservative cis-element |
| TaCCHC29 | CAAT-box | 1229 | 1234 | conservative cis-element |
| TaCCHC30 | GC-motif | 1272 | 1278 | anoxic specific inducibility |
| TaCCHC30 | GC-motif | 1454 | 1460 | anoxic specific inducibility |
| TaCCHC30 | ABRE | 672 | 677 | ABA responsive |
| TaCCHC30 | ABRE | 924 | 929 | ABA responsive |
| TaCCHC30 | ABRE | 1163 | 1170 | ABA responsive |
| TaCCHC30 | TGA-element | 377 | 383 | auxin responsive |
| TaCCHC30 | CGTCA-motif | 163 | 168 | MeJA responsive |
| TaCCHC30 | CGTCA-motif | 363 | 368 | MeJA responsive |
| TaCCHC30 | CGTCA-motif | 674 | 679 | MeJA responsive |
| TaCCHC30 | MBS | 1116 | 1122 | drought responsive |
| TaCCHC30 | LTR | 1275 | 1281 | low-temperature responsive |
| TaCCHC30 | AuxRR-core | 633 | 640 | auxin responsive |
| TaCCHC30 | TGACG-motif | 163 | 168 | MeJA responsive |
| TaCCHC30 | TGACG-motif | 363 | 368 | MeJA responsive |
| TaCCHC30 | TGACG-motif | 674 | 679 | MeJA responsive |
| TaCCHC30 | CAAT-box | 29 | 33 | conservative cis-element |
| TaCCHC30 | CAAT-box | 89 | 93 | conservative cis-element |
| TaCCHC30 | CAAT-box | 139 | 144 | conservative cis-element |
| TaCCHC30 | CAAT-box | 220 | 225 | conservative cis-element |
| TaCCHC30 | CAAT-box | 221 | 225 | conservative cis-element |
| TaCCHC30 | CAAT-box | 254 | 258 | conservative cis-element |
| TaCCHC30 | CAAT-box | 366 | 370 | conservative cis-element |
| TaCCHC30 | CAAT-box | 390 | 395 | conservative cis-element |
| TaCCHC30 | CAAT-box | 397 | 402 | conservative cis-element |
| TaCCHC30 | CAAT-box | 429 | 433 | conservative cis-element |
| TaCCHC30 | CAAT-box | 468 | 473 | conservative cis-element |
| TaCCHC30 | CAAT-box | 499 | 503 | conservative cis-element |
| TaCCHC30 | CAAT-box | 706 | 710 | conservative cis-element |
| TaCCHC30 | CAAT-box | 1113 | 1120 | conservative cis-element |
| TaCCHC30 | TATA-box | 647 | 651 | conservative cis-element |
| TaCCHC30 | TATA-box | 1297 | 1303 | conservative cis-element |
| TaCCHC30 | TATA-box | 1298 | 1302 | conservative cis-element |
| TaCCHC31 | TGA-element | 157 | 163 | auxin responsive |
| TaCCHC31 | ERE | 795 | 803 | ethylene responsive |
| TaCCHC31 | ERE | 810 | 818 | ethylene responsive |
| TaCCHC31 | ERE | 827 | 835 | ethylene responsive |
| TaCCHC31 | ERE | 842 | 850 | ethylene responsive |
| TaCCHC31 | AuxRR-core | 258 | 265 | auxin responsive |
| TaCCHC31 | LTR | 411 | 417 | low-temperature responsive |
| TaCCHC31 | CAAT-box | 139 | 144 | conservative cis-element |
| TaCCHC31 | CAAT-box | 140 | 144 | conservative cis-element |
| TaCCHC31 | CAAT-box | 219 | 224 | conservative cis-element |
| TaCCHC31 | CAAT-box | 263 | 268 | conservative cis-element |
| TaCCHC31 | CAAT-box | 264 | 268 | conservative cis-element |
| TaCCHC31 | CAAT-box | 266 | 270 | conservative cis-element |
| TaCCHC31 | CAAT-box | 429 | 434 | conservative cis-element |
| TaCCHC31 | CAAT-box | 539 | 543 | conservative cis-element |
| TaCCHC31 | CAAT-box | 547 | 551 | conservative cis-element |
| TaCCHC31 | CAAT-box | 607 | 611 | conservative cis-element |
| TaCCHC31 | CAAT-box | 619 | 624 | conservative cis-element |
| TaCCHC31 | CAAT-box | 620 | 624 | conservative cis-element |
| TaCCHC31 | CAAT-box | 633 | 637 | conservative cis-element |
| TaCCHC31 | CAAT-box | 657 | 661 | conservative cis-element |
| TaCCHC31 | CAAT-box | 667 | 672 | conservative cis-element |
| TaCCHC31 | CAAT-box | 720 | 724 | conservative cis-element |
| TaCCHC31 | CAAT-box | 722 | 726 | conservative cis-element |
| TaCCHC31 | CAAT-box | 803 | 807 | conservative cis-element |
| TaCCHC31 | CAAT-box | 838 | 842 | conservative cis-element |
| TaCCHC31 | CAAT-box | 903 | 908 | conservative cis-element |
| TaCCHC31 | CAAT-box | 942 | 947 | conservative cis-element |
| TaCCHC31 | CAAT-box | 1122 | 1126 | conservative cis-element |
| TaCCHC31 | CAAT-box | 1138 | 1143 | conservative cis-element |
| TaCCHC31 | CAAT-box | 1145 | 1149 | conservative cis-element |
| TaCCHC31 | CAAT-box | 1170 | 1174 | conservative cis-element |
| TaCCHC31 | CAAT-box | 1197 | 1201 | conservative cis-element |
| TaCCHC31 | CAAT-box | 1223 | 1232 | conservative cis-element |
| TaCCHC31 | CAAT-box | 1225 | 1230 | conservative cis-element |
| TaCCHC31 | CAAT-box | 1230 | 1234 | conservative cis-element |
| TaCCHC31 | CAAT-box | 1316 | 1320 | conservative cis-element |
| TaCCHC31 | CAAT-box | 1341 | 1348 | conservative cis-element |
| TaCCHC31 | TATA-box | 145 | 152 | conservative cis-element |
| TaCCHC31 | TATA-box | 146 | 152 | conservative cis-element |
| TaCCHC31 | TATA-box | 147 | 152 | conservative cis-element |
| TaCCHC31 | TATA-box | 148 | 152 | conservative cis-element |
| TaCCHC31 | TATA-box | 360 | 366 | conservative cis-element |
| TaCCHC31 | TATA-box | 361 | 365 | conservative cis-element |
| TaCCHC31 | TATA-box | 589 | 593 | conservative cis-element |
| TaCCHC31 | TATA-box | 825 | 833 | conservative cis-element |
| TaCCHC32 | ARE | 107 | 113 | anaerobic induction |
| TaCCHC32 | ARE | 1459 | 1465 | anaerobic induction |
| TaCCHC32 | ABRE | 313 | 318 | ABA responsive |
| TaCCHC32 | ABRE | 380 | 389 | ABA responsive |
| TaCCHC32 | ABRE | 453 | 458 | ABA responsive |
| TaCCHC32 | ABRE | 731 | 736 | ABA responsive |
| TaCCHC32 | P-box | 633 | 640 | gibberellin responsive |
| TaCCHC32 | ERE | 1166 | 1174 | ethylene responsive |
| TaCCHC32 | TATA-box | 19 | 25 | conservative cis-element |
| TaCCHC32 | TATA-box | 21 | 25 | conservative cis-element |
| TaCCHC32 | TATA-box | 238 | 242 | conservative cis-element |
| TaCCHC32 | TATA-box | 528 | 533 | conservative cis-element |
| TaCCHC32 | TATA-box | 529 | 533 | conservative cis-element |
| TaCCHC32 | TATA-box | 777 | 783 | conservative cis-element |
| TaCCHC32 | TATA-box | 779 | 783 | conservative cis-element |
| TaCCHC32 | TATA-box | 954 | 962 | conservative cis-element |
| TaCCHC32 | TATA-box | 980 | 984 | conservative cis-element |
| TaCCHC32 | TATA-box | 1060 | 1068 | conservative cis-element |
| TaCCHC32 | TATA-box | 1140 | 1147 | conservative cis-element |
| TaCCHC32 | TATA-box | 1147 | 1153 | conservative cis-element |
| TaCCHC32 | TATA-box | 1148 | 1154 | conservative cis-element |
| TaCCHC32 | TATA-box | 1150 | 1154 | conservative cis-element |
| TaCCHC32 | TATA-box | 1155 | 1161 | conservative cis-element |
| TaCCHC32 | TATA-box | 1156 | 1162 | conservative cis-element |
| TaCCHC32 | TATA-box | 1158 | 1162 | conservative cis-element |
| TaCCHC32 | TATA-box | 1175 | 1179 | conservative cis-element |
| TaCCHC32 | TATA-box | 1187 | 1194 | conservative cis-element |
| TaCCHC32 | CAAT-box | 24 | 28 | conservative cis-element |
| TaCCHC32 | CAAT-box | 119 | 123 | conservative cis-element |
| TaCCHC32 | CAAT-box | 205 | 209 | conservative cis-element |
| TaCCHC32 | CAAT-box | 479 | 484 | conservative cis-element |
| TaCCHC32 | CAAT-box | 557 | 561 | conservative cis-element |
| TaCCHC32 | CAAT-box | 741 | 745 | conservative cis-element |
| TaCCHC32 | CAAT-box | 769 | 774 | conservative cis-element |
| TaCCHC32 | CAAT-box | 811 | 816 | conservative cis-element |
| TaCCHC32 | CAAT-box | 823 | 828 | conservative cis-element |
| TaCCHC32 | CAAT-box | 872 | 877 | conservative cis-element |
| TaCCHC32 | CAAT-box | 913 | 918 | conservative cis-element |
| TaCCHC32 | CAAT-box | 941 | 945 | conservative cis-element |
| TaCCHC32 | CAAT-box | 1011 | 1015 | conservative cis-element |
| TaCCHC32 | CAAT-box | 1042 | 1046 | conservative cis-element |
| TaCCHC32 | CAAT-box | 1074 | 1079 | conservative cis-element |
| TaCCHC32 | CAAT-box | 1077 | 1081 | conservative cis-element |
| TaCCHC32 | CAAT-box | 1118 | 1123 | conservative cis-element |
| TaCCHC32 | CAAT-box | 1450 | 1455 | conservative cis-element |
| TaCCHC33 | LTR | 892 | 898 | low-temperature responsive |
| TaCCHC33 | ABRE | 12 | 21 | ABA responsive |
| TaCCHC33 | ABRE | 1232 | 1241 | ABA responsive |
| TaCCHC33 | ARE | 321 | 327 | anaerobic induction |
| TaCCHC33 | ARE | 329 | 335 | anaerobic induction |
| TaCCHC33 | ARE | 474 | 480 | anaerobic induction |
| TaCCHC33 | ARE | 1458 | 1464 | anaerobic induction |
| TaCCHC33 | ERE | 651 | 659 | ethylene responsive |
| TaCCHC33 | ERE | 937 | 945 | ethylene responsive |
| TaCCHC33 | TATA-box | 7 | 11 | conservative cis-element |
| TaCCHC33 | TATA-box | 900 | 909 | conservative cis-element |
| TaCCHC33 | TATA-box | 901 | 908 | conservative cis-element |
| TaCCHC33 | TATA-box | 902 | 908 | conservative cis-element |
| TaCCHC33 | TATA-box | 903 | 908 | conservative cis-element |
| TaCCHC33 | TATA-box | 904 | 908 | conservative cis-element |
| TaCCHC33 | TATA-box | 922 | 929 | conservative cis-element |
| TaCCHC33 | TATA-box | 923 | 929 | conservative cis-element |
| TaCCHC33 | TATA-box | 924 | 929 | conservative cis-element |
| TaCCHC33 | TATA-box | 925 | 929 | conservative cis-element |
| TaCCHC33 | TATA-box | 958 | 966 | conservative cis-element |
| TaCCHC33 | TATA-box | 959 | 966 | conservative cis-element |
| TaCCHC33 | TATA-box | 960 | 966 | conservative cis-element |
| TaCCHC33 | TATA-box | 961 | 966 | conservative cis-element |
| TaCCHC33 | TATA-box | 962 | 966 | conservative cis-element |
| TaCCHC33 | TATA-box | 978 | 982 | conservative cis-element |
| TaCCHC33 | CAAT-box | 40 | 45 | conservative cis-element |
| TaCCHC33 | CAAT-box | 198 | 202 | conservative cis-element |
| TaCCHC33 | CAAT-box | 222 | 226 | conservative cis-element |
| TaCCHC33 | CAAT-box | 242 | 250 | conservative cis-element |
| TaCCHC33 | CAAT-box | 243 | 248 | conservative cis-element |
| TaCCHC33 | CAAT-box | 244 | 248 | conservative cis-element |
| TaCCHC33 | CAAT-box | 339 | 344 | conservative cis-element |
| TaCCHC33 | CAAT-box | 384 | 388 | conservative cis-element |
| TaCCHC33 | CAAT-box | 407 | 411 | conservative cis-element |
| TaCCHC33 | CAAT-box | 425 | 430 | conservative cis-element |
| TaCCHC33 | CAAT-box | 442 | 447 | conservative cis-element |
| TaCCHC33 | CAAT-box | 460 | 464 | conservative cis-element |
| TaCCHC33 | CAAT-box | 497 | 501 | conservative cis-element |
| TaCCHC33 | CAAT-box | 532 | 537 | conservative cis-element |
| TaCCHC33 | CAAT-box | 535 | 540 | conservative cis-element |
| TaCCHC33 | CAAT-box | 553 | 558 | conservative cis-element |
| TaCCHC33 | CAAT-box | 556 | 561 | conservative cis-element |
| TaCCHC33 | CAAT-box | 580 | 584 | conservative cis-element |
| TaCCHC33 | CAAT-box | 619 | 624 | conservative cis-element |
| TaCCHC33 | CAAT-box | 639 | 643 | conservative cis-element |
| TaCCHC33 | CAAT-box | 695 | 700 | conservative cis-element |
| TaCCHC33 | CAAT-box | 698 | 703 | conservative cis-element |
| TaCCHC33 | CAAT-box | 722 | 726 | conservative cis-element |
| TaCCHC33 | CAAT-box | 768 | 773 | conservative cis-element |
| TaCCHC33 | CAAT-box | 877 | 882 | conservative cis-element |
| TaCCHC33 | CAAT-box | 1008 | 1013 | conservative cis-element |
| TaCCHC33 | CAAT-box | 1095 | 1100 | conservative cis-element |
| TaCCHC33 | CAAT-box | 1096 | 1100 | conservative cis-element |
| TaCCHC33 | CAAT-box | 1135 | 1140 | conservative cis-element |
| TaCCHC33 | CAAT-box | 1198 | 1203 | conservative cis-element |
| TaCCHC33 | CAAT-box | 1449 | 1454 | conservative cis-element |
| TaCCHC34 | TGACG-motif | 602 | 607 | MeJA responsive |
| TaCCHC34 | LTR | 703 | 709 | low-temperature responsive |
| TaCCHC34 | CGTCA-motif | 602 | 607 | MeJA responsive |
| TaCCHC34 | TGA-element | 375 | 381 | auxin responsive |
| TaCCHC34 | ABRE | 1035 | 1040 | ABA responsive |
| TaCCHC34 | ARE | 180 | 186 | anaerobic induction |
| TaCCHC34 | ARE | 765 | 771 | anaerobic induction |
| TaCCHC34 | TATA-box | 501 | 507 | conservative cis-element |
| TaCCHC34 | TATA-box | 502 | 508 | conservative cis-element |
| TaCCHC34 | TATA-box | 503 | 507 | conservative cis-element |
| TaCCHC34 | TATA-box | 753 | 757 | conservative cis-element |
| TaCCHC34 | TATA-box | 1411 | 1417 | conservative cis-element |
| TaCCHC34 | TATA-box | 1412 | 1416 | conservative cis-element |
| TaCCHC34 | CAAT-box | 36 | 41 | conservative cis-element |
| TaCCHC34 | CAAT-box | 249 | 254 | conservative cis-element |
| TaCCHC34 | CAAT-box | 565 | 575 | conservative cis-element |
| TaCCHC34 | CAAT-box | 569 | 574 | conservative cis-element |
| TaCCHC34 | CAAT-box | 673 | 677 | conservative cis-element |
| TaCCHC34 | CAAT-box | 804 | 808 | conservative cis-element |
| TaCCHC34 | CAAT-box | 818 | 823 | conservative cis-element |
| TaCCHC34 | CAAT-box | 819 | 823 | conservative cis-element |
| TaCCHC34 | CAAT-box | 980 | 984 | conservative cis-element |
| TaCCHC34 | CAAT-box | 1084 | 1088 | conservative cis-element |
| TaCCHC34 | CAAT-box | 1165 | 1170 | conservative cis-element |
| TaCCHC34 | CAAT-box | 1166 | 1170 | conservative cis-element |
| TaCCHC34 | CAAT-box | 1289 | 1293 | conservative cis-element |
| TaCCHC34 | CAAT-box | 1294 | 1299 | conservative cis-element |
| TaCCHC34 | CAAT-box | 1297 | 1301 | conservative cis-element |
| TaCCHC34 | CAAT-box | 1372 | 1377 | conservative cis-element |
| TaCCHC34 | CAAT-box | 1397 | 1402 | conservative cis-element |
| TaCCHC34 | CAAT-box | 1422 | 1426 | conservative cis-element |
| TaCCHC35 | WUN-motif | 118 | 127 | wound responsive |
| TaCCHC35 | WUN-motif | 1444 | 1452 | wound responsive |
| TaCCHC35 | P-box | 607 | 614 | gibberellin responsive |
| TaCCHC35 | ABRE | 80 | 85 | ABA responsive |
| TaCCHC35 | ABRE | 709 | 714 | ABA responsive |
| TaCCHC35 | ABRE | 913 | 918 | ABA responsive |
| TaCCHC35 | ABRE | 951 | 956 | ABA responsive |
| TaCCHC35 | ABRE | 1325 | 1330 | ABA responsive |
| TaCCHC35 | GC-motif | 968 | 974 | anoxic specific inducibility |
| TaCCHC35 | GC-motif | 1337 | 1343 | anoxic specific inducibility |
| TaCCHC35 | TGACG-motif | 697 | 702 | MeJA responsive |
| TaCCHC35 | TGACG-motif | 846 | 851 | MeJA responsive |
| TaCCHC35 | MBS | 171 | 177 | drought responsive |
| TaCCHC35 | MBS | 1476 | 1482 | drought responsive |
| TaCCHC35 | CGTCA-motif | 697 | 702 | MeJA responsive |
| TaCCHC35 | CGTCA-motif | 846 | 851 | MeJA responsive |
| TaCCHC35 | CAAT-box | 14 | 19 | conservative cis-element |
| TaCCHC35 | CAAT-box | 20 | 25 | conservative cis-element |
| TaCCHC35 | CAAT-box | 133 | 137 | conservative cis-element |
| TaCCHC35 | CAAT-box | 145 | 150 | conservative cis-element |
| TaCCHC35 | CAAT-box | 146 | 150 | conservative cis-element |
| TaCCHC35 | CAAT-box | 181 | 186 | conservative cis-element |
| TaCCHC35 | CAAT-box | 235 | 239 | conservative cis-element |
| TaCCHC35 | CAAT-box | 237 | 241 | conservative cis-element |
| TaCCHC35 | CAAT-box | 320 | 324 | conservative cis-element |
| TaCCHC35 | CAAT-box | 412 | 417 | conservative cis-element |
| TaCCHC35 | CAAT-box | 417 | 421 | conservative cis-element |
| TaCCHC35 | CAAT-box | 501 | 505 | conservative cis-element |
| TaCCHC35 | CAAT-box | 984 | 989 | conservative cis-element |
| TaCCHC35 | CAAT-box | 1000 | 1005 | conservative cis-element |
| TaCCHC35 | CAAT-box | 1036 | 1041 | conservative cis-element |
| TaCCHC35 | CAAT-box | 1108 | 1112 | conservative cis-element |
| TaCCHC35 | CAAT-box | 1185 | 1190 | conservative cis-element |
| TaCCHC35 | CAAT-box | 1190 | 1194 | conservative cis-element |
| TaCCHC35 | CAAT-box | 1223 | 1227 | conservative cis-element |
| TaCCHC35 | CAAT-box | 1227 | 1232 | conservative cis-element |
| TaCCHC35 | CAAT-box | 1230 | 1235 | conservative cis-element |
| TaCCHC35 | CAAT-box | 1251 | 1256 | conservative cis-element |
| TaCCHC35 | CAAT-box | 1382 | 1387 | conservative cis-element |
| TaCCHC35 | CAAT-box | 1443 | 1448 | conservative cis-element |
| TaCCHC35 | TATA-box | 74 | 80 | conservative cis-element |
| TaCCHC35 | TATA-box | 75 | 79 | conservative cis-element |
| TaCCHC35 | TATA-box | 84 | 93 | conservative cis-element |
| TaCCHC35 | TATA-box | 85 | 92 | conservative cis-element |
| TaCCHC35 | TATA-box | 95 | 99 | conservative cis-element |
| TaCCHC35 | TATA-box | 409 | 413 | conservative cis-element |
| TaCCHC35 | TATA-box | 942 | 951 | conservative cis-element |
| TaCCHC35 | TATA-box | 944 | 950 | conservative cis-element |
| TaCCHC35 | TATA-box | 945 | 952 | conservative cis-element |
| TaCCHC35 | TATA-box | 946 | 952 | conservative cis-element |
| TaCCHC35 | TATA-box | 948 | 952 | conservative cis-element |
| TaCCHC36 | ARE | 68 | 74 | anaerobic induction |
| TaCCHC36 | ARE | 251 | 257 | anaerobic induction |
| TaCCHC36 | ABRE | 1171 | 1176 | ABA responsive |
| TaCCHC36 | GC-motif | 503 | 509 | anoxic specific inducibility |
| TaCCHC36 | GC-motif | 626 | 632 | anoxic specific inducibility |
| TaCCHC36 | TC-rich repeats | 70 | 79 | defense and stress responsive |
| TaCCHC36 | TGA-element | 351 | 357 | auxin responsive |
| TaCCHC36 | CGTCA-motif | 398 | 403 | MeJA responsive |
| TaCCHC36 | LTR | 79 | 85 | low-temperature responsive |
| TaCCHC36 | LTR | 683 | 689 | low-temperature responsive |
| TaCCHC36 | GARE-motif | 1230 | 1237 | gibberellin responsive |
| TaCCHC36 | GARE-motif | 1468 | 1475 | gibberellin responsive |
| TaCCHC36 | TGACG-motif | 398 | 403 | MeJA responsive |
| TaCCHC36 | CAAT-box | 91 | 95 | conservative cis-element |
| TaCCHC36 | CAAT-box | 739 | 744 | conservative cis-element |
| TaCCHC36 | CAAT-box | 770 | 775 | conservative cis-element |
| TaCCHC36 | CAAT-box | 975 | 979 | conservative cis-element |
| TaCCHC36 | CAAT-box | 1016 | 1020 | conservative cis-element |
| TaCCHC36 | CAAT-box | 1059 | 1063 | conservative cis-element |
| TaCCHC36 | CAAT-box | 1090 | 1094 | conservative cis-element |
| TaCCHC36 | CAAT-box | 1262 | 1266 | conservative cis-element |
| TaCCHC36 | CAAT-box | 1275 | 1279 | conservative cis-element |
| TaCCHC36 | CAAT-box | 1280 | 1285 | conservative cis-element |
| TaCCHC36 | CAAT-box | 1283 | 1287 | conservative cis-element |
| TaCCHC36 | CAAT-box | 1396 | 1401 | conservative cis-element |
| TaCCHC36 | CAAT-box | 1421 | 1425 | conservative cis-element |
| TaCCHC36 | TATA-box | 130 | 139 | conservative cis-element |
| TaCCHC36 | TATA-box | 492 | 498 | conservative cis-element |
| TaCCHC36 | TATA-box | 493 | 497 | conservative cis-element |
| TaCCHC36 | TATA-box | 952 | 961 | conservative cis-element |
| TaCCHC36 | TATA-box | 1018 | 1024 | conservative cis-element |
| TaCCHC36 | TATA-box | 1019 | 1023 | conservative cis-element |
| TaCCHC36 | TATA-box | 1410 | 1416 | conservative cis-element |
| TaCCHC36 | TATA-box | 1411 | 1415 | conservative cis-element |
| TaCCHC36 | TATA-box | 1484 | 1490 | conservative cis-element |
| TaCCHC36 | TATA-box | 1485 | 1490 | conservative cis-element |
| TaCCHC36 | TATA-box | 1486 | 1490 | conservative cis-element |
| TaCCHC37 | LTR | 1487 | 1493 | low-temperature responsive |
| TaCCHC37 | CGTCA-motif | 528 | 533 | MeJA responsive |
| TaCCHC37 | CGTCA-motif | 1246 | 1251 | MeJA responsive |
| TaCCHC37 | TGACG-motif | 528 | 533 | MeJA responsive |
| TaCCHC37 | TGACG-motif | 1246 | 1251 | MeJA responsive |
| TaCCHC37 | ABRE | 1222 | 1228 | ABA responsive |
| TaCCHC37 | ABRE | 1223 | 1228 | ABA responsive |
| TaCCHC37 | ABRE | 1244 | 1249 | ABA responsive |
| TaCCHC37 | ABRE | 1268 | 1273 | ABA responsive |
| TaCCHC37 | GC-motif | 620 | 626 | anoxic specific inducibility |
| TaCCHC37 | GC-motif | 1238 | 1244 | anoxic specific inducibility |
| TaCCHC37 | GC-motif | 1256 | 1262 | anoxic specific inducibility |
| TaCCHC37 | GC-motif | 1304 | 1310 | anoxic specific inducibility |
| TaCCHC37 | GC-motif | 1490 | 1496 | anoxic specific inducibility |
| TaCCHC37 | ARE | 1179 | 1185 | anaerobic induction |
| TaCCHC37 | P-box | 306 | 313 | gibberellin responsive |
| TaCCHC37 | TATA-box | 352 | 356 | conservative cis-element |
| TaCCHC37 | TATA-box | 414 | 418 | conservative cis-element |
| TaCCHC37 | TATA-box | 701 | 707 | conservative cis-element |
| TaCCHC37 | TATA-box | 702 | 706 | conservative cis-element |
| TaCCHC37 | TATA-box | 708 | 712 | conservative cis-element |
| TaCCHC37 | TATA-box | 797 | 801 | conservative cis-element |
| TaCCHC37 | TATA-box | 912 | 918 | conservative cis-element |
| TaCCHC37 | TATA-box | 913 | 918 | conservative cis-element |
| TaCCHC37 | TATA-box | 914 | 918 | conservative cis-element |
| TaCCHC37 | TATA-box | 955 | 964 | conservative cis-element |
| TaCCHC37 | TATA-box | 956 | 962 | conservative cis-element |
| TaCCHC37 | TATA-box | 957 | 964 | conservative cis-element |
| TaCCHC37 | TATA-box | 958 | 964 | conservative cis-element |
| TaCCHC37 | TATA-box | 960 | 964 | conservative cis-element |
| TaCCHC37 | TATA-box | 962 | 969 | conservative cis-element |
| TaCCHC37 | TATA-box | 1076 | 1081 | conservative cis-element |
| TaCCHC37 | TATA-box | 1077 | 1081 | conservative cis-element |
| TaCCHC37 | TATA-box | 1362 | 1366 | conservative cis-element |
| TaCCHC37 | TATA-box | 1383 | 1389 | conservative cis-element |
| TaCCHC37 | TATA-box | 1384 | 1390 | conservative cis-element |
| TaCCHC37 | TATA-box | 1385 | 1389 | conservative cis-element |
| TaCCHC37 | CAAT-box | 31 | 36 | conservative cis-element |
| TaCCHC37 | CAAT-box | 72 | 76 | conservative cis-element |
| TaCCHC37 | CAAT-box | 142 | 146 | conservative cis-element |
| TaCCHC37 | CAAT-box | 235 | 239 | conservative cis-element |
| TaCCHC37 | CAAT-box | 289 | 294 | conservative cis-element |
| TaCCHC37 | CAAT-box | 340 | 344 | conservative cis-element |
| TaCCHC37 | CAAT-box | 421 | 425 | conservative cis-element |
| TaCCHC37 | CAAT-box | 498 | 503 | conservative cis-element |
| TaCCHC37 | CAAT-box | 499 | 503 | conservative cis-element |
| TaCCHC37 | CAAT-box | 524 | 529 | conservative cis-element |
| TaCCHC37 | CAAT-box | 567 | 572 | conservative cis-element |
| TaCCHC37 | CAAT-box | 593 | 598 | conservative cis-element |
| TaCCHC37 | CAAT-box | 594 | 598 | conservative cis-element |
| TaCCHC37 | CAAT-box | 762 | 767 | conservative cis-element |
| TaCCHC37 | CAAT-box | 854 | 858 | conservative cis-element |
| TaCCHC37 | CAAT-box | 859 | 863 | conservative cis-element |
| TaCCHC37 | CAAT-box | 866 | 870 | conservative cis-element |
| TaCCHC37 | CAAT-box | 868 | 873 | conservative cis-element |
| TaCCHC37 | CAAT-box | 888 | 892 | conservative cis-element |
| TaCCHC37 | CAAT-box | 939 | 944 | conservative cis-element |
| TaCCHC37 | CAAT-box | 942 | 946 | conservative cis-element |
| TaCCHC37 | CAAT-box | 977 | 982 | conservative cis-element |
| TaCCHC37 | CAAT-box | 1089 | 1094 | conservative cis-element |
| TaCCHC37 | CAAT-box | 1104 | 1108 | conservative cis-element |
| TaCCHC38 | P-box | 926 | 933 | gibberellin responsive |
| TaCCHC38 | ARE | 27 | 33 | anaerobic induction |
| TaCCHC38 | ABRE | 149 | 154 | ABA responsive |
| TaCCHC38 | ABRE | 1308 | 1313 | ABA responsive |
| TaCCHC38 | TGACG-motif | 78 | 83 | MeJA responsive |
| TaCCHC38 | TGACG-motif | 219 | 224 | MeJA responsive |
| TaCCHC38 | TGACG-motif | 448 | 453 | MeJA responsive |
| TaCCHC38 | TGACG-motif | 1128 | 1133 | MeJA responsive |
| TaCCHC38 | TGACG-motif | 1440 | 1445 | MeJA responsive |
| TaCCHC38 | CGTCA-motif | 78 | 83 | MeJA responsive |
| TaCCHC38 | CGTCA-motif | 219 | 224 | MeJA responsive |
| TaCCHC38 | CGTCA-motif | 448 | 453 | MeJA responsive |
| TaCCHC38 | CGTCA-motif | 1128 | 1133 | MeJA responsive |
| TaCCHC38 | CGTCA-motif | 1440 | 1445 | MeJA responsive |
| TaCCHC38 | LTR | 1363 | 1369 | low-temperature responsive |
| TaCCHC38 | CAAT-box | 7 | 12 | conservative cis-element |
| TaCCHC38 | CAAT-box | 30 | 35 | conservative cis-element |
| TaCCHC38 | CAAT-box | 31 | 35 | conservative cis-element |
| TaCCHC38 | CAAT-box | 87 | 91 | conservative cis-element |
| TaCCHC38 | CAAT-box | 129 | 133 | conservative cis-element |
| TaCCHC38 | CAAT-box | 131 | 135 | conservative cis-element |
| TaCCHC38 | CAAT-box | 200 | 205 | conservative cis-element |
| TaCCHC38 | CAAT-box | 201 | 205 | conservative cis-element |
| TaCCHC38 | CAAT-box | 214 | 218 | conservative cis-element |
| TaCCHC38 | CAAT-box | 223 | 227 | conservative cis-element |
| TaCCHC38 | CAAT-box | 241 | 245 | conservative cis-element |
| TaCCHC38 | CAAT-box | 273 | 277 | conservative cis-element |
| TaCCHC38 | CAAT-box | 415 | 420 | conservative cis-element |
| TaCCHC38 | CAAT-box | 416 | 420 | conservative cis-element |
| TaCCHC38 | CAAT-box | 617 | 622 | conservative cis-element |
| TaCCHC38 | CAAT-box | 694 | 698 | conservative cis-element |
| TaCCHC38 | CAAT-box | 710 | 714 | conservative cis-element |
| TaCCHC38 | CAAT-box | 739 | 743 | conservative cis-element |
| TaCCHC38 | CAAT-box | 753 | 760 | conservative cis-element |
| TaCCHC38 | CAAT-box | 812 | 822 | conservative cis-element |
| TaCCHC38 | CAAT-box | 855 | 859 | conservative cis-element |
| TaCCHC38 | CAAT-box | 865 | 869 | conservative cis-element |
| TaCCHC38 | CAAT-box | 870 | 874 | conservative cis-element |
| TaCCHC38 | CAAT-box | 894 | 898 | conservative cis-element |
| TaCCHC38 | CAAT-box | 1066 | 1071 | conservative cis-element |
| TaCCHC38 | CAAT-box | 1067 | 1071 | conservative cis-element |
| TaCCHC38 | CAAT-box | 1087 | 1092 | conservative cis-element |
| TaCCHC38 | CAAT-box | 1088 | 1092 | conservative cis-element |
| TaCCHC38 | CAAT-box | 1264 | 1269 | conservative cis-element |
| TaCCHC38 | CAAT-box | 1265 | 1269 | conservative cis-element |
| TaCCHC38 | CAAT-box | 1401 | 1406 | conservative cis-element |
| TaCCHC38 | TATA-box | 18 | 22 | conservative cis-element |
| TaCCHC38 | TATA-box | 46 | 52 | conservative cis-element |
| TaCCHC38 | TATA-box | 47 | 52 | conservative cis-element |
| TaCCHC38 | TATA-box | 48 | 52 | conservative cis-element |
| TaCCHC38 | TATA-box | 373 | 377 | conservative cis-element |
| TaCCHC38 | TATA-box | 722 | 728 | conservative cis-element |
| TaCCHC38 | TATA-box | 723 | 727 | conservative cis-element |
| TaCCHC38 | TATA-box | 857 | 863 | conservative cis-element |
| TaCCHC38 | TATA-box | 858 | 862 | conservative cis-element |
| TaCCHC38 | TATA-box | 896 | 902 | conservative cis-element |
| TaCCHC38 | TATA-box | 897 | 901 | conservative cis-element |
| TaCCHC39 | TGACG-motif | 229 | 234 | MeJA responsive |
| TaCCHC39 | TGACG-motif | 311 | 316 | MeJA responsive |
| TaCCHC39 | TGACG-motif | 320 | 325 | MeJA responsive |
| TaCCHC39 | AuxRR-core | 548 | 555 | auxin responsive |
| TaCCHC39 | LTR | 526 | 532 | low-temperature responsive |
| TaCCHC39 | LTR | 942 | 948 | low-temperature responsive |
| TaCCHC39 | MBS | 968 | 974 | drought responsive |
| TaCCHC39 | MBS | 1374 | 1380 | drought responsive |
| TaCCHC39 | CGTCA-motif | 229 | 234 | MeJA responsive |
| TaCCHC39 | CGTCA-motif | 311 | 316 | MeJA responsive |
| TaCCHC39 | CGTCA-motif | 320 | 325 | MeJA responsive |
| TaCCHC39 | TC-rich repeats | 439 | 448 | defense and stress responsive |
| TaCCHC39 | TGA-element | 1311 | 1317 | auxin responsive |
| TaCCHC39 | WUN-motif | 368 | 377 | wound responsive |
| TaCCHC39 | ABRE | 776 | 783 | ABA responsive |
| TaCCHC39 | ARE | 445 | 451 | anaerobic induction |
| TaCCHC39 | ARE | 467 | 473 | anaerobic induction |
| TaCCHC39 | ARE | 689 | 695 | anaerobic induction |
| TaCCHC39 | TATA-box | 579 | 583 | conservative cis-element |
| TaCCHC39 | TATA-box | 710 | 719 | conservative cis-element |
| TaCCHC39 | TATA-box | 712 | 719 | conservative cis-element |
| TaCCHC39 | TATA-box | 1307 | 1311 | conservative cis-element |
| TaCCHC39 | TATA-box | 1392 | 1398 | conservative cis-element |
| TaCCHC39 | TATA-box | 1394 | 1398 | conservative cis-element |
| TaCCHC39 | TATA-box | 1431 | 1435 | conservative cis-element |
| TaCCHC39 | TATA-box | 1455 | 1461 | conservative cis-element |
| TaCCHC39 | TATA-box | 1456 | 1461 | conservative cis-element |
| TaCCHC39 | TATA-box | 1457 | 1461 | conservative cis-element |
| TaCCHC39 | CAAT-box | 317 | 322 | conservative cis-element |
| TaCCHC39 | CAAT-box | 455 | 459 | conservative cis-element |
| TaCCHC39 | CAAT-box | 544 | 549 | conservative cis-element |
| TaCCHC39 | CAAT-box | 631 | 635 | conservative cis-element |
| TaCCHC39 | CAAT-box | 646 | 651 | conservative cis-element |
| TaCCHC39 | CAAT-box | 647 | 651 | conservative cis-element |
| TaCCHC39 | CAAT-box | 1014 | 1019 | conservative cis-element |
| TaCCHC39 | CAAT-box | 1070 | 1075 | conservative cis-element |
| TaCCHC39 | CAAT-box | 1071 | 1075 | conservative cis-element |
| TaCCHC39 | CAAT-box | 1083 | 1088 | conservative cis-element |
| TaCCHC39 | CAAT-box | 1216 | 1221 | conservative cis-element |
| TaCCHC39 | CAAT-box | 1270 | 1274 | conservative cis-element |
| TaCCHC39 | CAAT-box | 1340 | 1345 | conservative cis-element |
| TaCCHC39 | CAAT-box | 1490 | 1495 | conservative cis-element |
| TaCCHC40 | TGACG-motif | 1186 | 1191 | MeJA responsive |
| TaCCHC40 | TGACG-motif | 1228 | 1233 | MeJA responsive |
| TaCCHC40 | AuxRR-core | 370 | 377 | auxin responsive |
| TaCCHC40 | LTR | 1469 | 1475 | low-temperature responsive |
| TaCCHC40 | MBS | 139 | 145 | drought responsive |
| TaCCHC40 | CGTCA-motif | 1186 | 1191 | MeJA responsive |
| TaCCHC40 | CGTCA-motif | 1228 | 1233 | MeJA responsive |
| TaCCHC40 | TGA-element | 144 | 150 | auxin responsive |
| TaCCHC40 | ERE | 235 | 243 | ethylene responsive |
| TaCCHC40 | GC-motif | 1220 | 1226 | anoxic specific inducibility |
| TaCCHC40 | GC-motif | 1472 | 1478 | anoxic specific inducibility |
| TaCCHC40 | ABRE | 721 | 726 | ABA responsive |
| TaCCHC40 | ABRE | 750 | 755 | ABA responsive |
| TaCCHC40 | ABRE | 818 | 824 | ABA responsive |
| TaCCHC40 | ABRE | 819 | 824 | ABA responsive |
| TaCCHC40 | ABRE | 1202 | 1211 | ABA responsive |
| TaCCHC40 | ABRE | 1204 | 1210 | ABA responsive |
| TaCCHC40 | ABRE | 1205 | 1210 | ABA responsive |
| TaCCHC40 | ABRE | 1226 | 1231 | ABA responsive |
| TaCCHC40 | ABRE | 1250 | 1255 | ABA responsive |
| TaCCHC40 | ARE | 444 | 450 | anaerobic induction |
| TaCCHC40 | ARE | 513 | 519 | anaerobic induction |
| TaCCHC40 | ARE | 912 | 918 | anaerobic induction |
| TaCCHC40 | ARE | 1161 | 1167 | anaerobic induction |
| TaCCHC40 | TATA-box | 334 | 338 | conservative cis-element |
| TaCCHC40 | TATA-box | 364 | 368 | conservative cis-element |
| TaCCHC40 | TATA-box | 879 | 885 | conservative cis-element |
| TaCCHC40 | TATA-box | 880 | 885 | conservative cis-element |
| TaCCHC40 | TATA-box | 881 | 885 | conservative cis-element |
| TaCCHC40 | TATA-box | 888 | 894 | conservative cis-element |
| TaCCHC40 | TATA-box | 889 | 893 | conservative cis-element |
| TaCCHC40 | TATA-box | 931 | 937 | conservative cis-element |
| TaCCHC40 | TATA-box | 932 | 937 | conservative cis-element |
| TaCCHC40 | TATA-box | 933 | 937 | conservative cis-element |
| TaCCHC40 | TATA-box | 935 | 942 | conservative cis-element |
| TaCCHC40 | TATA-box | 1353 | 1357 | conservative cis-element |
| TaCCHC40 | TATA-box | 1374 | 1380 | conservative cis-element |
| TaCCHC40 | TATA-box | 1375 | 1381 | conservative cis-element |
| TaCCHC40 | TATA-box | 1376 | 1380 | conservative cis-element |
| TaCCHC40 | CAAT-box | 9 | 13 | conservative cis-element |
| TaCCHC40 | CAAT-box | 35 | 39 | conservative cis-element |
| TaCCHC40 | CAAT-box | 50 | 55 | conservative cis-element |
| TaCCHC40 | CAAT-box | 116 | 120 | conservative cis-element |
| TaCCHC40 | CAAT-box | 118 | 122 | conservative cis-element |
| TaCCHC40 | CAAT-box | 324 | 328 | conservative cis-element |
| TaCCHC40 | CAAT-box | 326 | 330 | conservative cis-element |
| TaCCHC40 | CAAT-box | 475 | 480 | conservative cis-element |
| TaCCHC40 | CAAT-box | 706 | 710 | conservative cis-element |
| TaCCHC40 | CAAT-box | 863 | 868 | conservative cis-element |
| TaCCHC40 | CAAT-box | 872 | 876 | conservative cis-element |
| TaCCHC40 | CAAT-box | 892 | 897 | conservative cis-element |
| TaCCHC40 | CAAT-box | 915 | 920 | conservative cis-element |
| TaCCHC40 | CAAT-box | 916 | 920 | conservative cis-element |
| TaCCHC40 | CAAT-box | 950 | 955 | conservative cis-element |
| TaCCHC40 | CAAT-box | 953 | 957 | conservative cis-element |
| TaCCHC40 | CAAT-box | 1062 | 1067 | conservative cis-element |
| TaCCHC40 | CAAT-box | 1077 | 1081 | conservative cis-element |
| TaCCHC41 | ARE | 83 | 89 | anaerobic induction |
| TaCCHC41 | GC-motif | 1109 | 1115 | anoxic specific inducibility |
| TaCCHC41 | GC-motif | 1307 | 1313 | anoxic specific inducibility |
| TaCCHC41 | ABRE | 179 | 185 | ABA responsive |
| TaCCHC41 | ABRE | 180 | 185 | ABA responsive |
| TaCCHC41 | ABRE | 433 | 438 | ABA responsive |
| TaCCHC41 | TCA-element | 467 | 476 | salicylic acid responsive |
| TaCCHC41 | CGTCA-motif | 1118 | 1123 | MeJA responsive |
| TaCCHC41 | CGTCA-motif | 1442 | 1447 | MeJA responsive |
| TaCCHC41 | TGA-element | 216 | 222 | auxin responsive |
| TaCCHC41 | LTR | 1365 | 1371 | low-temperature responsive |
| TaCCHC41 | TGACG-motif | 1118 | 1123 | MeJA responsive |
| TaCCHC41 | TGACG-motif | 1442 | 1447 | MeJA responsive |
| TaCCHC41 | CAAT-box | 120 | 124 | conservative cis-element |
| TaCCHC41 | CAAT-box | 135 | 140 | conservative cis-element |
| TaCCHC41 | CAAT-box | 246 | 253 | conservative cis-element |
| TaCCHC41 | CAAT-box | 274 | 278 | conservative cis-element |
| TaCCHC41 | CAAT-box | 305 | 310 | conservative cis-element |
| TaCCHC41 | CAAT-box | 327 | 331 | conservative cis-element |
| TaCCHC41 | CAAT-box | 412 | 416 | conservative cis-element |
| TaCCHC41 | CAAT-box | 442 | 447 | conservative cis-element |
| TaCCHC41 | CAAT-box | 524 | 529 | conservative cis-element |
| TaCCHC41 | CAAT-box | 618 | 622 | conservative cis-element |
| TaCCHC41 | CAAT-box | 857 | 861 | conservative cis-element |
| TaCCHC41 | CAAT-box | 867 | 871 | conservative cis-element |
| TaCCHC41 | CAAT-box | 872 | 876 | conservative cis-element |
| TaCCHC41 | CAAT-box | 896 | 900 | conservative cis-element |
| TaCCHC41 | CAAT-box | 1060 | 1065 | conservative cis-element |
| TaCCHC41 | CAAT-box | 1061 | 1065 | conservative cis-element |
| TaCCHC41 | CAAT-box | 1254 | 1259 | conservative cis-element |
| TaCCHC41 | CAAT-box | 1255 | 1259 | conservative cis-element |
| TaCCHC41 | CAAT-box | 1403 | 1408 | conservative cis-element |
| TaCCHC41 | TATA-box | 38 | 42 | conservative cis-element |
| TaCCHC41 | TATA-box | 47 | 51 | conservative cis-element |
| TaCCHC41 | TATA-box | 105 | 111 | conservative cis-element |
| TaCCHC41 | TATA-box | 106 | 111 | conservative cis-element |
| TaCCHC41 | TATA-box | 107 | 111 | conservative cis-element |
| TaCCHC41 | TATA-box | 263 | 267 | conservative cis-element |
| TaCCHC41 | TATA-box | 574 | 580 | conservative cis-element |
| TaCCHC41 | TATA-box | 575 | 580 | conservative cis-element |
| TaCCHC41 | TATA-box | 576 | 580 | conservative cis-element |
| TaCCHC41 | TATA-box | 682 | 689 | conservative cis-element |
| TaCCHC41 | TATA-box | 786 | 790 | conservative cis-element |
| TaCCHC41 | TATA-box | 859 | 865 | conservative cis-element |
| TaCCHC41 | TATA-box | 860 | 864 | conservative cis-element |
| TaCCHC41 | TATA-box | 898 | 904 | conservative cis-element |
| TaCCHC41 | TATA-box | 899 | 903 | conservative cis-element |
| TaCCHC42 | LTR | 951 | 957 | low-temperature responsive |
| TaCCHC42 | MBS | 266 | 272 | drought responsive |
| TaCCHC42 | MBS | 977 | 983 | drought responsive |
| TaCCHC42 | MBS | 1374 | 1380 | drought responsive |
| TaCCHC42 | TCA-element | 557 | 566 | salicylic acid responsive |
| TaCCHC42 | ABRE | 338 | 347 | ABA responsive |
| TaCCHC42 | ABRE | 341 | 346 | ABA responsive |
| TaCCHC42 | ABRE | 369 | 374 | ABA responsive |
| TaCCHC42 | ARE | 115 | 121 | anaerobic induction |
| TaCCHC42 | ARE | 141 | 147 | anaerobic induction |
| TaCCHC42 | ARE | 372 | 378 | anaerobic induction |
| TaCCHC42 | ARE | 1463 | 1469 | anaerobic induction |
| TaCCHC42 | SARE | 615 | 626 | salicylic acid responsive |
| TaCCHC42 | TATA-box | 712 | 721 | conservative cis-element |
| TaCCHC42 | TATA-box | 714 | 721 | conservative cis-element |
| TaCCHC42 | TATA-box | 1352 | 1356 | conservative cis-element |
| TaCCHC42 | TATA-box | 1392 | 1398 | conservative cis-element |
| TaCCHC42 | TATA-box | 1394 | 1400 | conservative cis-element |
| TaCCHC42 | TATA-box | 1396 | 1400 | conservative cis-element |
| TaCCHC42 | TATA-box | 1431 | 1435 | conservative cis-element |
| TaCCHC42 | TATA-box | 1455 | 1461 | conservative cis-element |
| TaCCHC42 | TATA-box | 1456 | 1461 | conservative cis-element |
| TaCCHC42 | TATA-box | 1457 | 1461 | conservative cis-element |
| TaCCHC42 | CAAT-box | 4 | 9 | conservative cis-element |
| TaCCHC42 | CAAT-box | 10 | 14 | conservative cis-element |
| TaCCHC42 | CAAT-box | 40 | 44 | conservative cis-element |
| TaCCHC42 | CAAT-box | 74 | 79 | conservative cis-element |
| TaCCHC42 | CAAT-box | 130 | 134 | conservative cis-element |
| TaCCHC42 | CAAT-box | 455 | 459 | conservative cis-element |
| TaCCHC42 | CAAT-box | 470 | 475 | conservative cis-element |
| TaCCHC42 | CAAT-box | 497 | 502 | conservative cis-element |
| TaCCHC42 | CAAT-box | 509 | 514 | conservative cis-element |
| TaCCHC42 | CAAT-box | 633 | 637 | conservative cis-element |
| TaCCHC42 | CAAT-box | 648 | 653 | conservative cis-element |
| TaCCHC42 | CAAT-box | 649 | 653 | conservative cis-element |
| TaCCHC42 | CAAT-box | 1023 | 1028 | conservative cis-element |
| TaCCHC42 | CAAT-box | 1079 | 1084 | conservative cis-element |
| TaCCHC42 | CAAT-box | 1080 | 1084 | conservative cis-element |
| TaCCHC42 | CAAT-box | 1092 | 1097 | conservative cis-element |
| TaCCHC42 | CAAT-box | 1220 | 1224 | conservative cis-element |
| TaCCHC42 | CAAT-box | 1225 | 1230 | conservative cis-element |
| TaCCHC42 | CAAT-box | 1272 | 1276 | conservative cis-element |
| TaCCHC42 | CAAT-box | 1292 | 1297 | conservative cis-element |
| TaCCHC42 | CAAT-box | 1298 | 1303 | conservative cis-element |
| TaCCHC42 | CAAT-box | 1342 | 1347 | conservative cis-element |
| TaCCHC42 | CAAT-box | 1490 | 1495 | conservative cis-element |
| TaCCHC43 | CGTCA-motif | 1248 | 1253 | MeJA responsive |
| TaCCHC43 | LTR | 489 | 495 | low-temperature responsive |
| TaCCHC43 | LTR | 1487 | 1493 | low-temperature responsive |
| TaCCHC43 | TATC-box | 473 | 480 | gibberellin responsive |
| TaCCHC43 | TGACG-motif | 1248 | 1253 | MeJA responsive |
| TaCCHC43 | ARE | 1181 | 1187 | anaerobic induction |
| TaCCHC43 | ABRE | 1222 | 1231 | ABA responsive |
| TaCCHC43 | ABRE | 1224 | 1230 | ABA responsive |
| TaCCHC43 | ABRE | 1225 | 1230 | ABA responsive |
| TaCCHC43 | ABRE | 1246 | 1251 | ABA responsive |
| TaCCHC43 | ABRE | 1270 | 1275 | ABA responsive |
| TaCCHC43 | GC-motif | 552 | 558 | anoxic specific inducibility |
| TaCCHC43 | GC-motif | 1240 | 1246 | anoxic specific inducibility |
| TaCCHC43 | GC-motif | 1306 | 1312 | anoxic specific inducibility |
| TaCCHC43 | GC-motif | 1490 | 1496 | anoxic specific inducibility |
| TaCCHC43 | WUN-motif | 256 | 265 | wound responsive |
| TaCCHC43 | TATA-box | 463 | 472 | conservative cis-element |
| TaCCHC43 | TATA-box | 465 | 469 | conservative cis-element |
| TaCCHC43 | TATA-box | 645 | 654 | conservative cis-element |
| TaCCHC43 | TATA-box | 647 | 651 | conservative cis-element |
| TaCCHC43 | TATA-box | 750 | 754 | conservative cis-element |
| TaCCHC43 | TATA-box | 843 | 847 | conservative cis-element |
| TaCCHC43 | TATA-box | 958 | 967 | conservative cis-element |
| TaCCHC43 | TATA-box | 959 | 965 | conservative cis-element |
| TaCCHC43 | TATA-box | 960 | 967 | conservative cis-element |
| TaCCHC43 | TATA-box | 961 | 967 | conservative cis-element |
| TaCCHC43 | TATA-box | 963 | 967 | conservative cis-element |
| TaCCHC43 | TATA-box | 965 | 972 | conservative cis-element |
| TaCCHC43 | TATA-box | 1393 | 1399 | conservative cis-element |
| TaCCHC43 | TATA-box | 1394 | 1400 | conservative cis-element |
| TaCCHC43 | TATA-box | 1395 | 1399 | conservative cis-element |
| TaCCHC43 | CAAT-box | 68 | 73 | conservative cis-element |
| TaCCHC43 | CAAT-box | 341 | 345 | conservative cis-element |
| TaCCHC43 | CAAT-box | 354 | 358 | conservative cis-element |
| TaCCHC43 | CAAT-box | 362 | 367 | conservative cis-element |
| TaCCHC43 | CAAT-box | 363 | 367 | conservative cis-element |
| TaCCHC43 | CAAT-box | 420 | 425 | conservative cis-element |
| TaCCHC43 | CAAT-box | 421 | 425 | conservative cis-element |
| TaCCHC43 | CAAT-box | 432 | 437 | conservative cis-element |
| TaCCHC43 | CAAT-box | 454 | 459 | conservative cis-element |
| TaCCHC43 | CAAT-box | 495 | 500 | conservative cis-element |
| TaCCHC43 | CAAT-box | 508 | 513 | conservative cis-element |
| TaCCHC43 | CAAT-box | 535 | 539 | conservative cis-element |
| TaCCHC43 | CAAT-box | 803 | 807 | conservative cis-element |
| TaCCHC43 | CAAT-box | 943 | 948 | conservative cis-element |
| TaCCHC43 | CAAT-box | 946 | 950 | conservative cis-element |
| TaCCHC43 | CAAT-box | 981 | 986 | conservative cis-element |
| TaCCHC43 | CAAT-box | 984 | 988 | conservative cis-element |
| TaCCHC43 | CAAT-box | 1091 | 1096 | conservative cis-element |
| TaCCHC43 | CAAT-box | 1106 | 1110 | conservative cis-element |
| TaCCHC44 | TGACG-motif | 1018 | 1023 | MeJA responsive |
| TaCCHC44 | MBS | 538 | 544 | drought responsive |
| TaCCHC44 | GARE-motif | 370 | 377 | gibberellin responsive |
| TaCCHC44 | CGTCA-motif | 1018 | 1023 | MeJA responsive |
| TaCCHC44 | P-box | 820 | 827 | gibberellin responsive |
| TaCCHC44 | TGA-element | 175 | 181 | auxin responsive |
| TaCCHC44 | TGA-element | 245 | 251 | auxin responsive |
| TaCCHC44 | ABRE | 103 | 109 | ABA responsive |
| TaCCHC44 | ABRE | 104 | 109 | ABA responsive |
| TaCCHC44 | ABRE | 164 | 169 | ABA responsive |
| TaCCHC44 | TATA-box | 1 | 7 | conservative cis-element |
| TaCCHC44 | TATA-box | 2 | 7 | conservative cis-element |
| TaCCHC44 | TATA-box | 3 | 7 | conservative cis-element |
| TaCCHC44 | TATA-box | 508 | 512 | conservative cis-element |
| TaCCHC44 | TATA-box | 748 | 754 | conservative cis-element |
| TaCCHC44 | TATA-box | 749 | 753 | conservative cis-element |
| TaCCHC44 | TATA-box | 787 | 793 | conservative cis-element |
| TaCCHC44 | TATA-box | 788 | 792 | conservative cis-element |
| TaCCHC44 | CAAT-box | 79 | 83 | conservative cis-element |
| TaCCHC44 | CAAT-box | 155 | 160 | conservative cis-element |
| TaCCHC44 | CAAT-box | 156 | 160 | conservative cis-element |
| TaCCHC44 | CAAT-box | 169 | 173 | conservative cis-element |
| TaCCHC44 | CAAT-box | 217 | 221 | conservative cis-element |
| TaCCHC44 | CAAT-box | 459 | 464 | conservative cis-element |
| TaCCHC44 | CAAT-box | 568 | 573 | conservative cis-element |
| TaCCHC44 | CAAT-box | 647 | 651 | conservative cis-element |
| TaCCHC44 | CAAT-box | 657 | 661 | conservative cis-element |
| TaCCHC44 | CAAT-box | 663 | 667 | conservative cis-element |
| TaCCHC44 | CAAT-box | 716 | 720 | conservative cis-element |
| TaCCHC44 | CAAT-box | 746 | 750 | conservative cis-element |
| TaCCHC44 | CAAT-box | 756 | 760 | conservative cis-element |
| TaCCHC44 | CAAT-box | 761 | 765 | conservative cis-element |
| TaCCHC44 | CAAT-box | 785 | 789 | conservative cis-element |
| TaCCHC44 | CAAT-box | 962 | 967 | conservative cis-element |
| TaCCHC44 | CAAT-box | 963 | 967 | conservative cis-element |
| TaCCHC44 | CAAT-box | 979 | 984 | conservative cis-element |
| TaCCHC44 | CAAT-box | 980 | 984 | conservative cis-element |
| TaCCHC44 | CAAT-box | 1469 | 1474 | conservative cis-element |
| TaCCHC45 | LTR | 960 | 966 | low-temperature responsive |
| TaCCHC45 | GARE-motif | 231 | 238 | gibberellin responsive |
| TaCCHC45 | MBS | 986 | 992 | drought responsive |
| TaCCHC45 | MBS | 1374 | 1380 | drought responsive |
| TaCCHC45 | ERE | 352 | 360 | ethylene responsive |
| TaCCHC45 | ABRE | 787 | 794 | ABA responsive |
| TaCCHC45 | ARE | 1463 | 1469 | anaerobic induction |
| TaCCHC45 | TATA-box | 292 | 298 | conservative cis-element |
| TaCCHC45 | TATA-box | 293 | 297 | conservative cis-element |
| TaCCHC45 | TATA-box | 483 | 490 | conservative cis-element |
| TaCCHC45 | TATA-box | 484 | 490 | conservative cis-element |
| TaCCHC45 | TATA-box | 485 | 490 | conservative cis-element |
| TaCCHC45 | TATA-box | 486 | 490 | conservative cis-element |
| TaCCHC45 | TATA-box | 717 | 726 | conservative cis-element |
| TaCCHC45 | TATA-box | 719 | 726 | conservative cis-element |
| TaCCHC45 | TATA-box | 1392 | 1398 | conservative cis-element |
| TaCCHC45 | TATA-box | 1394 | 1400 | conservative cis-element |
| TaCCHC45 | TATA-box | 1395 | 1401 | conservative cis-element |
| TaCCHC45 | TATA-box | 1396 | 1400 | conservative cis-element |
| TaCCHC45 | TATA-box | 1431 | 1435 | conservative cis-element |
| TaCCHC45 | TATA-box | 1455 | 1461 | conservative cis-element |
| TaCCHC45 | TATA-box | 1456 | 1461 | conservative cis-element |
| TaCCHC45 | TATA-box | 1457 | 1461 | conservative cis-element |
| TaCCHC45 | CAAT-box | 255 | 259 | conservative cis-element |
| TaCCHC45 | CAAT-box | 270 | 275 | conservative cis-element |
| TaCCHC45 | CAAT-box | 300 | 304 | conservative cis-element |
| TaCCHC45 | CAAT-box | 330 | 335 | conservative cis-element |
| TaCCHC45 | CAAT-box | 331 | 335 | conservative cis-element |
| TaCCHC45 | CAAT-box | 344 | 348 | conservative cis-element |
| TaCCHC45 | CAAT-box | 654 | 658 | conservative cis-element |
| TaCCHC45 | CAAT-box | 1032 | 1037 | conservative cis-element |
| TaCCHC45 | CAAT-box | 1088 | 1093 | conservative cis-element |
| TaCCHC45 | CAAT-box | 1089 | 1093 | conservative cis-element |
| TaCCHC45 | CAAT-box | 1101 | 1106 | conservative cis-element |
| TaCCHC45 | CAAT-box | 1234 | 1239 | conservative cis-element |
| TaCCHC45 | CAAT-box | 1288 | 1292 | conservative cis-element |
| TaCCHC45 | CAAT-box | 1307 | 1312 | conservative cis-element |
| TaCCHC45 | CAAT-box | 1313 | 1318 | conservative cis-element |
| TaCCHC45 | CAAT-box | 1342 | 1347 | conservative cis-element |
| TaCCHC45 | CAAT-box | 1490 | 1495 | conservative cis-element |
| TaCCHC46 | TGACG-motif | 400 | 405 | MeJA responsive |
| TaCCHC46 | TGACG-motif | 618 | 623 | MeJA responsive |
| TaCCHC46 | AuxRR-core | 587 | 594 | auxin responsive |
| TaCCHC46 | LTR | 1313 | 1319 | low-temperature responsive |
| TaCCHC46 | LTR | 1403 | 1409 | low-temperature responsive |
| TaCCHC46 | MBS | 10 | 16 | drought responsive |
| TaCCHC46 | MBS | 875 | 881 | drought responsive |
| TaCCHC46 | CGTCA-motif | 400 | 405 | MeJA responsive |
| TaCCHC46 | CGTCA-motif | 618 | 623 | MeJA responsive |
| TaCCHC46 | ABRE | 15 | 24 | ABA responsive |
| TaCCHC46 | ABRE | 144 | 150 | ABA responsive |
| TaCCHC46 | ABRE | 145 | 150 | ABA responsive |
| TaCCHC46 | ABRE | 493 | 498 | ABA responsive |
| TaCCHC46 | ABRE | 1187 | 1196 | ABA responsive |
| TaCCHC46 | ARE | 1052 | 1058 | anaerobic induction |
| TaCCHC46 | TATA-box | 352 | 356 | conservative cis-element |
| TaCCHC46 | TATA-box | 515 | 521 | conservative cis-element |
| TaCCHC46 | TATA-box | 516 | 521 | conservative cis-element |
| TaCCHC46 | TATA-box | 517 | 521 | conservative cis-element |
| TaCCHC46 | TATA-box | 690 | 696 | conservative cis-element |
| TaCCHC46 | TATA-box | 691 | 695 | conservative cis-element |
| TaCCHC46 | TATA-box | 839 | 843 | conservative cis-element |
| TaCCHC46 | TATA-box | 923 | 932 | conservative cis-element |
| TaCCHC46 | TATA-box | 1107 | 1113 | conservative cis-element |
| TaCCHC46 | TATA-box | 1108 | 1112 | conservative cis-element |
| TaCCHC46 | CAAT-box | 75 | 80 | conservative cis-element |
| TaCCHC46 | CAAT-box | 159 | 163 | conservative cis-element |
| TaCCHC46 | CAAT-box | 170 | 175 | conservative cis-element |
| TaCCHC46 | CAAT-box | 244 | 249 | conservative cis-element |
| TaCCHC46 | CAAT-box | 306 | 311 | conservative cis-element |
| TaCCHC46 | CAAT-box | 363 | 368 | conservative cis-element |
| TaCCHC46 | CAAT-box | 602 | 606 | conservative cis-element |
| TaCCHC46 | CAAT-box | 608 | 613 | conservative cis-element |
| TaCCHC46 | CAAT-box | 909 | 913 | conservative cis-element |
| TaCCHC46 | CAAT-box | 949 | 954 | conservative cis-element |
| TaCCHC46 | CAAT-box | 1015 | 1020 | conservative cis-element |
| TaCCHC46 | CAAT-box | 1016 | 1020 | conservative cis-element |
| TaCCHC46 | CAAT-box | 1105 | 1109 | conservative cis-element |
| TaCCHC46 | CAAT-box | 1413 | 1417 | conservative cis-element |
| TaCCHC47 | TGACG-motif | 766 | 771 | MeJA responsive |
| TaCCHC47 | GARE-motif | 398 | 405 | gibberellin responsive |
| TaCCHC47 | MBS | 922 | 928 | drought responsive |
| TaCCHC47 | MBS | 1213 | 1219 | drought responsive |
| TaCCHC47 | MBS | 1261 | 1267 | drought responsive |
| TaCCHC47 | MBS | 1479 | 1485 | drought responsive |
| TaCCHC47 | TCA-element | 408 | 417 | salicylic acid responsive |
| TaCCHC47 | CGTCA-motif | 766 | 771 | MeJA responsive |
| TaCCHC47 | P-box | 657 | 664 | gibberellin responsive |
| TaCCHC47 | P-box | 946 | 953 | gibberellin responsive |
| TaCCHC47 | TC-rich repeats | 403 | 412 | defense and stress responsive |
| TaCCHC47 | ERE | 874 | 882 | ethylene responsive |
| TaCCHC47 | TGA-element | 1132 | 1138 | auxin responsive |
| TaCCHC47 | ARE | 951 | 957 | anaerobic induction |
| TaCCHC47 | TATA-box | 3 | 11 | conservative cis-element |
| TaCCHC47 | TATA-box | 606 | 612 | conservative cis-element |
| TaCCHC47 | TATA-box | 607 | 613 | conservative cis-element |
| TaCCHC47 | TATA-box | 608 | 612 | conservative cis-element |
| TaCCHC47 | TATA-box | 741 | 745 | conservative cis-element |
| TaCCHC47 | TATA-box | 929 | 933 | conservative cis-element |
| TaCCHC47 | TATA-box | 1319 | 1325 | conservative cis-element |
| TaCCHC47 | TATA-box | 1320 | 1326 | conservative cis-element |
| TaCCHC47 | TATA-box | 1321 | 1327 | conservative cis-element |
| TaCCHC47 | TATA-box | 1322 | 1326 | conservative cis-element |
| TaCCHC47 | CAAT-box | 82 | 86 | conservative cis-element |
| TaCCHC47 | CAAT-box | 185 | 189 | conservative cis-element |
| TaCCHC47 | CAAT-box | 282 | 287 | conservative cis-element |
| TaCCHC47 | CAAT-box | 320 | 324 | conservative cis-element |
| TaCCHC47 | CAAT-box | 457 | 462 | conservative cis-element |
| TaCCHC47 | CAAT-box | 611 | 615 | conservative cis-element |
| TaCCHC47 | CAAT-box | 730 | 734 | conservative cis-element |
| TaCCHC47 | CAAT-box | 769 | 773 | conservative cis-element |
| TaCCHC47 | CAAT-box | 957 | 961 | conservative cis-element |
| TaCCHC47 | CAAT-box | 1077 | 1081 | conservative cis-element |
| TaCCHC47 | CAAT-box | 1086 | 1090 | conservative cis-element |
| TaCCHC47 | CAAT-box | 1088 | 1093 | conservative cis-element |
| TaCCHC47 | CAAT-box | 1284 | 1288 | conservative cis-element |
| TaCCHC47 | CAAT-box | 1314 | 1318 | conservative cis-element |
| TaCCHC47 | CAAT-box | 1325 | 1329 | conservative cis-element |
| TaCCHC47 | CAAT-box | 1354 | 1358 | conservative cis-element |
| TaCCHC48 | GARE-motif | 1425 | 1432 | gibberellin responsive |
| TaCCHC48 | AuxRR-core | 955 | 962 | auxin responsive |
| TaCCHC48 | ABRE | 159 | 164 | ABA responsive |
| TaCCHC48 | ABRE | 231 | 236 | ABA responsive |
| TaCCHC48 | ABRE | 1367 | 1374 | ABA responsive |
| TaCCHC48 | ARE | 1052 | 1058 | anaerobic induction |
| TaCCHC48 | TGA-element | 1356 | 1362 | auxin responsive |
| TaCCHC48 | WUN-motif | 485 | 494 | wound responsive |
| TaCCHC48 | WUN-motif | 486 | 494 | wound responsive |
| TaCCHC48 | TATA-box | 40 | 44 | conservative cis-element |
| TaCCHC48 | TATA-box | 85 | 91 | conservative cis-element |
| TaCCHC48 | TATA-box | 86 | 90 | conservative cis-element |
| TaCCHC48 | TATA-box | 281 | 287 | conservative cis-element |
| TaCCHC48 | TATA-box | 282 | 286 | conservative cis-element |
| TaCCHC48 | TATA-box | 291 | 295 | conservative cis-element |
| TaCCHC48 | TATA-box | 381 | 387 | conservative cis-element |
| TaCCHC48 | TATA-box | 382 | 386 | conservative cis-element |
| TaCCHC48 | TATA-box | 393 | 400 | conservative cis-element |
| TaCCHC48 | TATA-box | 675 | 682 | conservative cis-element |
| TaCCHC48 | TATA-box | 676 | 682 | conservative cis-element |
| TaCCHC48 | TATA-box | 677 | 682 | conservative cis-element |
| TaCCHC48 | TATA-box | 678 | 682 | conservative cis-element |
| TaCCHC48 | TATA-box | 776 | 780 | conservative cis-element |
| TaCCHC48 | TATA-box | 849 | 856 | conservative cis-element |
| TaCCHC48 | TATA-box | 850 | 856 | conservative cis-element |
| TaCCHC48 | TATA-box | 851 | 856 | conservative cis-element |
| TaCCHC48 | TATA-box | 852 | 856 | conservative cis-element |
| TaCCHC48 | TATA-box | 877 | 884 | conservative cis-element |
| TaCCHC48 | TATA-box | 878 | 884 | conservative cis-element |
| TaCCHC48 | TATA-box | 879 | 884 | conservative cis-element |
| TaCCHC48 | TATA-box | 880 | 884 | conservative cis-element |
| TaCCHC48 | TATA-box | 1108 | 1114 | conservative cis-element |
| TaCCHC48 | TATA-box | 1109 | 1113 | conservative cis-element |
| TaCCHC48 | CAAT-box | 257 | 261 | conservative cis-element |
| TaCCHC48 | CAAT-box | 300 | 304 | conservative cis-element |
| TaCCHC48 | CAAT-box | 305 | 309 | conservative cis-element |
| TaCCHC48 | CAAT-box | 338 | 342 | conservative cis-element |
| TaCCHC48 | CAAT-box | 385 | 389 | conservative cis-element |
| TaCCHC48 | CAAT-box | 390 | 394 | conservative cis-element |
| TaCCHC48 | CAAT-box | 458 | 462 | conservative cis-element |
| TaCCHC48 | CAAT-box | 478 | 482 | conservative cis-element |
| TaCCHC48 | CAAT-box | 497 | 502 | conservative cis-element |
| TaCCHC48 | CAAT-box | 572 | 576 | conservative cis-element |
| TaCCHC48 | CAAT-box | 697 | 701 | conservative cis-element |
| TaCCHC48 | CAAT-box | 699 | 703 | conservative cis-element |
| TaCCHC48 | CAAT-box | 791 | 796 | conservative cis-element |
| TaCCHC48 | CAAT-box | 870 | 874 | conservative cis-element |
| TaCCHC48 | CAAT-box | 948 | 953 | conservative cis-element |
| TaCCHC48 | CAAT-box | 1106 | 1110 | conservative cis-element |
| TaCCHC48 | CAAT-box | 1412 | 1416 | conservative cis-element |
| TaCCHC49 | P-box | 658 | 665 | gibberellin responsive |
| TaCCHC49 | ERE | 882 | 890 | ethylene responsive |
| TaCCHC49 | TGA-element | 1147 | 1153 | auxin responsive |
| TaCCHC49 | TGACG-motif | 1341 | 1346 | MeJA responsive |
| TaCCHC49 | CGTCA-motif | 1341 | 1346 | MeJA responsive |
| TaCCHC49 | MBS | 1259 | 1265 | drought responsive |
| TaCCHC49 | LTR | 981 | 987 | low-temperature responsive |
| TaCCHC49 | CAAT-box | 113 | 118 | conservative cis-element |
| TaCCHC49 | CAAT-box | 187 | 191 | conservative cis-element |
| TaCCHC49 | CAAT-box | 216 | 220 | conservative cis-element |
| TaCCHC49 | CAAT-box | 266 | 270 | conservative cis-element |
| TaCCHC49 | CAAT-box | 288 | 292 | conservative cis-element |
| TaCCHC49 | CAAT-box | 385 | 390 | conservative cis-element |
| TaCCHC49 | CAAT-box | 500 | 505 | conservative cis-element |
| TaCCHC49 | CAAT-box | 592 | 597 | conservative cis-element |
| TaCCHC49 | CAAT-box | 615 | 620 | conservative cis-element |
| TaCCHC49 | CAAT-box | 730 | 734 | conservative cis-element |
| TaCCHC49 | CAAT-box | 769 | 773 | conservative cis-element |
| TaCCHC49 | CAAT-box | 965 | 969 | conservative cis-element |
| TaCCHC49 | CAAT-box | 1090 | 1094 | conservative cis-element |
| TaCCHC49 | CAAT-box | 1099 | 1103 | conservative cis-element |
| TaCCHC49 | CAAT-box | 1101 | 1106 | conservative cis-element |
| TaCCHC49 | CAAT-box | 1153 | 1158 | conservative cis-element |
| TaCCHC49 | CAAT-box | 1154 | 1158 | conservative cis-element |
| TaCCHC49 | CAAT-box | 1240 | 1244 | conservative cis-element |
| TaCCHC49 | CAAT-box | 1256 | 1263 | conservative cis-element |
| TaCCHC49 | CAAT-box | 1282 | 1286 | conservative cis-element |
| TaCCHC49 | CAAT-box | 1312 | 1316 | conservative cis-element |
| TaCCHC49 | CAAT-box | 1323 | 1327 | conservative cis-element |
| TaCCHC49 | CAAT-box | 1352 | 1356 | conservative cis-element |
| TaCCHC49 | CAAT-box | 1386 | 1391 | conservative cis-element |
| TaCCHC49 | TATA-box | 67 | 71 | conservative cis-element |
| TaCCHC49 | TATA-box | 84 | 90 | conservative cis-element |
| TaCCHC49 | TATA-box | 86 | 90 | conservative cis-element |
| TaCCHC49 | TATA-box | 116 | 122 | conservative cis-element |
| TaCCHC49 | TATA-box | 117 | 121 | conservative cis-element |
| TaCCHC49 | TATA-box | 179 | 186 | conservative cis-element |
| TaCCHC49 | TATA-box | 201 | 210 | conservative cis-element |
| TaCCHC49 | TATA-box | 248 | 252 | conservative cis-element |
| TaCCHC49 | TATA-box | 610 | 616 | conservative cis-element |
| TaCCHC49 | TATA-box | 611 | 617 | conservative cis-element |
| TaCCHC49 | TATA-box | 612 | 616 | conservative cis-element |
| TaCCHC49 | TATA-box | 741 | 745 | conservative cis-element |
| TaCCHC49 | TATA-box | 937 | 941 | conservative cis-element |
| TaCCHC49 | TATA-box | 1317 | 1323 | conservative cis-element |
| TaCCHC49 | TATA-box | 1318 | 1324 | conservative cis-element |
| TaCCHC49 | TATA-box | 1319 | 1325 | conservative cis-element |
| TaCCHC49 | TATA-box | 1320 | 1324 | conservative cis-element |
| TaCCHC49 | TATA-box | 1466 | 1471 | conservative cis-element |
| TaCCHC49 | TATA-box | 1467 | 1471 | conservative cis-element |
| TaCCHC50 | MBS | 955 | 961 | drought responsive |
| TaCCHC50 | MBS | 1251 | 1257 | drought responsive |
| TaCCHC50 | MBS | 1299 | 1305 | drought responsive |
| TaCCHC50 | ERE | 907 | 915 | ethylene responsive |
| TaCCHC50 | ABRE | 30 | 35 | ABA responsive |
| TaCCHC50 | TATA-box | 322 | 329 | conservative cis-element |
| TaCCHC50 | TATA-box | 433 | 442 | conservative cis-element |
| TaCCHC50 | TATA-box | 435 | 439 | conservative cis-element |
| TaCCHC50 | TATA-box | 490 | 497 | conservative cis-element |
| TaCCHC50 | TATA-box | 640 | 646 | conservative cis-element |
| TaCCHC50 | TATA-box | 641 | 647 | conservative cis-element |
| TaCCHC50 | TATA-box | 642 | 646 | conservative cis-element |
| TaCCHC50 | TATA-box | 773 | 777 | conservative cis-element |
| TaCCHC50 | TATA-box | 962 | 966 | conservative cis-element |
| TaCCHC50 | TATA-box | 1319 | 1325 | conservative cis-element |
| TaCCHC50 | TATA-box | 1320 | 1326 | conservative cis-element |
| TaCCHC50 | TATA-box | 1321 | 1327 | conservative cis-element |
| TaCCHC50 | TATA-box | 1322 | 1326 | conservative cis-element |
| TaCCHC50 | TATA-box | 1466 | 1471 | conservative cis-element |
| TaCCHC50 | TATA-box | 1467 | 1471 | conservative cis-element |
| TaCCHC50 | CAAT-box | 8 | 13 | conservative cis-element |
| TaCCHC50 | CAAT-box | 70 | 75 | conservative cis-element |
| TaCCHC50 | CAAT-box | 154 | 158 | conservative cis-element |
| TaCCHC50 | CAAT-box | 163 | 168 | conservative cis-element |
| TaCCHC50 | CAAT-box | 219 | 223 | conservative cis-element |
| TaCCHC50 | CAAT-box | 254 | 258 | conservative cis-element |
| TaCCHC50 | CAAT-box | 531 | 536 | conservative cis-element |
| TaCCHC50 | CAAT-box | 579 | 583 | conservative cis-element |
| TaCCHC50 | CAAT-box | 645 | 649 | conservative cis-element |
| TaCCHC50 | CAAT-box | 762 | 766 | conservative cis-element |
| TaCCHC50 | CAAT-box | 800 | 805 | conservative cis-element |
| TaCCHC50 | CAAT-box | 801 | 805 | conservative cis-element |
| TaCCHC50 | CAAT-box | 932 | 936 | conservative cis-element |
| TaCCHC50 | CAAT-box | 990 | 994 | conservative cis-element |
| TaCCHC50 | CAAT-box | 1115 | 1119 | conservative cis-element |
| TaCCHC50 | CAAT-box | 1124 | 1128 | conservative cis-element |
| TaCCHC50 | CAAT-box | 1126 | 1131 | conservative cis-element |
| TaCCHC50 | CAAT-box | 1178 | 1183 | conservative cis-element |
| TaCCHC50 | CAAT-box | 1179 | 1183 | conservative cis-element |
| TaCCHC50 | CAAT-box | 1325 | 1329 | conservative cis-element |
| TaCCHC50 | CAAT-box | 1354 | 1358 | conservative cis-element |
| TaCCHC50 | CAAT-box | 1388 | 1393 | conservative cis-element |

Note: GA: gibberellins; ABA: abscisic acid; MeJA: methyl jasmonate; SA: salicylic acid

**Supplementary Table 8.** Potential interaction of miRNA and *TaCCHC-ZF* genes.

| **Target Gene** | **miRNA ID** | **Expectation** | **miRNA sequence** | **Inhibition** |
| --- | --- | --- | --- | --- |
| TaCCHC21 | tae-miR1121 | 1 | AGUAGUGAUCUAAACGCUCUUA | Cleavage |
| TaCCHC21 | tae-miR6197-5p | 1.5 | UCUGUAAACAAAUGUAGGACG | Cleavage |
| TaCCHC36 | tae-miR399 | 2.5 | UGCCAAAGGAGAAUUGCCC | Cleavage |
| TaCCHC28 | tae-miR399 | 2.5 | UGCCAAAGGAGAAUUGCCC | Cleavage |
| TaCCHC47 | tae-miR9672b | 3 | UACCACGACUGUCAUUAAGCA | Cleavage |
| TaCCHC49 | tae-miR9672b | 3 | UACCACGACUGUCAUUAAGCA | Cleavage |
| TaCCHC50 | tae-miR9672b | 3 | UACCACGACUGUCAUUAAGCA | Cleavage |
| TaCCHC42 | tae-miR9652-5p | 3.5 | CCUGUUUGUCAUUAAGUUUCUU | Cleavage |
| TaCCHC45 | tae-miR9652-5p | 3.5 | CCUGUUUGUCAUUAAGUUUCUU | Cleavage |
| TaCCHC28 | tae-miR9655-3p | 3.5 | CAAGGGAAGGAAGUAGCCAAC | Cleavage |
| TaCCHC30 | tae-miR9655-3p | 4 | CAAGGGAAGGAAGUAGCCAAC | Translation |
| TaCCHC47 | tae-miR9674a-5p | 4 | GCAUCAUCCAUCCUACCAUUC | Translation |
| TaCCHC13 | tae-miR9776 | 4 | UUGGACGAGGAUGUGCAACUG | Cleavage |
| TaCCHC6 | tae-miR9776 | 4 | UUGGACGAGGAUGUGCAACUG | Cleavage |
| TaCCHC22 | tae-miR9783 | 4 | AUAAGCACCGGUGCUUAAGAA | Cleavage |
| TaCCHC13 | tae-miR1121 | 4.5 | AGUAGUGAUCUAAACGCUCUUA | Translation |
| TaCCHC2 | tae-miR1137b-5p | 4.5 | UCCGUUCCAGAAUAGAUGACC | Cleavage |
| TaCCHC9 | tae-miR395b | 4.5 | UGAAGUGUUUGGGGGAACUC | Cleavage |
| TaCCHC45 | tae-miR5048-5p | 4.5 | UUUGCAGGUUUUAGGUCUAAGU | Cleavage |
| TaCCHC42 | tae-miR5048-5p | 4.5 | UUUGCAGGUUUUAGGUCUAAGU | Cleavage |
| TaCCHC26 | tae-miR9657b-5p | 4.5 | UUCGUCGGAGAAGCAUGUUGC | Cleavage |
| TaCCHC43 | tae-miR9664-3p | 4.5 | UUGCAGUCCUCGAUGUCGUAG | Cleavage |
| TaCCHC21 | tae-miR9667-5p | 4.5 | AAAUAUGGCAAACAAUGAAUG | Cleavage |
| TaCCHC50 | tae-miR9672a-3p | 4.5 | CCACGACUGUCAUUAAGCAUC | Cleavage |
| TaCCHC47 | tae-miR9672a-3p | 4.5 | CCACGACUGUCAUUAAGCAUC | Cleavage |
| TaCCHC49 | tae-miR9672a-3p | 4.5 | CCACGACUGUCAUUAAGCAUC | Cleavage |
| TaCCHC36 | tae-miR9675-3p | 4.5 | UUUAUGAUCACUCUCGUUUUG | Cleavage |
| TaCCHC17 | tae-miR9677b | 4.5 | CAGGGCGGGGAACAGGUGGCC | Cleavage |
| TaCCHC12 | tae-miR9677b | 4.5 | CAGGGCGGGGAACAGGUGGCC | Cleavage |
| TaCCHC1 | tae-miR9782 | 4.5 | GUAUUAGGUUGGUCAAAUUGACGA | Cleavage |
| TaCCHC9 | tae-miR9782 | 4.5 | GUAUUAGGUUGGUCAAAUUGACGA | Cleavage |
| TaCCHC36 | tae-miR1120a | 5 | ACAUUCUUAUAUUAUGAGACGGAG | Cleavage |
| TaCCHC21 | tae-miR1120c-5p | 5 | UAAUAUAAGAACGUUUUUGAC | Translation |
| TaCCHC1 | tae-miR1121 | 5 | AGUAGUGAUCUAAACGCUCUUA | Cleavage |
| TaCCHC9 | tae-miR1121 | 5 | AGUAGUGAUCUAAACGCUCUUA | Cleavage |
| TaCCHC26 | tae-miR1124 | 5 | GCAGGACGUGAAGAGCGAGUCC | Cleavage |
| TaCCHC35 | tae-miR1127a | 5 | UCCUUCCGUUCGGAAUUAC | Cleavage |
| TaCCHC21 | tae-miR1134 | 5 | CAACAACAACAAGAAGAAGAAGAU | Cleavage |
| TaCCHC9 | tae-miR1135 | 5 | CUGCGACAAGUAAUUCCGAACGGA | Cleavage |
| TaCCHC50 | tae-miR1136 | 5 | UUGUCGCAGGUAUGGAUGUAUCUA | Translation |
| TaCCHC15 | tae-miR1136 | 5 | UUGUCGCAGGUAUGGAUGUAUCUA | Cleavage |
| TaCCHC2 | tae-miR1136 | 5 | UUGUCGCAGGUAUGGAUGUAUCUA | Cleavage |
| TaCCHC10 | tae-miR1137b-5p | 5 | UCCGUUCCAGAAUAGAUGACC | Cleavage |
| TaCCHC15 | tae-miR1137b-5p | 5 | UCCGUUCCAGAAUAGAUGACC | Cleavage |
| TaCCHC1 | tae-miR156 | 5 | UGACAGAAGAGAGUGAGCACA | Cleavage |
| TaCCHC31 | tae-miR159a | 5 | UUUGGAUUGAAGGGAGCUCUG | Cleavage |
| TaCCHC35 | tae-miR159a | 5 | UUUGGAUUGAAGGGAGCUCUG | Cleavage |
| TaCCHC31 | tae-miR159b | 5 | UUUGGAUUGAAGGGAGCUCUG | Cleavage |
| TaCCHC35 | tae-miR159b | 5 | UUUGGAUUGAAGGGAGCUCUG | Cleavage |
| TaCCHC36 | tae-miR164 | 5 | UGGAGAAGCAGGGCACGUGCA | Cleavage |
| TaCCHC36 | tae-miR167a | 5 | UGAAGCUGCCAGCAUGAUCUA | Cleavage |
| TaCCHC36 | tae-miR167c-5p | 5 | UGAAGCUGCCAGCAUGAUCUGC | Cleavage |
| TaCCHC5 | tae-miR169 | 5 | GGGCAAGUCACCCUGGGCUACC | Translation |
| TaCCHC24 | tae-miR171b | 5 | UUGAGCCGUGCCAAUAUCACG | Cleavage |
| TaCCHC26 | tae-miR1847-5p | 5 | ACCUGCAGUUGGGCCAAUGAC | Cleavage |
| TaCCHC25 | tae-miR1847-5p | 5 | ACCUGCAGUUGGGCCAAUGAC | Cleavage |
| TaCCHC27 | tae-miR1847-5p | 5 | ACCUGCAGUUGGGCCAAUGAC | Cleavage |
| TaCCHC2 | tae-miR2275-3p | 5 | UUUGGUUUCCUCCAAUAUCUCG | Translation |
| TaCCHC44 | tae-miR395a | 5 | GUGAAGUGUUUGGGGGAACUC | Cleavage |
| TaCCHC44 | tae-miR395b | 5 | UGAAGUGUUUGGGGGAACUC | Cleavage |
| TaCCHC4 | tae-miR5048-5p | 5 | UUUGCAGGUUUUAGGUCUAAGU | Cleavage |
| TaCCHC12 | tae-miR5048-5p | 5 | UUUGCAGGUUUUAGGUCUAAGU | Cleavage |
| TaCCHC31 | tae-miR5085 | 5 | AAGGACAUUUUUUGUGGCCUG | Cleavage |
| TaCCHC35 | tae-miR5085 | 5 | AAGGACAUUUUUUGUGGCCUG | Cleavage |
| TaCCHC26 | tae-miR5384-3p | 5 | UGAGCGCGCCGCCGUCGAAUG | Cleavage |
| TaCCHC30 | tae-miR9652-3p | 5 | AAGCUUAAUGAGAACAUGUG | Cleavage |
| TaCCHC31 | tae-miR9652-5p | 5 | CCUGUUUGUCAUUAAGUUUCUU | Cleavage |
| TaCCHC35 | tae-miR9652-5p | 5 | CCUGUUUGUCAUUAAGUUUCUU | Cleavage |
| TaCCHC13 | tae-miR9652-5p | 5 | CCUGUUUGUCAUUAAGUUUCUU | Cleavage |
| TaCCHC50 | tae-miR9652-5p | 5 | CCUGUUUGUCAUUAAGUUUCUU | Cleavage |
| TaCCHC47 | tae-miR9652-5p | 5 | CCUGUUUGUCAUUAAGUUUCUU | Cleavage |
| TaCCHC49 | tae-miR9652-5p | 5 | CCUGUUUGUCAUUAAGUUUCUU | Cleavage |
| TaCCHC39 | tae-miR9652-5p | 5 | CCUGUUUGUCAUUAAGUUUCUU | Translation |
| TaCCHC26 | tae-miR9657b-3p | 5 | CGUGCUUCCUCGUCGAACGGU | Cleavage |
| TaCCHC10 | tae-miR9657b-5p | 5 | UUCGUCGGAGAAGCAUGUUGC | Cleavage |
| TaCCHC26 | tae-miR9657c-3p | 5 | CGUGCUUCCUCGUCGAACGGU | Cleavage |
| TaCCHC50 | tae-miR9670-3p | 5 | AGGUGGAAUACUUGAAGAAGA | Translation |
| TaCCHC50 | tae-miR9674a-5p | 5 | GCAUCAUCCAUCCUACCAUUC | Translation |
| TaCCHC8 | tae-miR9676-5p | 5 | UGGAUGUCAUCGUGGCCGUACA | Cleavage |
| TaCCHC3 | tae-miR9676-5p | 5 | UGGAUGUCAUCGUGGCCGUACA | Cleavage |
| TaCCHC11 | tae-miR9676-5p | 5 | UGGAUGUCAUCGUGGCCGUACA | Cleavage |
| TaCCHC43 | tae-miR9677a | 5 | UGGCCGUUGGUAGAGUAGGAGA | Cleavage |
| TaCCHC37 | tae-miR9677a | 5 | UGGCCGUUGGUAGAGUAGGAGA | Cleavage |
| TaCCHC21 | tae-miR9677b | 5 | CAGGGCGGGGAACAGGUGGCC | Cleavage |
| TaCCHC28 | tae-miR9679-5p | 5 | CAGAACCAGAAUGAGUAGCUC | Cleavage |
| TaCCHC36 | tae-miR9679-5p | 5 | CAGAACCAGAAUGAGUAGCUC | Cleavage |
| TaCCHC15 | tae-miR9780 | 5 | CGGGUCGGCGCUGCACGCGGC | Cleavage |
| TaCCHC12 | tae-miR9782 | 5 | GUAUUAGGUUGGUCAAAUUGACGA | Cleavage |
| TaCCHC17 | tae-miR9782 | 5 | GUAUUAGGUUGGUCAAAUUGACGA | Cleavage |
| TaCCHC4 | tae-miR9782 | 5 | GUAUUAGGUUGGUCAAAUUGACGA | Cleavage |
| TaCCHC50 | tae-miR9782 | 5 | GUAUUAGGUUGGUCAAAUUGACGA | Cleavage |

**Supplementary Table 9.** DEGs under different treatments.

| **DH-6h** | **HS-1h** | **HS-6h** | **DHS-1h** | **DHS-6h** | **Cold** |
| --- | --- | --- | --- | --- | --- |
| TaCCHC18 | TaCCHC2 | TaCCHC3 | TaCCHC1 | TaCCHC4 | TaCCHC2 |
| TaCCHC40 | TaCCHC3 | TaCCHC4 | TaCCHC3 | TaCCHC7 | TaCCHC10 |
|  | TaCCHC7 | TaCCHC5 | TaCCHC5 | TaCCHC8 | TaCCHC17 |
|  | TaCCHC10 | TaCCHC7 | TaCCHC11 | TaCCHC9 | TaCCHC31 |
|  | TaCCHC11 | TaCCHC8 | TaCCHC16 | TaCCHC13 | TaCCHC37 |
|  | TaCCHC12 | TaCCHC9 | TaCCHC18 | TaCCHC14 | TaCCHC40 |
|  | TaCCHC13 | TaCCHC13 | TaCCHC20 | TaCCHC16 | TaCCHC43 |
|  | TaCCHC14 | TaCCHC14 | TaCCHC21 | TaCCHC18 | TaCCHC48 |
|  | TaCCHC15 | TaCCHC16 | TaCCHC22 | TaCCHC20 |  |
|  | TaCCHC16 | TaCCHC18 | TaCCHC23 | TaCCHC21 |  |
|  | TaCCHC18 | TaCCHC20 | TaCCHC24 | TaCCHC28 |  |
|  | TaCCHC20 | TaCCHC21 | TaCCHC28 | TaCCHC29 |  |
|  | TaCCHC21 | TaCCHC28 | TaCCHC29 | TaCCHC32 |  |
|  | TaCCHC22 | TaCCHC29 | TaCCHC34 | TaCCHC33 |  |
|  | TaCCHC23 | TaCCHC32 | TaCCHC35 | TaCCHC35 |  |
|  | TaCCHC24 | TaCCHC33 | TaCCHC36 | TaCCHC36 |  |
|  | TaCCHC28 | TaCCHC36 | TaCCHC37 | TaCCHC38 |  |
|  | TaCCHC34 | TaCCHC38 | TaCCHC38 | TaCCHC40 |  |
|  | TaCCHC35 | TaCCHC43 | TaCCHC40 | TaCCHC46 |  |
|  | TaCCHC36 | TaCCHC46 | TaCCHC42 | TaCCHC48 |  |
|  | TaCCHC38 | TaCCHC48 | TaCCHC43 | TaCCHC49 |  |
|  | TaCCHC40 | TaCCHC49 | TaCCHC46 |  |  |
|  | TaCCHC42 |  | TaCCHC47 |  |  |
|  | TaCCHC43 |  | TaCCHC48 |  |  |
|  | TaCCHC46 |  | TaCCHC49 |  |  |
|  | TaCCHC47 |  | TaCCHC50 |  |  |
|  | TaCCHC48 |  |  |  |  |
|  | TaCCHC49 |  |  |  |  |
|  | TaCCHC50 |  |  |  |  |

Note: DEG: differentially expressed gene

**Supplementary Table 10.** Primers used for qRT-PCR.

| **Gene Name** | **Forward Primer** | **Reverse Primer** |
| --- | --- | --- |
| TaCCHC1 | TGGTGGATTTGCTTGGGGTT | GCAACCAGACATGTCCCTCA |
| TaCCHC2 | AAGTGGATGGTGGTGACGAC | CCTCCTGACCCTCCTCTTCT |
| TaCCHC3 | GCTGATGAGGCCCGATACAA | CTCGTGAACCACCAGAACCA |
| TaCCHC9 | CACGTCGAAAGAGTCTCGCT | GTGTTGCTGGACGTGAAACC |
| TaCCHC10 | AGTATCACTGCGCTGGACTG | GCACCATCAAGAGCCAATGC |
| TaCCHC11 | CAAGCCCTGCTAACAATGGC | GCCATCATCGTCACCATTGC |
| TaCCHC16 | TGGTTCTGGTGGTTCACGAG | TGTTCTTCCAGTCACCAGCC |
| TaCCHC22 | CACTGGGCTCGTGAATGTCT | TGATCCTCCAAAGCGGTCAC |
| TaCCHC32 | TTTATCGGTGCCCAGCTCAG | ATGTTTTGGTTGGTGGGGGT |
| TaCCHC33 | AAGAGAACCACTGGGCGAAG | GACCAGTGACCAGCCTTACC |
| TaCCHC36 | AAGGGGCTGGTAGAGATCGT | CAAAGTGTCCAGGTTTGCCG |
| TaCCHC37 | GATGGAGGCTCGGATGAAGG | TTGATGGAGGACTGGTGCAC |
| TaCCHC38 | GGATGGCAAGAATGGGTGGA | CATTCTCGTGCAAAGTGGCC |
| TaCCHC46 | ACAGGAGGGATGCTCAGGAT | GACCAGATTCGCCACACTCA |
| TaCCHC47 | CGAGACTATCGTGGTGGTGG | TGCAAAATGACCAGGCTTGC |
| Actin-TaRP15 | GCACACGTGCTTTGCAGATAAG | GCCCTCAAGCTCAACCATAACT |


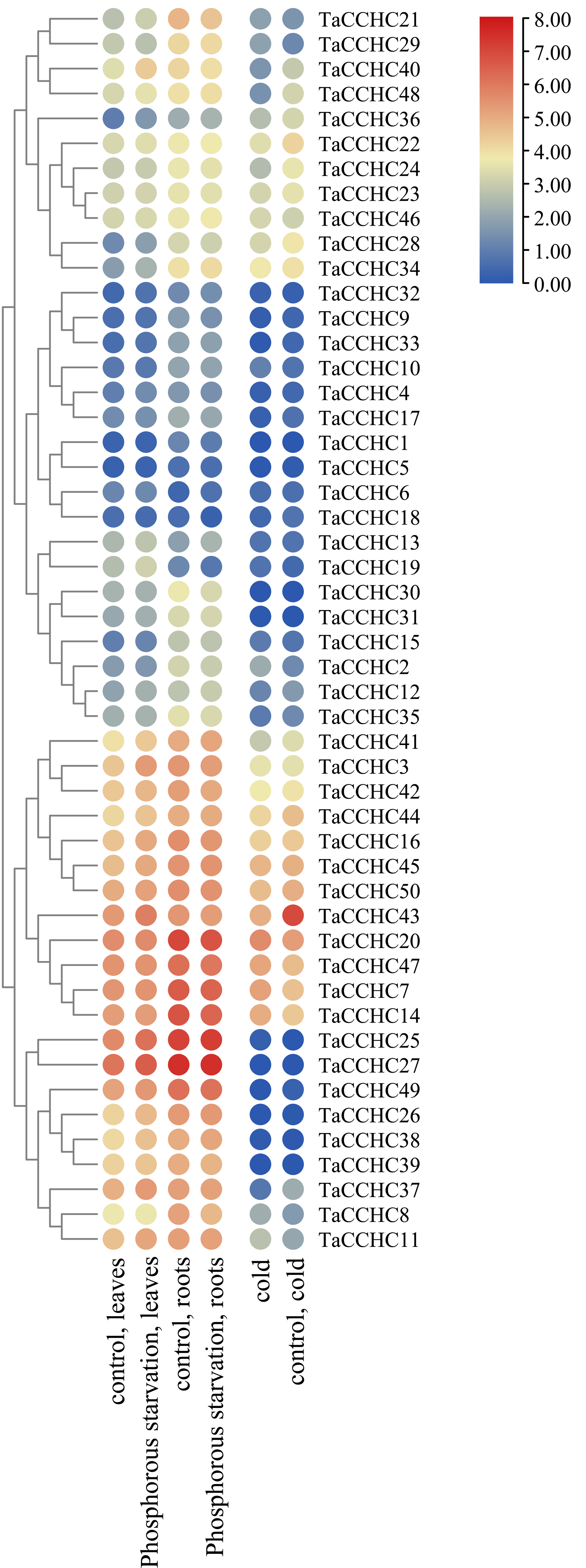


**Supplementary Figure 1.** Expression patterns of 50 TaCCHC-ZF genes under phosphorous starvation and cold treatments.
